# A Large-Scale, Higher-Level, Molecular Phylogenetic Study of the Insect Order Lepidoptera (Moths and Butterflies)

**DOI:** 10.1371/journal.pone.0058568

**Published:** 2013-03-12

**Authors:** Jerome C. Regier, Charles Mitter, Andreas Zwick, Adam L. Bazinet, Michael P. Cummings, Akito Y. Kawahara, Jae-Cheon Sohn, Derrick J. Zwickl, Soowon Cho, Donald R. Davis, Joaquin Baixeras, John Brown, Cynthia Parr, Susan Weller, David C. Lees, Kim T. Mitter

**Affiliations:** 1 Institute for Bioscience and Biotechnology Research, University of Maryland, College Park, Maryland, United States of America; 2 Department of Entomology, University of Maryland, College Park, Maryland, United States of America; 3 Entomology, State Museum of Natural History, Stuttgart, Germany; 4 Laboratory of Molecular Evolution, Center for Bioinformatics and Computational Biology, University of Maryland, College Park, Maryland, United States of America; 5 Florida Museum of Natural History, Gainesville, Florida, United States of America; 6 Department of Ecology and Evolutionary Biology, University of Arizona, Tucson, Arizona, United States of America; 7 Department of Plant Medicine, Chungbuk National University, Cheongju, Korea; 8 Department of Entomology, Smithsonian Institution, Washington, District of Columbia, United States of America; 9 Cavanilles Institute of Biodiversity and Evolutionary Biology, University of Valencia, Valencia, Spain; 10 Systematic Entomology Lab, Agricultural Research Service, United States Department of Agriculture, Beltsville, Maryland, United States of America; 11 Encyclopedia of Life, Smithsonian Institution, Washington, District of Columbia, United States of America; 12 Department of Entomology, University of Minnesota, Saint Paul, Minnesota, United States of America; 13 Department of Life Sciences, Natural History Museum, London, England; Field Museum of Natural History, United States of America

## Abstract

**Background:**

Higher-level relationships within the Lepidoptera, and particularly within the species-rich subclade Ditrysia, are generally not well understood, although recent studies have yielded progress. We present the most comprehensive molecular analysis of lepidopteran phylogeny to date, focusing on relationships among superfamilies.

**Methodology / Principal Findings:**

483 taxa spanning 115 of 124 families were sampled for 19 protein-coding nuclear genes, from which maximum likelihood tree estimates and bootstrap percentages were obtained using GARLI. Assessment of heuristic search effectiveness showed that better trees and higher bootstrap percentages probably remain to be discovered even after 1000 or more search replicates, but further search proved impractical even with grid computing. Other analyses explored the effects of sampling nonsynonymous change only versus partitioned and unpartitioned total nucleotide change; deletion of rogue taxa; and compositional heterogeneity. Relationships among the non-ditrysian lineages previously inferred from morphology were largely confirmed, plus some new ones, with strong support. Robust support was also found for divergences among non-apoditrysian lineages of Ditrysia, but only rarely so within Apoditrysia. Paraphyly for Tineoidea is strongly supported by analysis of nonsynonymous-only signal; conflicting, strong support for tineoid monophyly when synonymous signal was added back is shown to result from compositional heterogeneity.

**Conclusions / Significance:**

Support for among-superfamily relationships outside the Apoditrysia is now generally strong. Comparable support is mostly lacking within Apoditrysia, but dramatically increased bootstrap percentages for some nodes after rogue taxon removal, and concordance with other evidence, strongly suggest that our picture of apoditrysian phylogeny is approximately correct. This study highlights the challenge of finding optimal topologies when analyzing hundreds of taxa. It also shows that some nodes get strong support only when analysis is restricted to nonsynonymous change, while total change is necessary for strong support of others. Thus, multiple types of analyses will be necessary to fully resolve lepidopteran phylogeny.

## Introduction

Among the largest of insect orders, the Lepidoptera, with more than 157,000 described species [1], serve terrestrial ecosystems as major herbivores, pollinators, and prey [2]. They have major impact on humans as agricultural pests, but also provide important model systems for scientific enquiry [3]. However, the complexity and abundance of their interactions with the rest of the natural environment is not easily captured across space and time through the study of model systems alone. A robust phylogeny would provide a valuable framework for the analysis of large-scale environmental and evolutionary processes and patterns exemplified by Lepidoptera.

**Figure 1:**
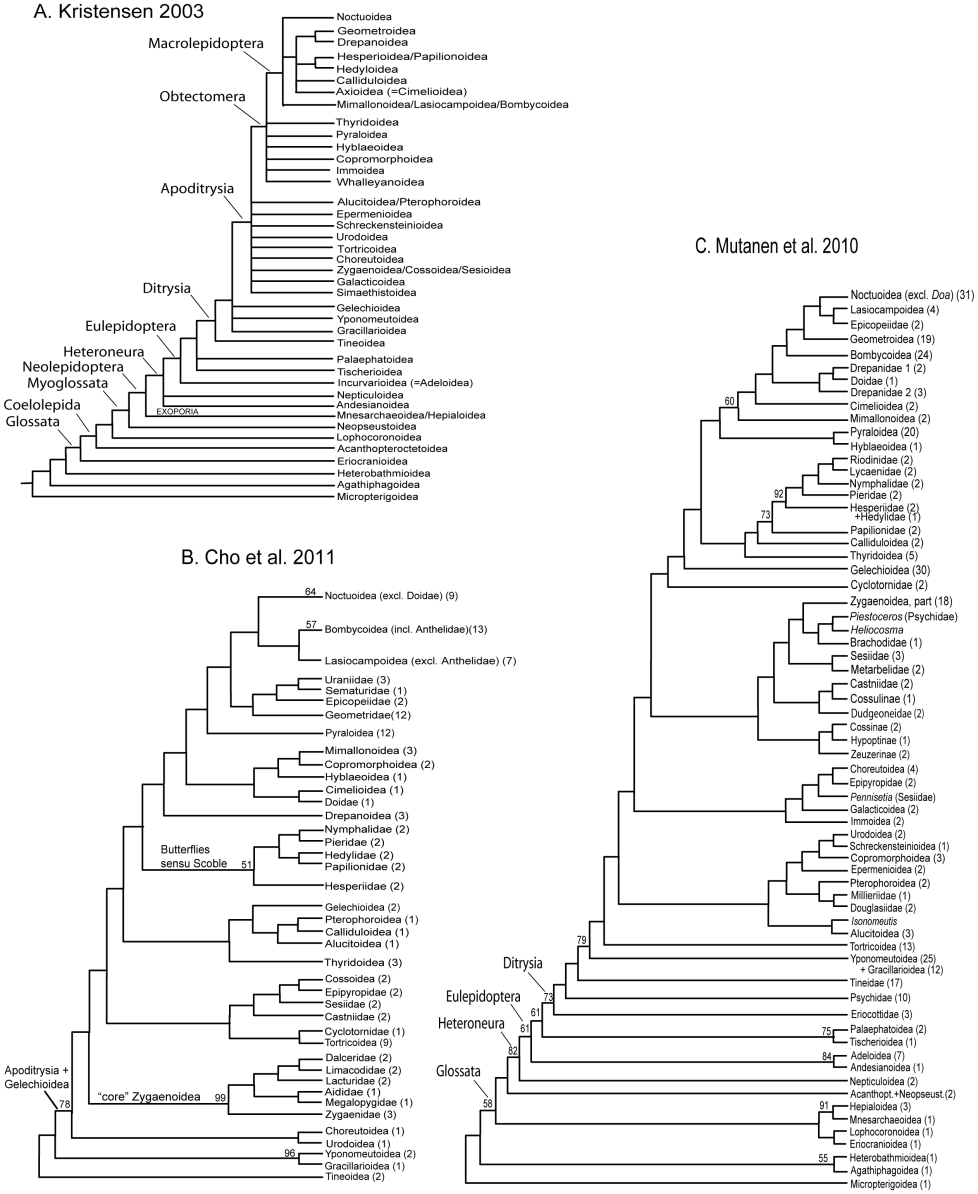
Previous hypotheses of deep-level relationships in Lepidoptera. A. Composite working hypothesis based on morphology [7]. B. Ditrysian-only relationships (rooted on Tineoidea) inferred from *degen1* ML analysis of 123 taxa sequenced either for 5 or 26 gene segments, with bootstrap values ≥50% displayed for nodes at the superfamily level and above [6]. C. Lepidopteran relationships (rooted on Micropterigoidea) inferred from ML analysis of 350 taxa, using nucleotides from the first and second codon positions (+ third codon position for EF-1α only) of 8 gene segments, with bootstrap values >50% displayed for nodes at the superfamily level and above [5]. Numbers in parentheses after taxon names are numbers of exemplars sampled.

The current report, which builds on other recent studies ([4]–[6]; Figure 1), describes our search for robust support of higher-level lepidopteran relationships, particularly across families and superfamilies. In this effort, we have extensively sampled extant lepidopteran diversity -- 483 species representing 45 of 47 superfamilies, 115 of 124 families, and 303 of 332 subfamilies in the classification system of Kristensen [7]. In parallel, we [8]–[11] and others [12]–[15] have also begun a systematic description of intra-superfamily relationships, often obtaining robust support. Already, however, it is apparent that, generally speaking, higher-level lepidopteran relationships are more challenging to decipher than lower-level relationships, based on broadly weak support across the backbone of the lepidopteran tree in multiple multi-gene studies [4]–[6]. This may be because the earlier lepidopteran radiations, particularly within the clade Ditrysia, which constitutes approximately 98% of extant species diversity, have been rapid and occurred mostly by the Cretaceous. Rapid radiations typically give rise on phylograms to short internal branches, reflecting reduced phylogenetic signal. Generating sufficient phylogenetic signal to yield statistically significant support for these short branches can be challenging. This challenge is compounded when the nodes of interest subtend relatively long terminal branches, making the "multiple hits" problem more acute. Such is the case for the Ditrysia and likely for many other insect radiations [16].

Multiple strategies have been devised to deal with these challenges, and we have incorporated these into the current study. One strategy is to increase the size of the data set. The current study samples up to 19 protein-coding nuclear genes for each taxon, while our earlier 123-taxon study utilized only five [4]. A second strategy is to utilize the increasing availability of grid computing to enable more, and more thorough, heuristic searches. For the current study, grid computing has provided at least a hundred-fold increase in search capacity relative to some of our earlier studies (cf. [17] versus [4]). These first two strategies can only be implemented if, as a third strategy, suitable phylogenetic software is available and utilized. The current study takes advantage of the now grid-operable GARLI program [18],[19], which, when performing maximum likelihood and bootstrap analyses, has already proved valuable for lepidopteran and arthropod phylogeny [4], [6], [8], and in a manner that does not tend towards overconfidence in interpreting node support values [20].

A fourth strategy is based on distinguishing synonymous and nonsynonymous change in character codings. For relatively recent lepidopteran divergences, say, within many families (e.g., [21], synonymous change, which accumulates more rapidly, provides an abundance of useful phylogenetic information, while that from the typically more slowly evolving nonsynonymous change is sparse. By contrast, for resolving Paleozoic- and Mesozoic-aged clades across Arthropoda, synonymous change is almost completely undecipherable due to multiple overlapping substitutions, and can even become misinformative due to evolving compositional heterogeneity, while nonsynonymous change can now contribute much useful signal, and remains less prone to compositional heterogeneity over this period [22]–[25]. In terms of divergence times, higher-level lepidopteran relationships likely present an intermediate situation, one in which both synonymous and nonsynonymous change are potentially useful, although not necessarily at the same nodes. For example, we have recently shown that nonsynonymous change provides strong support for a novel higher-level taxonomic group near the base of Ditrysia, namely, 'Ditrysia − (Tineoidea, Gracillarioidea, Yponomeutoidea)', but that overall support for this group largely disappears if synonymous change is included [6]. By contrast, the level of support for another higher-level grouping (i.e., 'Noctuoidea − Doidae'), although not as high, is significantly greater when synonymous change is included. Unfortunately, most backbone nodes within Ditrysia receive little support under either condition; hence, our speculation about rapid radiations. The current report provides a more elaborate and definitive test of the differential utilities of synonymous and nonsynonymous change, and particularly of the (still controversial) hypothesis that analysis of nonsynonymous change alone can yield improved confidence of some higher-level lepidopteran groups. The novelty of the current test resides in an almost fourfold increase in number of taxa sampled and a doubling of the amount of sequence per taxon.

## Results

### On recovering the maximum-likelihood topology

**Figure 2:**
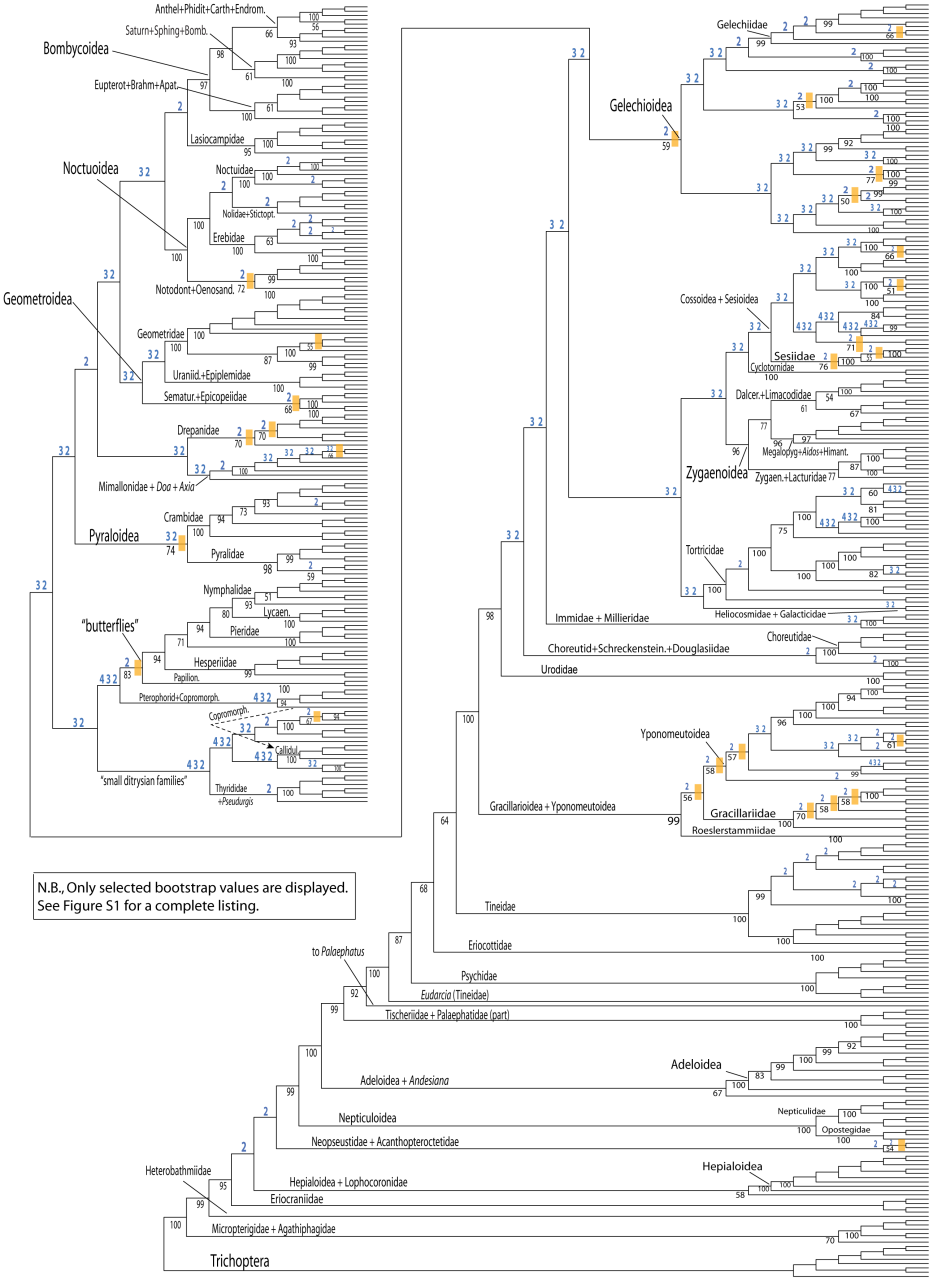
Assessing the effectiveness of the GARLI heuristic ML search through an analysis of 4608 search replicates as derived from the full 483-taxon, 19-gene, *nt123_degen1* data set. Out of 4608 search replicates, the single fully-resolved topology of highest likelihood is displayed (lnL  =  −583,900.053394). Terminal taxa, not shown in this figure in order to save space, are displayed in Figure S1. Dichotomous nodes that are not present in one or more strict consensuses of subsets of the 4608 topologies are identified by having numbers with blue coloration *above* subtending branches. The three subsets are as follows: *4*, all topologies with lnL scores that are within 0.0001% (10^-4^ %) of that of the best ML topology (2 topologies total, including the best ML topology); *3*, all topologies within 0.001% (10^-3^ %, 19 topologies total); *2*, all topologies within 0.01% (10^-2^ %, 1827 topologies total). Selected bootstrap percentages based on 15 heuristic search replicates and 500 bootstrap pseudoreplicates that are ≥50% are displayed *below* branches (see Figure S1 for all bootstrap percentages). An orange-colored bar is placed beside each node that has bootstrap support ≥50% and that is missing in one or more of the subset consensuses. The dashed arrow identifies the altered placement of one (and only one) taxon that was found in a new and improved topology (lnL  =  −583,898.838616), when the dichotomous topology displayed in this figure was used as a starting tree in a second round of 561 GARLI ML heuristic searches. This new topology was recovered in 248 of the 561 search replicates. Higher-level taxon names, some of which are abbreviated, are displayed. All abbreviations follow: Anth+Phidit+Carth+Endrom., Anthelidae + Phiditidae + Carthaeidae + Endromidae; Saturn+Sphing+Bomb., Saturniidae + Sphingidae + Bombycidae; Eupterot+Brahm+Apat., Eupterotidae + Brahmaeidae + Apatelodidae; Nolidae+Stictopt., Nolidae + Stictopterinae; Notodont+Oenosand., Notodontidae + Oenosandridae; Uraniid.+Epiplemidae, Uraniidae + Epiplemidae; Sematur.+Epicopeiidae, Sematuridae + Epicopeiidae; Papilion., Papilionidae; Pterophorid+Copromorph., Pterophoridae (part) + Copromorphidae (part); Copromorph., Copromorphidae (part); Callidul., Callidulidae; “small ditrysian families”, Copromorphidae + Carposinidae + Epermeniidae + Alucitidae + Hyblaeidae + Pterophoridae (part) + Thyrididae + *Pseudurgis* (unplaced); Dalcer.+Limacodidae, Dalceridae + Limacodidae; Megalopyg+Aidos+Himant., Megalopygidae + *Aidos* + Himantopteridae; Zygaen.+Lacturidae, Zygaenidae + Lacturidae; Choreutid+Schreckenstein.+Douglasiidae, Choreutidae + Schreckensteiniidae + Douglasiidae.

With 483 taxa in the present study, a heuristic, rather than exhaustive, search for the topology of highest likelihood is a practical necessity. Previously [4], we described and utilized a metric to estimate how many search replicates would be required in order to have 95% confidence that the recovered topology of highest likelihood is the "best-feasible" topology. This metric is based on the frequency of recovering the topology of apparent highest likelihood. However, after performing 4608 search replicates on the full 483-taxon, 19-gene, *nt123_degen1* data set (see Materials and Methods for a description of the *degen1* approach), even the top two trees differed -- at 13 out of the 481 internal nodes (Figure 2) -- so a confidence estimate could not be assigned. Instead, we extended our search for an improved topology by using the tree of highest likelihood (lnL  =  -583,900.053394) from the 4608 searches as a starting tree for 561 additional search replicates. Now, a new best topology (lnL  =  -583,898.838616) was recovered 248 times that differs from the starting topology in the placement of only one taxon, although multiple nodes in that highly localized region of the tree are thereby affected relative to the starting topology (Figure 2). It is reasonable to expect that this new topology would have been the best-feasible topology if the original search had been extended, although of course we have not demonstrated this.

An interesting aspect of the search for an overall best-feasible *degen1* topology is that the recovery of some nodes requires, on average, more search replicates of the full data set than others. To illustrate this, we have compared strict consensuses of subsets of the 4608 topologies with lnL scores that are within 10^-4^ %, 10^-3^ %, and 10^-2^ % of the best topology, that is, the one shown in Figure 2. The number of such topologies (always including the best one) are 2, 19, and 1827, respectively. Of particular relevance to this report is that many of the high-interest backbone nodes in Ditrysia are relatively difficult to recover; that is, they are not recovered in the strict consensus of the top 10^-3^ % of all topologies. As a correlate, many hard-to-recover nodes, including all of those along the backbone, have low (i.e., <50%) bootstrap support, but elsewhere in the tree there are a few examples of nodes with low bootstrap support that are not hard-to-recover, and there are numerous examples of difficult-to-recover groups that do have bootstrap ≥50%, so the correlation with bootstrap support is variable. For example, of taxonomic groups found in the best topology (Figure 2), Pyraloidea and butterflies have the highest bootstrap percentages (namely, 74 and 83, respectively) of any group that is *not* present in all topologies of the top 10^-3^ % and 10^-2^ %, respectively. None of the 13 nodes that differ between the top two topologies (i.e., the top 10^-4^ %) have bootstraps ≥50%.

A less-extensive analysis of the 483-taxon, 19-gene, *nt123* data set (based on 977 search replicates) again demonstrates the challenge of finding a best-feasible topology. For example, a strict consensus of the three topologies within 10^-4^ % of the best topology (lnL  =  -2,429,912.231878) has eight collapsed nodes (results not shown).

### On calculating bootstrap percentages

**Figure 3:**
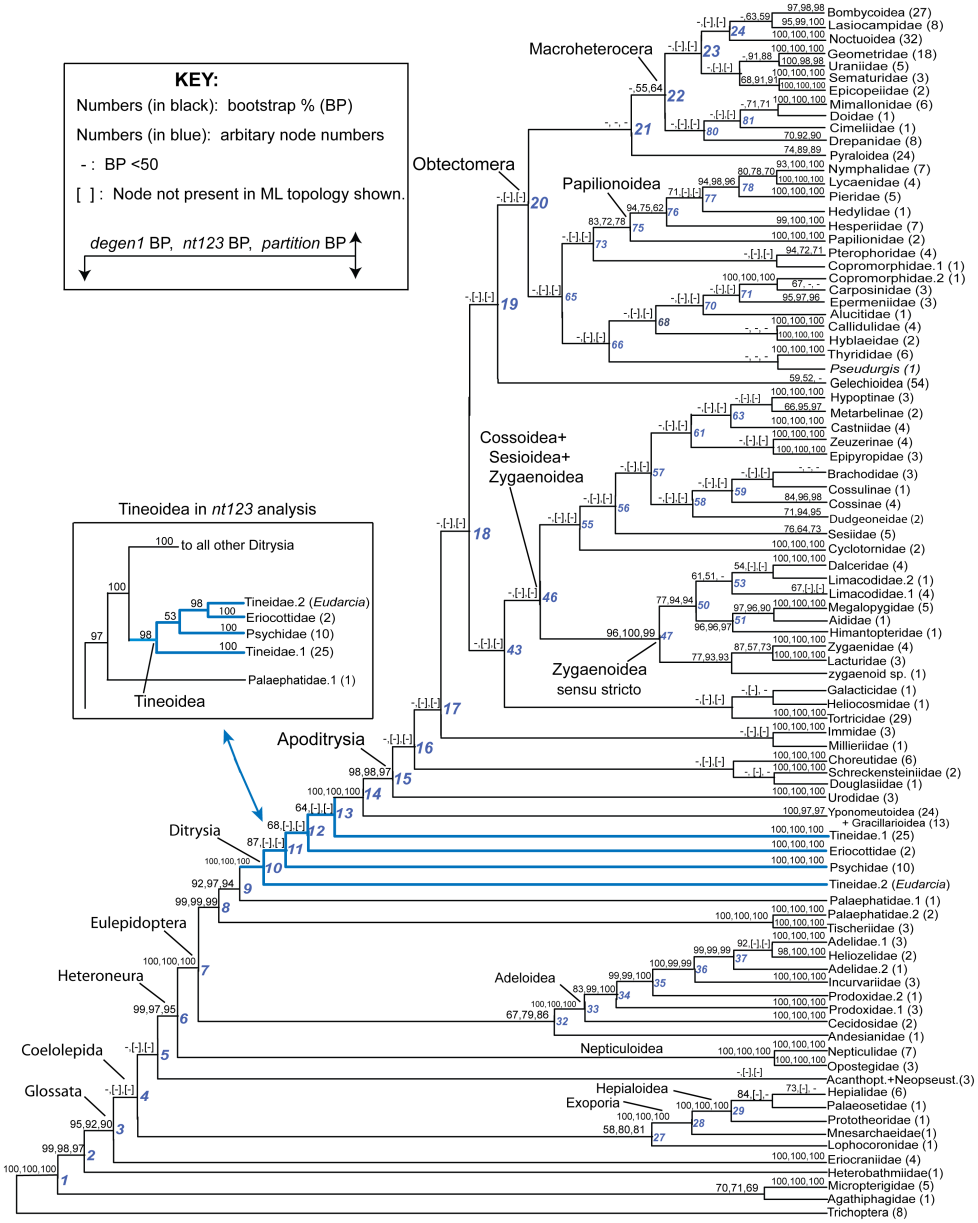
Summary of three phylogenetic analyses of 483 taxa and 19 genes. Bootstrap percentages derived from GARLI analysis of three data sets -- *nt123_degen1*, *nt123, and nt123_partition* -- are displayed in that order above internal branches of a condensed, higher-level-only portion of the *nt123_degen1* ML topology (see numbers in black). Selected nodes are arbitrarily numbered for convenient reference (see numbers in blue). The full *nt123_degen1* and *nt123* topologies are shown in Figure S1 and S2, respectively. A bracket indicates that the node displayed was not recovered in the ML analysis of that data set. A dash indicates that the bootstrap value is <50%. The number of exemplars is listed in parentheses after the family or subfamily name. The region of the topology that includes Tineoidea has blue-colored branches, and its favored alternative topology, based on analysis of *nt123*, is also displayed (see lower boxed area). Throughout this report, we have subsumed all tineoid taxa traditionally identified as Acrolophidae under Tineidae, all tineoid taxa traditionally identified as Arrhenophanidae under Psychidae, and Crinopterygidae under Incurvariidae, following van Nieukerken et al. [1]. *BP*, bootstrap percentage.

Two factors were considered in the design of our bootstrap analyses. Firstly, we settled on performing approximately 500 bootstrap pseudoreplicates per analysis, which should yield a standard error of ≤5% around a true value for those bootstrap percentages in the range of 60% and greater [26]. Secondly, we undertook a pilot study to empirically estimate how many search replicates would be needed to ensure an adequate search for each bootstrap pseudoreplicate, that is, to determine the number of search replicates beyond which there was no significant increase in the bootstrap percentage. To do this, we performed 15 and 25 search replicates per bootstrap pseudoreplicate for the *nt123_degen1* and *nt123* data sets, respectively, and then subsampled the resulting topologies to varying extents (1, 5, 10, and 15 replicates for *nt123_degen1*; 1, 5, 10, 15, and 25 replicates for *nt123*). For both data sets, the majority of nodes with bootstrap ≥50% showed no sensitivity to increasing numbers of search replicates (± 5%), indicating that even a single search replicate per bootstrap pseudoreplicate was adequate. However, there were 15 and 22 nodes for *nt123_degen1* and *nt123*, respectively, whose bootstrap values significantly increased up to 5 search replicates, and 4 and 7 nodes for *nt123_degen1* and *nt123*, respectively, that further increased up to 10 search replicates (Tables 1, 2; Figure 3). Based on these findings, we performed 15 search replicates per bootstrap pseudoreplicate for all other analyses reported herein, except for the one mentioned immediately below.

**Table 1:** Assessing the effectiveness of the GARLI heuristic bootstrap search by varying the number of search replicates performed per individual bootstrap pseudoreplicate in an analysis of 500 483-taxon, 19-gene, *nt123_degen1*, bootstrapped data sets.*

		Number of search replicates per bootstrap pseudoreplicate			
Node number	Taxonomic group	**1**	5	10	15
75	"butterflies"	***76***	81	82	83
76	"butterflies" − Papilionidae	***88***	93	94	94
53	Zygaenoidea subgroup *A* (9 taxa)	***56***	62	61	61
50	Zygaenoidea subgroup *B* (16 taxa)	***67***	*72*	77	77
	Zygaenoidea subgroup *C* (7 taxa)	***82***	87	87	87
	Zygaenoidea subgroup *D* (8 taxa)	***71***	78	77	78
47	Zygaenoidea sensu stricto	***73***	***89***	95	96
	Pyraloidea	***69***	73	74	74
	Gelechioidea	***50***	55	59	59
	Gelechioidea subgroup (7 taxa)	***94***	99	99	100
	Pterophoridae (4 taxa)	***85***	94	93	94
	Epermeniidae (3 taxa)	***56***	*81*	90	95
	Cossidae subgroup (3 taxa)	***95***	100	100	100
	Brachodidae subgroup (2 taxa)	***81***	*94*	98	99
15	Ditrysia − (Tineoidea, Gracillarioidea, Yponomeutoidea)	***92***	96	97	98

* Bootstrap percentages of all taxonomic groups in Figures 3 and S1 that are at least 5% lower than the value for 15 search replicates are displayed in this table in boldfaced, italicized font (columns 3–6). In no case was the value for 1 search replicate higher than that for 15 by 5% or more. Only bootstrap percentages close to or over 60% at 15 search replicates, and which differ by 5% or more from corresponding values at 1 search replicate, are shown in this table. Node numbers (column 1) refer to correspondingly numbered nodes in Figure 3, while un-numbered taxonomic groups correspond to terminal taxa in that same figure.

**Table 2:** Assessing the effectiveness of the GARLI heuristic bootstrap search by varying the number of search replicates performed per individual bootstrap pseudoreplicate in an analysis of 500 483-taxon, 19-gene, *nt123*, bootstrapped data sets.*

		Number of search replicates per bootstrap pseudoreplicate				
Node number	Taxonomic group	1	5	10	15	25
14	Ditrysia − Tineoidea	***89***	100	100	100	100
	Tineoidea	***92***	98	98	98	97
15	Ditrysia − (Tineoidea, Gracillarioidea, Yponomeutoidea)	***83***	97	98	99	98
	Gracillarioidea + Yponomeutoidea	***90***	97	98	98	97
	Dudgeoneidae (2 taxa)	***88***	93	94	94	95
	Epermeniidae	***75***	***88***	93	94	97
	Sesiidae	***59***	62	63	62	64
	Pterophoridae subgroup (4 taxa)	***51***	***60***	68	70	72
	Choreutidae	***81***	***94***	99	100	100
	Mimallonidae + *Doa*	***63***	69	69	70	71
	Drepanidae	***87***	90	91	91	92
	Gelechioidea subgroup *A* (4 taxa)	***91***	96	98	98	98
	Gelechioidea subgroup *B* (6 taxa)	***91***	96	98	98	98
	Gelechioidea subgroup *C* (8 taxa)	***77***	83	87	86	86
	Gelechioidea subgroup *D* (12 taxa)	***76***	79	82	82	81
	Cosmopterigidae subgroup (2 taxa)	***75***	78	79	79	80
	Pyraloidea	***74***	***83***	86	87	89
	Pyralidae	***94***	98	100	100	100
75	"butterflies"	***59***	***66***	68	69	72
	Geometridae + Uraniidae	***64***	***83***	88	90	91
	Uraniidae	***74***	***92***	96	97	98
	Notodontidae + Oenosandridae	***73***	77	78	77	78

* Bootstrap percentages of all taxonomic groups in Figures 3 and S2 that are at least 5% lower than the value for 15 search replicates are displayed in this table in boldfaced, italicized font (columns 3–7). In no case was the value for 1 search replicate higher than that for 15 by 5% or more. Only bootstrap percentages close to or over 60% at 25 search replicates, and which differ by 5% or more from corresponding values at 1 search replicate, are shown in this table. Node numbers (column 1) refer to correspondingly numbered nodes in Figure 3, while un-numbered taxonomic groups correspond to terminal taxa in that same figure.

Near the end of this entire study, we revisited the question as to how many search replicates were required to generate accurate bootstrap values by repeating the bootstrap analysis of the 483-taxon, 19-gene *nt123_degen1* data set but increasing the number of search replicates to 1000 for each of 505 bootstrap pseudoreplicates. If our initial conclusion were correct, namely, that effort beyond 15 search replicates would not significantly increase bootstrap values, we would expect, for one, that in the new analysis with 1000 search replicates per bootstrap pseudoreplicate, only about 5% of nodes would show differences in bootstrap support from the initial analyses greater than 5% points (and then probably not too much beyond 5% points), and, for another, that these differences would be negative as often as positive. While the frequency of nodes with bootstrap difference between the two analyses ≥ 5% points was indeed on the order of 5% (17/482  =  3.5%), for all but one of such nodes (16/17 = 94%), the search with 1000 search replicates per bootstrap pseudoreplicate gave the higher value (Table 3). Of the nodes with bootstrap values from 50–79% in the initial analysis with 15 search replicates, two showed increases of 6 percentage points or less, while five showed increases from 11–23 points. There were three nodes with BP <50% after 15 search replicates but with BP >50% after 1000 search replicates, showing increases of 17, 30, and 40 percentage points. These results strongly suggest that bootstrap support for at least some nodes in the initial analysis was underestimated due to insufficient search effort.

**Table 3:** A further assessment of the effectiveness of the GARLI heuristic bootstrap search by instituting a huge increase in the number of search replicates performed per individual bootstrap pseudoreplicate in an analysis of 505 483-taxon, 19-gene, *nt123_degen1*, bootstrapped data sets.*

		Numbers of search replicates /bootstrap pseudoreplicate		
Node number	Taxonomic group	15	1000	% points difference
	Lasiocampidae	95	100	+ 5
	***Macroheterocera + Pyraloidea + Hyblaeidae***	31	71	+ 40
75	butterflies	83	88	+ 5
	Nymphalidae	93	98	+ 5
	Epermeniidae	95	100	+ 5
	***Callidulidae + Copromorphidae:Copromorpha***	36	66	+ 30
	***Sesiidae***	76	95	+ 19
	***Cossidae:Metarbelinae***	66	89	+ 23
50	***Dalceridae + Limacodidae + Megalopygidae + Aididae + Himantopteridae***	77	88	+ 11
	Zygaenidae + Lacturidae	87	93	+ 6
	***Zygaenidae + Lacturidae + 'zygaenoid sp. (Lact)'***	77	89	+ 12
16	***Apoditrysia − Urodidae***	40	57	+ 17
13	Apoditrysia + Yponomeutoidea + Gracillarioidea + Tineidae (no *Eudarcia*)	64	70	+ 6
12	***Apoditrysia + Yponomeutoidea + Gracillarioidea + Tineidae (no Eudarcia) + Eriocottidae = 'Ditrysia − (Psychidae, Arrhenophanidae, Eudarcia)'***	68	79	+ 11
11	Apoditrysia + Yponomeutoidea + Gracillarioidea + Tineidae (no *Eudarcia*) + Eriocottidae + Psychidae + Arrhenophanidae = 'Ditrysia − *Eudarcia*'	87	92	+ 5
	'Adelidae − *Nematopogon'* + Heliozelidae	92	99	+ 7
	Micropterigidae + Agathiphagidae	70	65	− 5

* Node numbers (column 1) refer to correspondingly numbered nodes in Figure 3, while un-numbered taxonomic groups either correspond to terminal taxa in that same figure or to groups not recovered. Numbers in columns 3 and 4 are the resulting bootstrap percentages. Taxonomic groups whose bootstrap percentage increases by >10% are in boldfaced, italicized font (column 2).

### Three approaches to phylogeny estimation

Maximum likelihood and bootstrap analyses were performed on the *nt123_degen1, nt123*, and *nt123-partition* data sets. For ease of presentation, bootstrap values for all three data sets have been mapped onto the higher-level phylogeny provided by the *degen1* maximum-likelihood estimate (Figure 3, but see Figures S1, S2 for the complete *degen1* and *nt123* results mapped onto their own maximum-likelihood topologies in phylogram format). Note that for those nodes in the *degen1* ML topology that are not present in the *nt123* and *nt123-partition* ML topologies, the bootstrap percentages of the *nt123* and *nt123-partition* results are in brackets. There are numerous regions of the tree where bootstrap percentages vary significantly between *degen1* and *nt123* or *nt123-partition*, but for deep-level relationships it is only Tineoidea and relationships therein where they also strongly conflict (see below and Discussion). Multiply-sampled families and some superfamilies are generally strongly supported by one or more approaches, as are many backbone relationships at the base of Lepidoptera, i.e., outside Apoditrysia. However, within Apoditrysia backbone relationships are uniformly weakly supported. An examination of the phylograms for *degen1* and *nt123* (Figures S1, S2, respectively) reveals that many of the weakly supported backbone relationships have short basal branches, consistent with little informative change.

### Taxon subsampling as an approach for increasing node support

Three general taxon subsampling schemes of the *nt123_degen1* and *nt123* data sets were explored in varying combinations: 1) removal of "rogue" taxa (defined by two approaches, see Materials and Methods), 2) removal of compositionally heterogeneous taxa, and 3) removal of distant outgroups (see Text S1 for listing of taxa deleted). Of most interest are 21 supra-family-level groups whose bootstrap support in one or more subsampling schemes increases by at least 5% points relative to that in the 483-taxon data set, and always to values ≥50% (Tables 4, 5; see Tables S3XPATH ERROR: unknown variable "checknextn".). In 11 of these, bootstrap support becomes strong, i.e., ≥80%, under the particular subsampling scheme. Examples are a modified Macroheterocera (up to 79% for *degen1* and 88% for *nt123*), placement of the "noctuoid" *Doa* (Doidae) with the non-noctuoid family Mimallonidae (up to 92% for *nt123*), and grouping of Cossoidea (including Castniidae), Sesioidea, and all or part of Zygaenoidea (up to 96% for *nt123*). (See also Discussion below.)

**Table 4:** Selected bootstrap results based on analysis of taxon-depleted *nt123_degen1* data sets.*

Node number	Taxonomic Group	483 taxa	453 taxa, no ACrogue	436 taxa, no RNRrogue	434 taxa, no RNRrogue, − Acan, − Neop: *Neopseustis*	344 taxa, APODIT	314 taxa, APODIT, no ACrogue	133 taxa, MACRO	129 taxa, MACRO, no ACrogue
	Bomb + Lasi	36	52	25	24	40	54	x	38
	Geom:Sema + Drep:Epic	68	***72***	***74***	***76***	***70***	61	66	59
22	Bomb + Lasi + Noct + Drep + Geom + Mima + Cime ( = **MACRO**)	39	63 [no *Doa*, Cime]	*79* [no *Doa*, Cime]	***79***	60	***77*** [no *Doa*, Cime]		
	Mima + *Doa*	33	[no *Doa*]	[no *Doa*]	[no *Doa*]	44	[no *Doa*]	57	[no *Doa*]
	Pyra	***74***	***74***	***70***	*80*	***71***	*77*		
21	**MACRO** + Pyra	23	38	36	37	41	40		
	**MACRO** + Pyra + Hybl	31	27	39	26	34	24		
	Gele	59	*99*	*99*	*99*	68	***99***		
19	**MACRO** + Pyra + Hybl + Copr + Eper+ Thyr + Call + "butterflies" + Pter + Aluc + Gele ( = OBTECT + Gele)	14	65	58	57	16	34		
71	Eper + Copr − *Copromorpha*	35	69 (no *Copromorpha*)	***76*** (no *Copromorpha*)	***73*** (no *Copromorpha*)	37	54 (no *Copromorpha*)		
	Tort + Immo	x	44	32	x	x	x		
46	Zyga + Sesi + Coss	x	25 [no Zyga:Cycl, Zyga: Epip]	x	3	2 [no Zyga:Cycl, Zyga:Epip]	23 [no Zyga:Cycl, Zyga:Epip]		
	Sesi + Coss	x	67	x	x	x	64		
	Schr + Grac:Doug	37	[no Grac:Doug]	***78***	***77***	39	[no Grac:Doug]		
15	Ditrysia − (Urod, Ypon, Grac, Tine) ( = **APODIT**)	40		56	57				
32	Adel + Ande	67		66	68				
	Acan + Neop	35		***76*** [no Neop: *Apoplania*]	[no Acan or Neop]				
	Acan + Neop + Erio	x		45 [no Neop: *Apoplania*]	[no Acan or Neop]				
	Eulep + Neop + Acan + Erio	26		33 [no Neop: *Apoplania*]	52 [no Acan or Neop]				
27	Hepi + Mnes + Loph	58		***72***	***92***				
4	Ditrysia + Tisc + Pala + Adel + Ande + Nept + Acan + Neop + Hepi + Mnes + Loph	28		x	x				
	Agat + Microp	***70***		***77***	***76***				

* Bootstrap results in PAUP* are those shown under the "le  =  yes" option. *x*, Not present in the bootstrap table under le option, so value <50%; "Strong" bootstrap values, i.e., ≥80%, and "moderate" bootstrap values, i.e., 70–79%, are in boldfaced, italicized font (columns 3–10). Node numbers (column 1) refer to correspondingly numbered nodes in Figure 3. Abbreviated taxonomic group names are in column 2 and throughout the table (see below for abbreviations). Columns 3–10 show the bootstrap values based on analysis of eight different *nt123_degen1* data sets. Descriptors of these data sets (see labels at top) include number of taxa (129 – 483), whether rogue taxa were excluded, and whether analysis was restricted to a subset of total Lepidoptera (i.e., *APODIT*, restricted to Apoditrysia; *MACRO*, restricted to Macroheterocera). *ACrogue*, Adams-consensus rogue; *RNRrogue*, RNR rogue; *Bomb*, Bombycoidea; *Lasi*, Lasiocampidae; *Geom:Sema*, Geometroidea:Sematuridae; *Drep:Epic*, Drepanoidea:Epicopeiidae; *Noct*, Noctuoidea; *Drep*, Drepanoidea; *Geom*, Geometroidea; *Mima*, Mimallonidae; *Cime*, Cimeliidae; *MACRO*, Macroheterocera; *Doa*, Noctuoidea: *Doa* sp.; *Pyra*, Pyraloidea; *Hybl*, Hyblaeidae; *Gele*, Gelechioidea; *Copr*, Copromorphoidea; *Eper*, Epermeniidae; *Thyr*, Thyrididae; *Call*, Callidulidae; *"butterflies"*, Nymphalidae + Lycaenidae + Pieridae + Hedylidae + Hesperiidae + Papilionidae; *Pter*, Pterophoridae; *Aluc*, Alucitidae; *Gele*, Gelechioidea; OBTECT, Obtectomera; *Copromorpha*, *Copromorpha* sp.; *Tort*, Tortricoidea; *Immo*, Immoidea; *Zyga*, Zygaenoidea; *Sesi*, Sesioidea; *Coss*, Cossoidea; *Zyga:Cycl*, Zygaenoidea:Cyclotornidae; *Zyga:Epip*, Zygaenoidea:Epipyropidae; *Schr*, Schreckensteiniidae; *Grac:Doug*, Gracillarioidea:Douglasiidae; *Ditrysia*, Ditrysia (as defined in Figure S1); *Urod*, Urodidae; *Ypon*, Yponomeutoidea; *Grac*, Gracillarioidea; *Tine*, Tineoidea; *APODIT*, Apoditrysia; *Adel*, Adeloidea; *Ande*, Andesianidae; *Acan*, Acanthopteroctetidae; *Neop*, Neopseustidae; *Erio*, Eriocraniidae; *Neop:Apoplania*, Neopseustidae:*Apoplania* sp.; *Tisc*, Tischeriidae; *Pala*, Palaephatidae; *Eulep*: Ditrysia + Palaephatidae + Tischeriidae + Adeloidea + Andesianidae; *Erio*, Eriocraniidae; *Hepi*, Hepialidae; *Mnes*, Mnesarchaeidae; *Loph*, Lophocoronidae; *Nept*, Nepticuloidea; *Agat*, Agathiphagidae; *Micr*, Micropterigidae.

**Table 5:** Selected bootstrap results based on analysis of taxon-depleted *nt123* data sets.*

Node number	Taxonomic Group	483 taxa	455 taxa, no ACrogue	432 taxa, no RNRrogue	356 taxa, no RNRrogue, − heterog	344 taxa, APODIT	316 taxa, APODIT no ACrogue	133 taxa, MACRO	129 tx, MACRO, no ACrogue
	Bomb + Lasi	63	*95*	***97***	***99***	54	***96***	***76***	***82***
	Mima + *Doa* + Geom:Sema + Drep:Epic	26	x	x	x	38	62	x	65 [no *Doa*]
	Geom:Sema + Drep:Epic	***91***	***88***	***97***	***91***	***94***	***76***	***94***	68
22	Bomb + Lasi + Noct + Drep + Geom + Mima + Cime ( = **MACRO**)	55	36 [no *Doa*, Cime]	*82* [no Cime]	*88* [no Cime]	*83*	*70* [no *Doa*, Cime]		
	Mima + *Doa*	***71***	[no *Doa*]	***88***	***92***	***85***	[no *Doa*]	***85***	[no *Doa*]
	Pyra	***89***	***95***	*98*	***97***	***95***	***78***		
21	**MACRO** + Pyra	37	23	**58**	68	58	47		
	Gele	52	29	68	***72***	52	54		
19	MACRO + Pyra + Hybl + Copr + Eper+ Thyr + Call + butterflies + Pter + Aluc + Gele ( = OBTECT + Gele)	x	x	x	x	x	x		
	Call + Hybl + Thyr	x	59	***79***	***72***	x	40		
	Call + Hybl	24	*83*	***70***	68	x	69		
71	Eper + Copr − *Copromorpha*	x	17 (no *Copromorpha*)	x	21 (no *Copromorpha*)	14	13 (no *Copromorpha*)		
	Tort + Immo	53	*84*	***72***	***92***	60	***94***		
46	Zyga + Sesi + Coss	2	*96* [no Zyga:Cycl, Zyga:Epip]	32	41	x	***95*** [no Zyga:Cycl, Zyga:Epip]		
32	Adel + Ande	***79***		***83***	***90***				
	Acan + Neop	x		[no Neop]	[no Neop]				
	Acan + Neop + Erio	x		***88** [no Neop]*	***70*** [no Neop]				
	Eulep + Nept + Acan + Neop + Erio	x		***88** [no Neop]*	49 [no Neop]				
27	Hepi + Mnes + Loph	***80***		***100***	[no Loph]				
4	Eulep + Nept + Acan + Neop + Hepi + Mnes + Loph	x		x	x				
	Agat + Microp	***71***		***72***	***81***				

* Bootstrap results in PAUP* are those shown under the "le  =  yes" option. Node numbers (column 1) refer to correspondingly numbered nodes in Figure 3. "Strong" bootstrap values, i.e., ≥80%, and "moderate" bootstrap values, i.e., 70–79%, are in boldfaced, italicized font (columns 3–10). See footnote to Table 4 for definitions of abbreviations.

### Compositional heterogeneity and resolution of the Tineoidea

**Figure 4:**
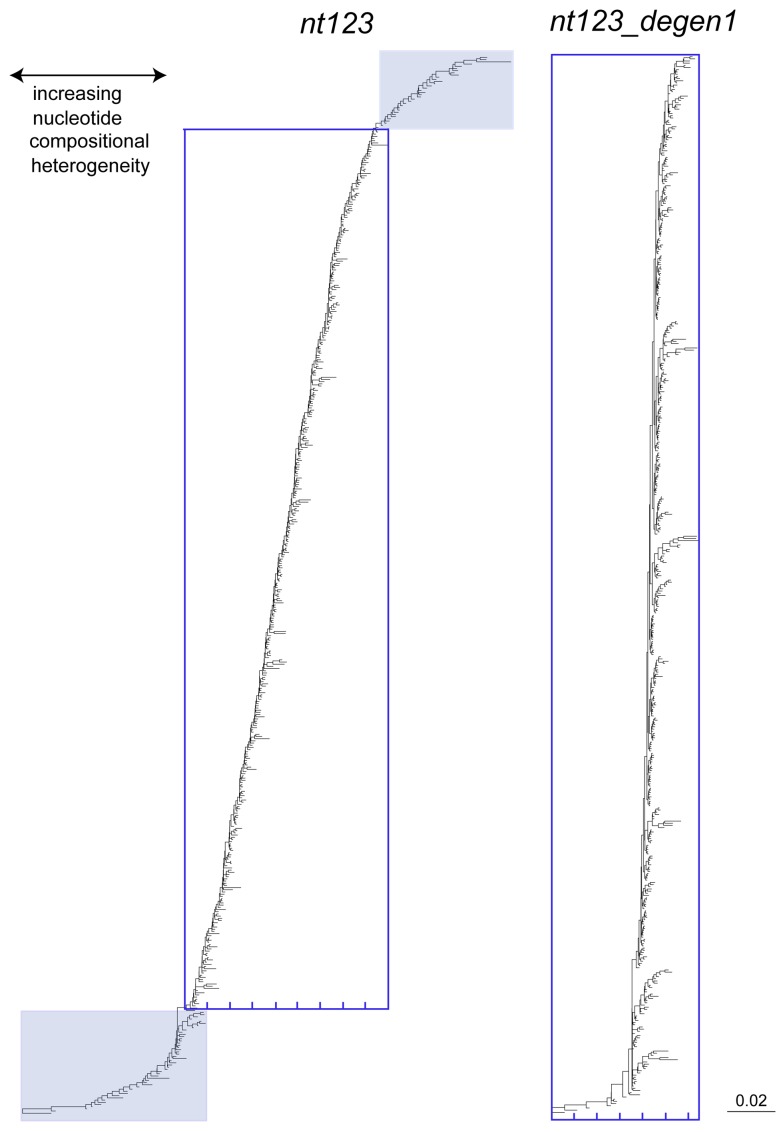
Base-composition distance diagrams derived from analysis of the *nt123* and *nt123_degen1* data sets for 483 taxa. Branching structure obtained by neighbor-join / minimum evolution analysis of Euclidean distances calculated on the proportions of each of the four nucleotide types in each species. All diagrams are drawn to the same scale, and units are 'per cent ÷ 100'. The blue shaded portions identify taxa deleted from *nt 123* data subsets to explore the effect of decreased nucleotide heterogeneity on bootstrap percentages.

While *degen1* strongly supports a paraphyletic Tineoidea (e.g., 87% bootstrap for Tineidae: *Eudarcia* as sister group to all other Ditrysia; Figure 3), *nt123* strongly supports tineoid monophyly (98% for Tineoidea; Figure 3). Despite this strong conflict, both data sets robustly support (i.e., 100% bootstrap) three tineoid subgroups: 'Tineidae − *Eudarcia*', Eriocottidae, and Psychidae. The explanation that we now favor for the observed conflict across these three tineoid subgroups, plus the singleton Tineidae: *Eudarcia*, is that the *nt123* result is biased by compositional heterogeneity, which overall is much greater than that for *degen1* (Figure 4). Our initial approach that led to this conclusion was to identify taxa that caused a difference between the two data sets by systematically deleting one or more of the four taxonomic subgroups (*Eudarcia*; 'Tineidae minus *Eudarcia'*; Eriocottidae; Psychidae), followed by a direct analysis of the compositional features of the problematic subgroups. However, working with the entire data set is computationally impractical, so as an alternative we defined a subset of 63 test taxa that includes almost all tineoid exemplars but a reduced number of non-Ditrysia as outgroups and of non-tineoid Ditrysia (these 63 taxa are identified by three asterisks beside their generic names in Figure S1).

**Figure 5:**
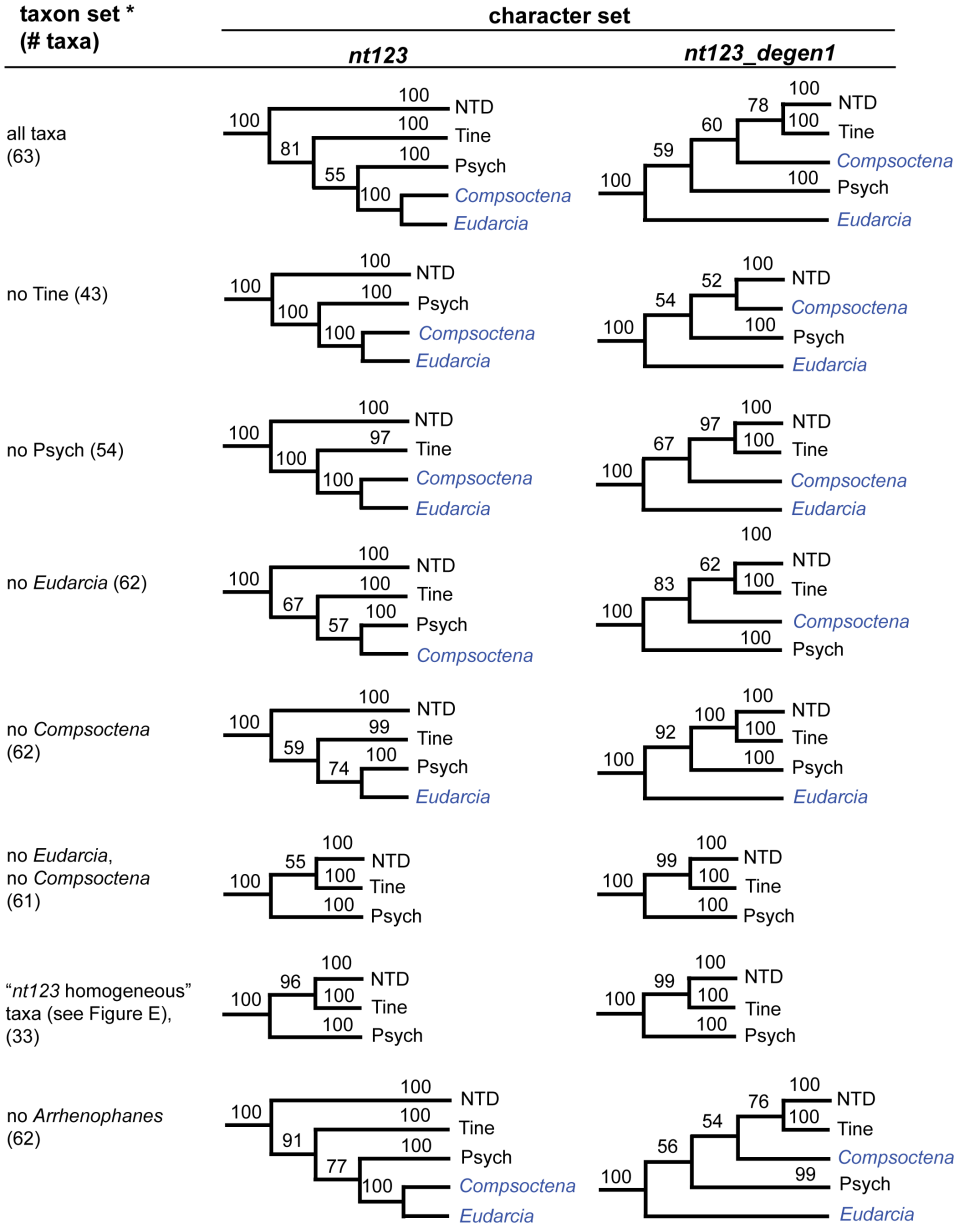
Summary of phylogenetic analyses based on taxon (sub)sampling of Tineoidea. Summary phylogenetic trees are displayed with corresponding bootstrap percentages for analysis of *nt123* and *nt123_degen1* data sets based on different taxon subsamples for Tineoidea. For ease and focus of presentation, only relationships among strongly supported, higher-level groupings are shown (see Figure 3). These groupings are: *Tine*: Tineidae – *Eudarcia* (20 taxa total); *Psych*  =  Psychidae (9 taxa total); *Eudarcia* (currently classified within Tineidae, 1 taxon); *Compsoctena* (currently classified within Eriocottidae; 1 taxon); *NTD*: non-tineoid Ditrysia (27 taxa total); and the non-ditrysian outgroup (not shown), which consist of Palaephatidae + Tischeriidae (5 taxa total).

As a control, analysis of this reduced taxon set results in the same inter-relationships of the four subgroups for *nt123_degen1* and, separately, for *nt123* as the full data sets, although bootstrap values are somewhat altered (cf. Figures 3, 5). For *nt123_degen1*, no matter which subgroup(s) is deleted, the relationships among the remaining subgroups are unchanged relative to the full set of test taxa (Figure 5). However, for *nt123* the Tineoidea become paraphyletic -- and in a manner that matches the *nt123_degen1* result (either altered or unaltered) -- when only two taxa, namely, *Eudarcia* and *Compsoctena* (i.e., the single representative of Eriocottidae in the test set) are deleted, although this paraphyly is not strongly supported, i.e., 55% bootstrap. Removal of either one of these taxa alone greatly reduces *nt123* bootstrap support for tineoid monophyly.

**Figure 6:**
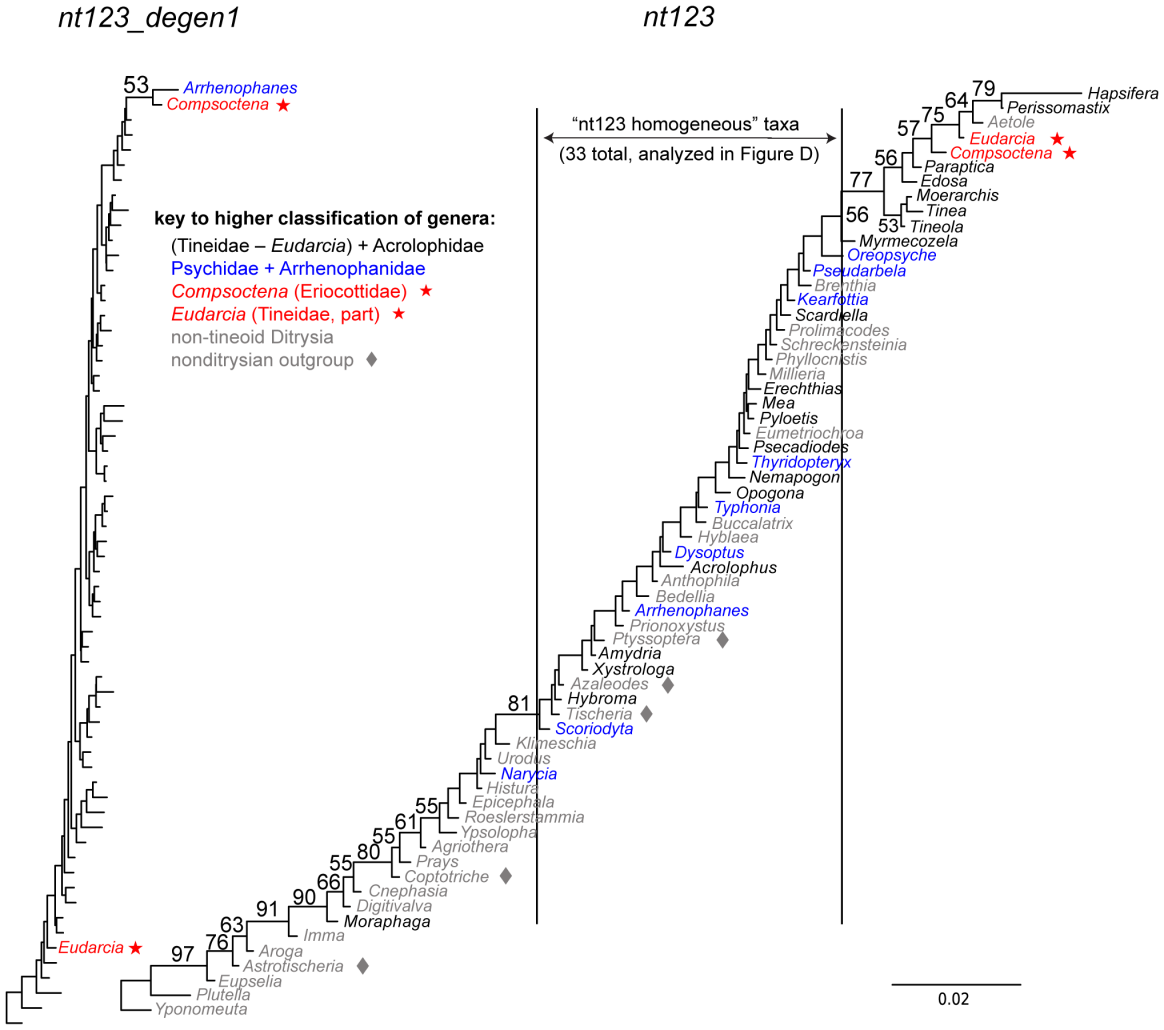
Base-composition distance diagrams of *nt123_degen1* and *nt123* data sets for the 63 taxa in the Tineoidea test set. Both diagrams are drawn to the same scale, and units are 'per cent ÷ 100'. Bootstrap percentages ≥50% are displayed. Bootstrap percentages are based on analysis of total taxon-specific nucleotide compositions, as described in Materials and Methods. All terminal taxa are identified to genus for *nt123* but not for *nt123_degen1*, due to the reduced compositional heterogeneity in the latter data set. The vertical bars identify those taxa used in a phylogenetic analysis (Figure 5) to test the effect of reduced compositional heterogeneity on the analysis of *nt123*. The five sets of taxa whose inter-relationships are analyzed in Figure 5 are color- and/or symbol-coded (see key).

Compositional heterogeneity of the *nt123* data set is more than fivefold greater than that of the *degen1* data set, and it is additionally noteworthy that Tineidae: *Eudarcia* and Eriocottidae: *Compsoctena* are highly and similarly biased in their *nt123* compositions (Figure 6). The 100% bootstrap support for '*Eudarcia* + *Compsoctena'* in the *nt123* analysis is likely due to these compositional features. By contrast, *Eudarcia* and *Compsoctena* do not group in the *degen1* analysis, nor are they highly divergent in composition when synonymous differences are removed (Figure 6).

To test whether a reduction in compositional heterogeneity for *nt123* would lead to a result that more closely approximates the *degen1* result, we deleted 30 taxa at both ends of the *nt123* compositional distance tree in Figure 6 (see vertical boundary lines), resulting in a 33-taxon data set with a >70% reduction in compositional heterogeneity. Maximum-likelihood and bootstrap analysis of the *nt123* and *nt123_degen1* data sets now yield almost identical results, with 96% and 99% bootstrap values, respectively, for a paraphyletic Tineoidea (Figure 5).

## Discussion

### Exploring tree space with large data sets

The current study makes it clear that, when analyzing large data sets, finding the maximum-likelihood topology using a heuristic algorithm, such as that implemented by GARLI, is not a trivial task. This should not be surprising given the enormous number of theoretically possible topologies, plus the fact that many differences in topology yield exceedingly small differences in total lnL values. In the current case, 4608 likelihood search replicates of the complete *nt123_degen1* data set still yield a suboptimal tree, although an improved topology based on further searches differs only in the position of one terminal taxon (see dashed arrow in Figure 2). One might question whether an extended effort to find the best-feasible ML topology is warranted, given the small differences in lnL values among the optimal and the many suboptimal topologies. A positive answer seems warranted, however, since at least some nodes with weak signal are likely to be correctly recovered given a sufficiently thorough search; whereas, it seems counter-intuitive, although not theoretically impossible, that some correct nodes would be lost in overall-improved topologies found with more thorough searches. The recovery of Bombycoidea + Lasiocampidae (BP <50%) and of Gelechioidea (BP 59%), neither of which is present in a strict consensus of the top 10^-2^ % of all *degen1* topologies, are likely examples that illustrate the value of performing multiple search replicates (Figure 2). To further illustrate the importance of performing multiple searches, we calculate from the 483-taxon, *degen1* results shown in Figure 2 that six, 725, and 6903 searches are required to ensure a 95% probability of recovering a topology whose lnL is within 10^-2^ %, 10^-3^ %, and 10^-4^ %, respectively, of the topology of highest likelihood. For the 483-taxon, *nt123* results (not shown), the number of required searches are 3, 70, and 974, respectively.

Unfortunately, there are limits to what is practical for numbers of searches, even with grid computing, particularly given the size of our data sets. Accordingly, for all studies we restricted the number of ML search replicates to 500–1000 for all data sets other than the *nt123_degen1* data set for 483-taxa. Our confidence in any given node must, therefore, be tempered by this practicality. Indeed, it is an interesting further observation that not all nodes are recovered at the same frequency. Notable for this report, most of the nodes along the backbone are frequently not present in strict consensuses of the top 10^-3^ % of all topologies (Figure 2), indicating that the very nodes of interest are ones that are particularly difficult to recover. Fortunately, these backbone nodes are all present in the top 10^-4^ %. There is also a general tendency for hard-to-recover nodes to have lower bootstrap values, but there are exceptions, e.g., the Pyraloidea (bootstrap, 74%) is not present in the strict consensus of the top 10^-3^ % of all topologies (Figure 2).

In principle, what applies to the ML search could also apply to the search of each and every bootstrapped data set in order to calculate an accurate bootstrap value, making accurate bootstrap analysis a truly daunting task. However, before considering this there is an additional complexity in that the bootstrap provides a statistical summary measure of results from multiple pseudoreplicated data sets. The variance of the bootstrap percentage decreases as the number of replicates increases, but it decreases more rapidly for higher bootstrap percentages than lower ones. Following a standard model [26], we chose to perform approximately 500 bootstrap pseudoreplicates for each analysis. This number ensures, within the assumptions of the model, that bootstrap percentages in the general range of 60% and higher are accurate to within 5%.

We have empirically tested the effect of increasing numbers of search replicates on the resulting bootstrap values (Tables 1, 2). For analysis of the *nt123_degen1* and *nt123* data sets, there are 15 and 22 higher-level nodes, respectively, whose bootstrap values increase from 1 to 5 search replicates, of which 3 and 6, respectively, increase further from 5 to 10 search replicates. None increase by more than 5% points beyond 10 search replicates, and all have final bootstrap values that are ≥55%, assuring that the standard error should be in the range of 5% or less. (No conclusions are made for values <50%.) It is on this empirical basis that the standard condition of 15 search replicates per bootstrap pseudoreplicate was selected for other analyses. Interestingly, Pyraloidea is one of the nodes whose bootstrap value is sensitive to number of search replicates, paralleling a similar difficulty in its recovery for ML searches (Figure 2). However, for Pyraloidea many fewer replicates are needed to achieve an accurate bootstrap value than to recover this group in the ML topology. This seeming paradox could reflect the particular characteristics of each somewhat-distinct bootstrap data set, but of course recovering a particular node in an ML topology and accurately (enough) estimating its bootstrap value are not directly equivalent undertakings either.

The just-mentioned results stimulated us to reinvestigate the matter of number of search replicates needed to generate accurate bootstrap percentages for GARLI and the given parameters. To do this, we increased the number of search replicates to 1000 for each of 505 bootstrap pseudoreplicates of the 483-taxon, 19-gene *nt123_degen1* data set, and compared the resulting bootstrap values with those derived from 15 search replicates (Table 3). In light of our ML search results, it would have been desirable to increase the number of search replicates to ≥ 7000, but this simply was not practical. Even given our access to considerable computational resources, performing this one analysis with 1000 search replicates was at the limits of feasibility, as it consumed approximately 3-million computer-processor hours ( =  3.4 centuries). The results are modestly surprising and add further complexity in interpretation to an already complex study. The eight nodes that show changes (all increases) in bootstrap values of >10% provide clear evidence of the inadequacy of relying on 15 search replicates, although of course all of these should thereby be interpreted as introducing *underconfidence* in our results, not overconfidence. Not surprisingly given the ML results, when each of the 1000 topologies generated for each of the 505 bootstrap pseudoreplicates is examined, it turns out that in 504 of the bootstrap pseudoreplicates the best topology is recovered only once, so even with 1000 search replicates per bootstrap pseudoreplicate we cannot be confident that the enhanced bootstrap percentages are accurate (results not shown). The difficulty can be explained from two perspectives. From the perspective of model choice, the estimate that bootstrap values in the range of 60% and above would have no more than 5% points variation at the 95% confidence level assumes a binomial distribution for the proportion of bootstrapped trees containing a particular group. Seemingly, this assumption is incorrect for some groups. From the perspective of the individual groups themselves, some are simply harder to recover than others; that is, their recovery requires more search replicates. Of the five groups with bootstrap values >65% after 15 search replicates, two (Sesiidae, Cossidae: Metarbelinae) are "difficult to recover" in the ML search (Figure 2); that is, they are not present in all of the top 10^-2^ % of all 4608 topologies recovered. The other three are not notably difficult to recover in the ML analysis, at least for this data set.

The effect of search effort on bootstrap values has been little studied [27]–[29]. The challenge of getting accurate bootstrap values probably relates to the number of taxa analyzed, since tree space itself increases exponentially with number of taxa, as does the computational effort required. By modern standards the current study is no longer "large", so this problem may be even more challenging for studies larger than ours. Finally, this study provides only a single datum -- out of practical necessity -- and it raises new questions. What changes would have been observed if we could have applied increased numbers of search replicates to our other analyses? What changes to the user-controlled parameters of the GARLI program might improve the efficiency of the search? How would our findings in GARLI relate to those derived from other ML and bootstrap search algorithms? These are important issues for future studies.

### Selecting characters for higher-level phylogenetic analysis

In the preceding section we discussed ways to improve heuristic search results through more thorough searches of tree space. In this section we discuss the relative contributions of two categories of nucleotide change, namely, synonymous and nonsynonymous, and their implications for improved phylogenetic analysis. We conclude that both synonymous and nonsynonymous change provide valuable phylogenetic signal across Lepidoptera, but that these signals are optimally informative at different phylogenetic levels. This is generally the case because synonymous change occurs more rapidly, and is particularly useful for resolving more recent divergences that receive little support from the more slowly evolving nonsynonymous change. By contrast, nonsynonymous change is less subject to the multiple-hits problem at deeper levels in the tree, where it is particularly useful. Equally important for this study, however, is that nonsynonymous signal is less affected by compositional heterogeneity at all levels (Figure 4). Such heterogeneity can introduce an analytical bias that distorts the phylogenetic signal of primary sequence evolution, and can even result in strong support for incorrect nodes [24].

These general observations about synonymous and nonsynonymous change have been widely acknowledged, and multiple approaches have been implemented to obviate their consequent problems for phylogenetic analysis. One standard approach has been to apply separate "partition" models to nonsynonymous and synonymous change (either as implemented in this report or, less discriminatingly, by codon position). While this approach can be effective (e.g., see [30] and references therein], we found little difference between the partitioned and unpartitioned analyses in this study (Figure 3).

A second common approach is to delete all third-codon-position characters, which eliminates synonymous (and nonsynonymous) change at third codon positions, while still allowing synonymous change at first codon positions. However, even this reduced level of synonymous change causes problems for deep-level arthropod phylogeny [22]-[24]. Therefore, in this and other studies we have instead "degenerated" all nucleotides (the *degen1* approach) such that synonymous change should be largely eliminated but without any loss of information from nonsynonymous change [23]–[25].

Previous studies of Lepidoptera using some or all of the same genes as in the current study have demonstrated the utility, and indeed the necessity, of a nonsynonymous-only approach for robustly resolving a novel group at the base of Ditrysia ('Apoditrysia + Gelechioidea'; [6]; also observed by Mutanen et al. [5]). Conversely, other studies directed at relationships within superfamilies -- e.g., Bombycoidea [8], Gracillarioidea [9], Tortricoidea [11], and Pyraloidea [10] -- have illustrated that total synonymous + nonsynonymous change provides much more overall support than nonsynonymous alone. Of course, data sets that include synonymous change are more prone to signal distortion from compositional heterogeneity, so this must also be considered. From these studies, we conclude that no single approach is warranted across the entire Lepidoptera, and it is for that reason that we have performed both total *nt123* and *degen1* analyses, along with independent tests of compositional heterogeneity. Another reason is that "deep" and "shallow" nodes are relative terms that cannot yet be applied across lepidopteran phylogeny, since neither a robust phylogeny nor a robust dating of internal nodes is available.

A direct comparison of higher-level node support for the 483 taxa provided by analysis of the *nt123* and *nt123_degen1* data sets can be made from Figure 3. Excluding the Tineoidea (discussed in the next section), there are 16 nodes above the family level with bootstrap values that differ by at least 10%. Nine are more strongly supported by *nt123* than by *degen1*: 'Bombycoidea + Lasiocampidae'; 'Sematuridae + Epicopeiidae'; 'Geometridae + Uraniidae'; Pyraloidea; Macroheterocera (*sensu* van Nieukerken [1]); 'Mimallonidae + Doidae'; 'Zygaenoidea − (Zygaenidae + Lacturidae)'; 'Adeloidea + Andesianidae'; and 'Exoporia + Lophocoronidae'. Seven nodes receive greater support from *degen1* than from *nt123*: 'Nymphalidae + Lycaenidae'; 'Papilionoidea − (Papilionidae, Hesperiidae)'; 'Papilionoidea − Papilionidae', Gelechioidea, 'Dalceridae + Lacturidae', 'Hepialidae + Palaeosetidae', and 'Adelidae + Heliozelidae'. All 16 nodes are recovered in the *degen1* ML topology, while the *nt123* ML topology includes all except two: 'Papilionoidea − (Papilionidae, Hesperiidae)' and 'Adelidae + Heliozelidae'. Such a high level of agreement across data sets argues against the influence of a strongly distorting signal based on compositional heterogeneity and for the phylogenetic accuracy of the nodes, particularly for those 10 that receive strong bootstrap support from one or the other data set. However, this conclusion must be tempered by the fact that those seven nodes with lower support from *nt123* than *degen1* (e.g., 'Papilionoidea − Papilionidae') must contain some enhanced conflicting signal in the synonymous portion, since *nt123* captures both synonymous and nonsynonymous signals, while *degen1* captures only nonsynonymous.

### Compositional heterogeneity and resolution of the Tineoidea

Despite the many similarities between the *nt123* and *degen1* ML topologies, there are also numerous differences (Figure 3). However, with one exception these differences are weakly supported by one or the other or both data sets. As such, the source and evaluation of these disagreements are less certain. The one exception occurs in the case of Tineoidea, which *nt123* strongly supports as monophyletic but *degen1* strongly supports as paraphyletic (Figure 3). A series of taxon-deletion experiments (Figure 5), coupled with an assessment of compositional heterogeneity (Figure 6), strongly supports the hypothesis that the difference results from a major distorting effect of nucleotide composition on the synonymous signal of selected taxa. Most convincingly, when a subset of compositionally more homogeneous taxa is analyzed, both *nt123* and *degen1* strongly support tineoid paraphyly (Figure 5).

So, it would seem that Tineoidea decisively illustrate the challenge heterogeneous compositions present for generating phylogenetically accurate *nt123* results. It also seems reasonable that the many cases in which *degen1* provides significantly greater support for a particular node than *nt123* (see preceding section) also reflect underlying distortion and/or conflict in the synonymous signal. We suggest that *degen1* results provide a valuable check on those of *nt123*, particularly in those cases when *nt123* support is high and *degen1* favors a conflicting grouping. However, we are not suggesting that *degen1* results are infallible, only that they are less prone to error caused by compositional heterogeneity. When bootstrap values are low in *degen1* analyses, the stochastic nature of evolutionary change still makes accurate node assignment problematic.

### Selecting taxa for higher-level phylogenetic analysis

While it seems like a straightforward proposition that taxa should be selected to represent known and hypothesized larger groups, not all taxa serve as equally good representatives. Some taxa may be especially fast evolving, and be long-branch attractors of distant taxa. Others may evolve in such a manner that the nucleotide composition of their gene markers becomes more similar to that of distantly related taxa than to more closely related ones, as demonstrated in this study for selected tineoids (discussed in previous section; Figures D, E).

One way to explore phylogenetic results beyond total-data analysis is to test the sensitivity of a result (in our case, a bootstrap percentage) to the removal of these potentially problematic taxa. But exactly which taxa ought to be removed, even assuming their inclusion as a representative of a group is not obligatory? In the remainder of this section, we discuss phylogenetic results based on three general approaches to taxon subsampling, sometimes performed in combination: 1) removal of "rogue" taxa based on the *RNR* and *Adams-consensus* approaches (described in Materials and Methods), 2) removal of clusters of taxa (in addition to Tineoidea, whose subsampling has already been discussed) that are compositional outliers relative to the mean composition of all taxa, and 3) removal of distant outgroups. Many of these tests have been performed separately for *nt123* and *degen1*.

A general concern with all taxon deletion studies is that resulting changes in topology or node support may or may not actually be due to the hypothesized problematic feature of the deleted taxon itself. For example, a taxon that happens to be long branch or compositionally heterogeneous might be *required* in order to maintain the integrity of a monophyletic group, even though it would do this more effectively if it were not long branch or compositionally heterogeneous. Thus, it is worth emphasizing the exploratory nature of these subsampling studies. As concerns the first approach (i.e., rogue identification and removal), rogue taxa by definition are not robust to various analytical perturbations. In this regard, it is worth emphasizing that long-branch taxa can be either stably or unstably positioned -- correctly or incorrectly -- and, thus, are not necessarily rogue taxa as such. As concerns the second approach (identification and removal of taxa with shared unusual compositions), its utility has already been demonstrated for Tineoidea. In other taxa for which compositional divergence is not so striking, the effect is more difficult to separate from other contributors to the total signal. It is also worth noting that taxa with compositions that are unusually divergent from the mean composition are not necessarily rogue taxa either. A strong compositional atypicality (relative to the mean) could by itself result in increased bootstrap support, and this support might either be consistent with phylogeny (for clusters of related taxa) or not (for clusters of unrelated taxa). As concerns the third approach (removal of outgroup taxa), this would seem to present the fewest challenges to accepting altered results, because ingroup taxa are not deleted. Its potential utility is based on the premise that there exist taxa in the outgroup that affect the position of taxa in the ingroup, e.g., through their shared and unusually biased compositions. Of course this assumes that outgroups are indeed outgroups and that the basal-most subgroup relationships within the ingroup -- the ones most likely to be affected by altering outgroup taxa -- either are not altered or are of lesser interest.

The effects of taxon sampling on seventy-two higher-level groups (some conflicting) were assessed in 15 tests (Tables S1, S2) that collectively show many notable differences from the 483-taxon *degen1* or *nt123* result. In general, removal of rogue taxa either increases bootstrap values or has little effect, but does not decrease them. Of the most notable *degen1* results (Table 4), there are five nodes (Macroheterocera; 'Epermeniidae + Copromorphoidea in part'; 'Schreckensteiniidae + Douglasiidae'; Gelechioidea; and 'Acanthopteroctetidae + Neopseustidae') that show increases in bootstrap percentages of ≥40% points, always to final values of 70 - 80% when the *RNR rogues* are removed, cf. columns 3 and 5. As an aside, we also note that the single taxon which remains suboptimally positioned (*Copromorpha*) after 4608 search replicates of the 483-taxon *nt123_degen1* data set (Figure 2) is also a rogue taxon (Text S1).

There is another very striking increase in *degen1* node support (from 72% to 92% bootstrap) -- for 'Exoporia + Lophocoronidae' -- when two additional, neighboring taxa (i.e, Acanthopteroctetidae: *Acanthopteroctetes* and Neopseustidae: *Neopseustis*) are removed (see column 6 in Table 4). We note that these two taxa match the criteria for rogue taxa according to the *Adams-consensus* approach, although in this report this approach was applied only to taxa within Apoditrysia, so this increase too could be considered a "rogue" effect.

The effect of removing distant outgroups (see columns 7, 9 in Table 4) is somewhat difficult to evaluate for *degen1* because many of the relevant nodes are not strongly supported by any approach. The largest effects are for Macroheterocera (bootstrap increases from 39% to 60%,) and Gelechioidea (59% to 68%) when the analysis is restricted to Apoditrysia. Regardless, it is clear that the effect of removing rogue taxa is much greater than simply removing distant outgroups.

Of the most notable *nt123* results (Table 5), nine groups ('Bombycoidea + Lasiocampidae'; Macroheterocera; 'Mimallonidae + *Doa'*; 'Callidulidae + Hyblaeidae'; ‘Callidulidae + Hyblaeidae + Thyrididae'; 'Tortricoidea + Immoidea'; 'Acanthopteroctetidae + Neopseustidae + Eriocraniidae', 'Exoporia + Lophocoronidae'; and 'Glossata − (Exoporia + Lophocoronidae)') show increases in bootstrap support of >10% points, always to final values ≥70%, when the *RNR rogues* are removed (cf. columns 3 and 5). There are no examples of bootstrap decreases with rogue removal for groups that have bootstrap values of at least 50% in the full data set. The additional removal of 76 heterogeneous taxa (plus 6 more already in *RNR rogue* set) can have large effects, and these are not unidirectional. The largest effects are: 20% point increase for 'Tortricoidea + Immoidea', 18% point decrease for 'Acanthopteroctetidae + Neopseustidae + Eriocraniidae', and 39% point decrease for Glossata minus Exoporia + Lophocoronidae. Straightforwardly interpreted, these results provide additional strong support for 'Tortricoidea + Immoidea', but reduced confidence for the other two groupings.

The effect of rogue removal under the *Adams-consensus* approach is particularly dramatic (i.e., 94% point increase) for node 46 ('Zygaenoidea + Cossoidea + Sesioidea') but much less so under the *RNR approach* (Table 5). This appears to be due to the absence of taxa belonging to Zygaenoidea: Cyclotornidae and Zygaenoidea: Epipyropidae from the former analysis. Overall, a comparison of the two approaches to rogue identification shows that the *RNR approach* yields significantly higher bootstrap values than the *AC approach* five times, the *AC approach* yields higher values than the *RNR approach* three times, while there is no significant difference two times.

Removal of distant outgroups (columns 7, 9) is clearly beneficial in some cases, although, as for *degen1*, rogue removal yields higher support values more consistently.

So, what can we conclude about the various approaches that rely on deleting selected taxa? Firstly, it is clear that removal of rogue taxa oftentimes increases bootstrap support and seldom, if ever, decreases it. This is an encouraging observation. Secondly, removal of distant outgroups can be useful, but it is not as effective as, and probably is not necessary in addition to, rogue taxon removal. Thirdly, removal of heterogeneous taxa prior to analysis of *nt123* data sets, when coupled with removal of rogue taxa, can increase, decrease, or leave unchanged bootstrap support relative to removal of rogue taxa alone, as one would expect if some of the nodes were accurate and others inaccurate. This point has already been clearly demonstrated for Tineoidea (Figure 5), but there are a few other instances just discussed and apparent from an inspection of Table 5.

### Higher-level phylogeny of the Lepidoptera

In this section we review current understanding of major features of the “backbone” lepidopteran phylogeny -- relationships among superfamilies -- in light of this and other recent molecular studies. The discussion below refers primarily to Figure 3, which shows the *degen1* topology condensed to superfamilies or the largest monophyletic fragments thereof. Our analyses also yield much new information about the monophyly of and basal divergences within superfamilies and families. However, we defer most discussion of such relationships to an ongoing series of studies on individual superfamilies or groups thereof in which the taxon sample is expanded beyond that included here (e.g., [8]–[11]; see Materials and Methods section on taxon sampling below).

As is evident in Figure 3, molecular data abundantly confirm the existence of a highly asymmetrical topology at the base of lepidopteran phylogeny, first noted by Hennig ([31] *fide* Kristensen [32]) and corroborated by subsequent morphological studies [32]. There is now very strong molecular support for a majority of those early major divergences. Among the non-ditrysian lineages, six of the eight “backbone” nodes (nodes 2–9 in Figure 3), including the previously-recognized major clades Glossata, Heteroneura and Eulepidoptera (Figure 1), have bootstrap support of 95% or greater in one or more analyses, as does the clade Exoporia (node 28). There is also strong molecular support for several novel proposals, such as apparent non-monophyly of Palaephatidae (node 9) and the grouping of Lophocoronidae with Exoporia (node 27, see also Table S1), despite morphological evidence to the contrary. Some relationships, however, remain very weakly supported, for example at the base of Glossata (nodes 4 and 5), and there is striking lack of confirmation for some clades included in the working hypothesis of Figure 1A, such as Myoglossata, Neolepidoptera, and Lepidoptera excluding Micropterigidae. A detailed update on phylogeny and classification among the non-ditrysians will be provided in a separate, forthcoming publication.

Support is also strong for early divergences within the Ditrysia (Figure 3, nodes 10–15). As argued above, the oldest lineages belong to the Tineoidea as previously defined, which now appear to be paraphyletic. Paraphyly for Tineoidea was also seen in the analysis of Mutanen et al. [5]. Support for this conclusion is further strengthened by the 1000 search replicate per bootstrap pseudoreplicate analysis of *degen1* (Table 3). We will update the phylogeny and classification of groups currently placed in Tineoidea in a forthcoming publication that will propose a new family for *Eudarcia* and relatives.

Our results provide very strong evidence that all non-tineoid ditrysians form a monophyletic group (node 14; BP  = 100, all analyses) that divides basally into Yponomeutoidea + Gracillarioidea (BP ≥97%, all analyses) versus all others (node 15; BP ≥97%, all analyses). The latter corresponds to Apoditrysia sensu Minet [33], [34] expanded [1] to include Gelechioidea. A relationship between gelechioids and Apoditrysia had been deemed plausible by Kristensen and Skalski [35] based on putative synapomorphies in male genital structures [36], proboscis morphology [37] and larval setal pattern.

In dramatic contrast to those in earlier-originating clades, “backbone” relationships in the Apoditrysia *sensu lato* largely lack strong support. Of the approximately 27 nodes within Apoditrysia *sensu lato* in Figure 3 which subtend two or more superfamilies (no classification fully matches our findings on superfamily definitions), all but three (Macroheterocera, 'Mimallonidae + *Doa*', 'Bombycoidea + Lasiocampidae') have bootstrap supports <50% in all analyses of the full 483-taxon data set; only one has bootstrap support as high as 71% ('Mimallonidae + Doidae'). Moreover, the majority of these 27 nodes do not even occur in the best trees from other analyses (Figure 3). Two additional "backbone" nodes attain bootstrap support >50% with more thorough bootstrap searches, namely, 'Macroheterocera + Pyraloidea + Hyblaeidae' (BP, 71%) and 'Apoditrysia − Urodidae' (BP, 57%; Table 3). Similarly challenging results are reported in all previous molecular studies of relationships in Apoditrysia [4]–[6], which appear to represent an exceptionally difficult phylogenetic problem.

Strong, node-by-node resolution of relationships among apoditrysian superfamilies thus appears mostly beyond the reach of even this largest-ever data set. As detailed below, however, closer inspection shows on two grounds that substantial progress toward that goal has nonetheless been made. First, on a broad scale, the *degen1* topology in Figure 3 shows much greater than random similarity to the morphology-based working hypothesis (Figure 1A), as well as close similarity to the results of our own (much smaller) previous studies (Figure 1B) and those of others (Figure 1C, [5]). Second, our experiments, after removal of “rogue” taxa and other forms of taxon subsampling, point to the existence of stronger signal for a number of putative clades in Apoditrysia than is evident in Figure 3 (Tables 4, 5, S1, S2; discussed below).

The “lower” (i.e., non-obtectomeran) Apoditrysia have been so problematic that the morphology-based working hypothesis (Figure 1A) postulates only one tentative grouping in this tree region, Cossoidea + Sesioidea + Zygaenoidea (sensu Kristensen [7]). This grouping is recovered entirely in our *degen1* analysis (Figure 3), albeit with very low support. It is also recovered or nearly recovered, albeit with very low support, in all other analyses in this study (e.g. nt123; Figure S2) and in other recent reports [4]–[6]. In the current study, bootstrap support for Cossoidea/Sesioidea/Zygaenoidea is almost always increased in analyses of both *nt123* and *degen1* from which rogue taxa have been deleted (Tables 4, 5), rising to 96% for *nt123* with apoditrysian “AC rogues” removed. The 28 rogues (Text S1) include 10 of our 57 exemplars from Cossoidea/Sesioidea/Zygaenoidea, of which five represent the two problematic parasitic families of Zygaenoidea, Cyclotornidae and Epipyropidae. Thus, the 96% bootstrap value does not apply to the entire hypothesized clade as sampled here. Nonetheless, the dramatic increase in support, coupled with consistent recovery or near recovery of the clade in analyses of the full data set, suggests that strong underlying signal for Cossoidea + Sesioidea + Zygaenoidea is both present and obscured by the inclusion of unstably placed taxa.

One of the striking points of approximate agreement between our findings and the largely morphological working hypothesis is the complete recovery of Obtectomera [34] in the slightly modified sense of van Nieukerken et al. [1] by our most conservative data set (*degen1*; Figure 3; node 20), albeit with very low support (BP = 6%). Very similar groupings, though always poorly supported, are also found in our other present analyses (Figure S2), as well other recent studies, provided that synonymous change is in some way down-weighted [4]–[6]. In this study, bootstrap support for Obtectomera under *degen1* rises from 6% to 40% when the 47 rogue taxa identified by *RNR* (see Materials and Methods) are removed (Table 4), suggesting that unstably-placed taxa are indeed part of the reason for low support. The 33 apoditrysians among the 47 *RNR* rogues (Text S1) consist disproportionately of exemplars that are the sole representatives of their small, monobasic superfamilies (Table S1). Such taxa make up only 3% (10/344) of the Apoditrysia sampled, but constitute 27% (9/33) of the rogues. Thus, one obstacle to clear resolution of major groups, in a mega-diverse clade such as Apoditrysia, may be the difficulty of placing the many small, taxonomically isolated families that such clades typically include.

In our *degen1* analysis (Figure 3), the sister group to Obtectomera is Gelechioidea (node 19). Bootstrap support is very weak (14%), but rises with all forms of rogue taxon deletion (Table 4), to as high as 65%, suggesting again underlying signal obscured by unstably placed taxa. This grouping, or something like it (i.e., with inclusion of one or two small additional superfamilies), is found in all previous analyses in which synonymous change is partially to completely excluded [4]–[6]; however, it is not supported by *nt123* (Figure S2). It nevertheless seems likely that Gelechioidea are closely related to Obtectomera.

Within Obtectomera, there is now considerable molecular support for monophyly of Macroheterocera sensu van Nieukerken et al. [1], with the addition of Mimallonidae. Macroheterocera in this modified sense consists of Macrolepidoptera sensu Kristensen [7] minus the expanded concept of the butterflies (Papilionoidea sensu van Nieukerken et al. [1]). This group was recovered by the ML analysis of Mutanen et al. [5], and by some of the analyses of Regier et al. [4] and Cho et al. [6], without strong support. In the present study, it is recovered in all analyses of the full data set. Although the maximum bootstrap for the full data set is 64% (*nt123_partitioned*; Figure 3), support increases markedly with rogue deletion and other forms of taxon sub-sampling, to as high as 89% (Tables 4, 5), again suggesting strong underlying signal obscured by unstably placed taxa.

In all recent molecular studies [4]–[6], there has been consistent support for Pyraloidea, with or without the addition of one or two other small superfamilies, as nearest relatives to the Macroheterocera, though always with weak support. In the present study, the ML trees for all analyses of the full data set unite Pyraloidea alone with Macroheterocera, but with weak support. Support increases somewhat with rogue deletion/taxon-subsampling, to a high of 68% under *nt123* (Tables 5, S2). Under *degen1*, the alternative grouping of Pyraloidea + Hyblaeidae with Macroheterocera, though not found in the ML tree, has higher bootstrap support, reaching 71% when search replication per bootstrap pseudoreplicate is raised to 1000 (Table 3). This grouping also occurs in the ML tree reported by Mutanen et al. [5]. It seems clear both that Pyraloidea are closely related to Macroheterocera, and that their proximity to Hyblaeidae remains possible but still problematic, as reported previously in our expanded study of Pyraloidea [10]. Sequencing of the enigmatic African genus *Prodidactis*, whose larvae, but not adults, are pyraloid-like [38], might help to resolve this problem.

Within Macroheterocera, as at the base of Apoditrysia, relationships among superfamilies remain largely unresolved, with a few possible exceptions. Lasiocampoidea are united with Bombycoidea in all of our analyses (Figure 3), with bootstrap support rising from ≤ 63% to as high as 97% (*nt123*; Table 5) following rogue deletion. This long-accepted pairing [34], [39] was strongly supported by the results of Cho et al. [6], and is also supported by morphological synapomorphies [40]. It seems likely to be real.

A second pairing supported by all of the present analyses is that of Mimallonoidea + Doidae (Figure 3). Bootstrap support under *nt123* rises from 71% with the full taxon set to 85–92% following rogue deletion / taxon subsampling. Despite these encouraging molecular indicators, there are grounds for doubt: the grouping has no known morphological support, and did not emerge in previous molecular studies with smaller data sets. It contradicts the proposal by van Nieukerken et al. [1] of a superfamily Drepanoidea consisting of just Drepanidae, Cimeliidae and Doidae, but reinforces the recent separation of Doidae from Noctuoidea, with which it has never grouped in any molecular analysis despite sharing two seemingly strong morphological synapomorphies with that superfamily [41].

Finally, all of our analyses reinforce the previously reported grouping of 'Sematuridae + Epicopeiidae' ([4], [6]; Figure 3), formerly placed in different superfamilies [7]. Bootstrap support from *nt123* is 91%. Although support is weak under *degen1* (but not *nt123*), these families group in turn with the strongly-supported pair Geometridae + Uraniidae (Figure 3; 91% bootstrap for *nt123*), yielding Geometroidea sensu van Nieukerken et al. [1]. Geometroidea in this sense are also monophyletic, albeit without strong support, in all of our previous analyses [4], [6]. This definition of Geometroidea is thus a reasonable working hypothesis.

### Conclusions and prospectus on lepidopteran phylogeny

The past decade has seen tremendous advances in our understanding of lepidopteran phylogeny at all levels, providing a radically improved phylogenetic framework for the study of lepidopteran biology and evolution. Molecular data have proven especially powerful for defining superfamilies and relationships within them, as exemplified by the bootstrap support at those levels seen in Figure 3. In a remarkable burst of community progress, robust molecular phylogenies for nearly all of the major superfamilies (those containing hundreds to thousands of species), combined with review of the morphological evidence, have been published in the past few years or will be forthcoming shortly. Recently appearing examples (not an exhaustive list) include studies of Bombycoidea [6], Gelechioidea [15], Geometroidea [5], [42], [43], Gracillarioidea [9], Noctuoidea [12], [13], [44], Papilionoidea [45], Pyraloidea [10], Tortricoidea [11] and Yponomeutoidea [46]. In all of these superfamilies, a majority of the major divergences (at least) now seem credibly established, though important uncertainties remain. Progress is now rapid also at more subordinate levels.

Above the superfamily level, progress has been greatest at the highly asymmetrical base of lepidopteran phylogeny, as is evident in Figure 3. A majority of the earliest divergences, giving rise to the non-ditrysian lineages, are now strongly established by both morphology and molecules, although a number of important problems remain. Molecular data also strongly resolve the earliest divergences in the Ditrysia, giving rise to successive lineages in the paraphyletic Tineoidea sensu lato followed by the split between 'Yponomeutoidea + Gracillarioidea' and its sister group Apoditrysia (now expanded to include Gelechioidea). These are more recent proposals, and morphological evidence bearing on them has yet to be fully evaluated.

The hardest remaining problem is achieving a fully and robustly resolved “backbone” phylogeny linking the superfamilies of Apoditrysia. Though they have left many questions unanswered, analyses of the data sets so far have yielded substantial progress. Few if any nodes subtending two or more apoditrysian superfamilies are definitively established (Figure 3). However, if a number of small superfamilies and aberrant members of larger ones are set aside as “rogue” taxa, there is now strong molecular evidence for a group approximating the Macroheterocera (macro moths) of van Nieukerken et al [1]; moderately strong support for Pyraloidea as sister group to these; and weaker but credible evidence for a still broader group approximating the Obtectomera of Minet [47], to which the Gelechioidea now appear closely related. Among the “lower” (non-obtectomeran) Apoditrysia, rogue taxon removal also yields strong evidence for the long-standing hypothesis of monophyly for a group consisting of most if not all Cossoidea, Sesioidea and Zygaenoidea.

On a broad scale, then, despite some exceptions, the molecular evidence largely supports the morphology-based working hypothesis (Figure 1A; [7]) and the major ecological/ evolutionary trends it has suggested. These include, among others, a dramatic increase (though with rampant parallelism and reversal) in mean body size since the early ancestors of Lepidoptera; non-ditrysian moths, and ditrysians outside Macroheterocera (along with butterflies {Papilionoidea}), are sometimes referred to as Microlepidoptera. Paralleling the increase in size is an overall trend from the internal feeding (endophytophagy) typical of non-ditrysians (though not Micropterigidae), to concealed external feeding (leaf rolling, leaf tying and the like), widespread in non-obtectomeran ditrysians, to the exposed external phytophagy typical of most families of Macroheterocera and of butterflies [48]. Thirdly, a majority of the families of the Macroheterocera, as well as their apparent sister group Pyraloidea, typically bear bilateral ultra-sound detecting tympanic organs on the thorax or abdomen, thought to function most often for averting predation by bats that hunt using sonar. Such “ears” may or may not be homologous within 'Macroheterocera + Pyraloidea', but they occur only sporadically elsewhere in Lepidoptera [49]–[51].

While establishment of broad life history trends and the approximate phylogenetic groupings that underlie them is a major step forward, a full understanding of lepidopteran evolution, including quantitative assessment of the evolutionary frequency, causes and consequences of the traits involved, will require a more robust and detailed resolution of relationships among the apoditrysian superfamilies. It is possible that continuing analyses of this and other existing data sets, by gene-tree/species-tree and other methods, will yield at least some additional signal. We think it most probable, however, that greatly increased amounts of data, and/or new kinds of characters, will be required to attain fully robust resolution among the Apoditrysia, including its “rogue” members. To help test this hypothesis, we are currently collecting RNA-seq transcriptome data for phylogenomic re-analysis of the apoditrysian families, on the model of Hittinger et al. [52].

Finally, a complete understanding of lepidopteran evolution will require, in addition to a robust branching structure, a rigorous estimate of the geological time scales over which these divergences have occurred. The use of fossil-calibrated molecular dating is less advanced in Lepidoptera than in other insect groups, mainly because the fossil record in this order is relatively sparse and poorly studied [53], [54]. Very few lepidopteran fossils have rigorously established, synapomorphy-based identifications, and as yet, no molecular dating for any lepidopteran group has been explicitly based on synapomorphy-grounded calibration points. Building on our recent comprehensive review of the lepidopteran fossil record [55], we are preparing an estimate of lepidopteran divergence times using the data set reported here in conjunction with synapomorphy-based fossil calibrations.

## Materials and Methods

### Taxon sampling and identification, template preparation

The data for this study were generated as part of a larger effort -- the ‘Leptree’ project (www.Leptree.net) -- aimed at producing both a “backbone” estimate of relationships among the 47 superfamilies of Lepidoptera and separate estimates of deeper relationships within each major superfamily and family. In all, about 900 species were sequenced, representing all the lepidopteran superfamilies, families and subfamilies for which we were able to obtain material suitable for sequencing. Nearly all of the approximately 900 species were sequenced for five genes (6.6 kb) shown previously to provide generally strong resolution within superfamilies [4], [17]. Pilot studies also showed, however, that this gene sample would probably not provide a robust estimate of relationships among superfamilies [4]. To increase resolving power for the “backbone” phylogeny, as well as for more recalcitrant nodes within superfamilies, we sequenced an additional 14 genes, for a total of 14.8 kb, in 432 species spanning as many subfamilies as possible. For the current study, which is aimed at the “backbone” phylogeny, all 432 species sequenced for 19 genes were included. To these we added 33 species sequenced only for the five genes of Regier et al. [4], and 18 species sequenced only for a set of 8 genes described below. These 51 additional species represent subfamilies and families for which we had few or no species among the taxa sequenced for 19 genes. The 483-taxon total sample spans 45 of the 47 superfamilies (96%), 115 of the 126 families (91%), and 303 of the 344 subfamilies (88%) in the Lepidoptera classification of Kristensen [7], the morphology-based working hypothesis that we originally set out to test. A complete list of lepidopteran species sampled and their distribution across that classification (as slightly modified by van Nieurkerken et al. [1]) is given in Table S3. As outgroups, our sample also includes 8 species of Trichoptera, the sister group of Lepidoptera, representing 8 families, 6 superfamilies, both suborders and all infra-orders in the classification of Holzenthal et al. [56]. A summary of the numbers of lepidopteran species sampled across superfamilies can be found in Figure 3. DNA 'barcodes' were generated for all taxa, either by us using standard primer sequences with M13 tails [57] or, more typically, by the All-Leps Barcode of Life project (http://www.lepbarcoding.org). COI DNA 'barcodes' were checked against the BOLD (Barcode of Life Data system) [58] reference library to confirm specimen identifications and also to facilitate future identification of specimens whose identity is still pending, i.e., species listed as 'sp.' or 'unidentified' in this report. Our rationale for not including the COI data in our phylogenetic analyses has already been published [4].

Species-specific templates for mRNA amplification were prepared by extracting total nucleic acids, typically from parts of single specimens that had been stored in approximately 100% ethanol at –80° C (described in [17]). Extracted nucleic acids were stored at −80° C in diethyl-pyrocarbonate-treated deionized water. This solution was prepared by adding diethyl pyrocarbonate to 0.1% (v/v) in a glass bottle, shaking vigorously and incubating at 37° C for 16 hours, followed by steam sterilization to destroy the diethyl pyrocarbonate. Although most specimens had been stored in ethanol before or immediately after death, for a few taxa, the only material we could get had been dried, in air or in silica gel, for several days to several years before we acquired them. Of the twelve such specimens included in our taxon sample (see Table S3), 19 genes were attempted for eight, 8 genes were attempted for two, and five genes were attempted for two. The average numbers of base pairs obtained were 6787, 3695 and 2738 for 19, 8 and 5 genes respectively, about half the corresponding averages for alcohol-preserved material. These data may reflect, as least partially, amplification of genomic DNA.

### Gene sampling, amplification, and sequencing

Previously, 26 protein-coding nuclear genes were characterized and used in a phylogenetic study of 41 ditrysian Lepidoptera [4], [6], [17]. Nineteen of these genes (14658 characters total after removal of a 1098-character-long alignment mask -- many of the 1098 characters were gap characters from numerous taxa) were selected for sequencing of 391 additional taxa for a total of 432 19-gene taxa, based on information from that previous study about their consistency in generating high-quality sequences and their satisfactory degree of sequence variability. Gene names / functions and full lengths of the individual gene regions have already been published (see Table S1 of [11]), and are repeated here in Table S4. The 8-gene set referred to above, the only sequences generated for 18 of our species, was chosen for its relatively high amplification success rates and phylogenetic utility in samples which were too small or too degraded to reliably sequence for 19 genes. The eight genes, in the nomenclature of Regier et al. [11] Cho et al. [6] are: 109fin (573 bp with masked characters excluded), 265fin (447 bp), 268fin (768 bp), 3007fin (621 bp), ACC (501 bp), CAD (2865 bp), DDC (1281 bp) and Enolase (1134 bp). GenBank numbers for all sequences and taxon codenames are listed in Table S3. The absolute number of basepairs and the percentage completeness of the sequence obtained for each gene region in each species is shown in Table S5.

A detailed protocol of all laboratory procedures is available, including mRNA sequence amplification and gel isolation strategies, primer sequences, and sequence assembly and alignment methods ([22]; see also [4], [17], [59]). To summarize, specific regions of the cognate mRNAs were amplified by reverse transcription followed by PCR. Specific bands were gel isolated and reamplified by PCR using heminested primers, when available. Visible bands that were too faint to sequence were reamplified using as primers the M13 sequences at the 5’ ends of all gene-specific primers. PCR amplicons were sequenced directly on a 3730 DNA Analyzer (Applied Biosystems). Sequences were edited and assembled using the TREV, PREGAP4, and GAP4 programs in the STADEN package (Staden 1999). Individual sequences were concatenated, and alignments were made automatically using the "Translation Align" software in the Geneious Pro v. 5.3.4 package [60]. In the alignment process, splitting of individual codons was not allowed.

### Data set encoding

Three distinct data sets that include all sequences from all 483 taxa were constructed. The first one consists of unaltered nucleotides from all three nucleotide positions (nt123), analyzed as such after removal of the ambiguously aligned mask characters (Dataset S1). The second (nt123_partition) contains the same nucleotides, but they are partitioned into two non-overlapping character sets that separate nonsynonymous-only and mostly synonymous change. These two complementary character sets are called noLRall1nt2 and LRall1nt3 (see Table 1 in [24] for complete definitions; also see http://www.phylotools.com]. We chose this 1-partition procedure over the more common 2-partition procedure of separating nucleotides by codon position because the approach is simpler, having only two character sets, and yet generates a larger nonsynonymous-only set. Scripts to generate the two character sets are freely available (appendix 4 of [22], http://www.phylotools.com].

The third data set (nt123_degen1; Dataset S2) is based on the degen1 approach [23], in which in-frame codons of the same amino acid are fully degenerated with respect to synonymous change, e.g., CAT --> CAY. Leu codons (TTR + CTN) are degenerated to Leu + Phe (YTN), and Arg codons (AGR + CGN) are degenerated to Arg + Ser2 (MGN). Phe and Ser2 are degenerated to TTY and AGY, respectively. The basic idea of the degen1 approach is to capture the nonsynonymous signal while excluding the synonymous signal. When the degen1 approach is applied to the nt123 data set, we say that it yields the "nt123_degen1 data set". The degen1 script is freely available ([22], [25], http://www.phylotools.com). Other versions of degeneracy coding, including that for other genetic codes, e.g., mitochondrial, are also available at http://www.phylotools.com.

### Phylogenetic analysis of 483 taxa

An earlier study [6] found little evidence of inter-gene conflict in single-gene bootstrap analyses of a subset of 41 of the taxa used here. For this reason it seemed reasonable to concatenate the sequences for phylogenetic analysis in this study. All phylogenetic analyses are based on the Maximum Likelihood criterion applied to nucleotides, as implemented in a parallelized test version of GARLI 2.0 [18] that is available through the grid computing resources of The Lattice Project [19], [61]–[63] at the University of Maryland. The program was used with and without the character partitioning feature, always under the GTR+G+I model. Typically, the same starting topology was specified for both ML and bootstrap analyses, namely, the strict consensus from a Maximum Parsimony heuristic search of the non-bootstrapped data set obtained using PAUP*4.0 [64]. Other GARLI settings were default values. The number of heuristic search replicates for the ML topology in the analysis of nt123, nt123_partition, and nt123_degen1 for 483 taxa was 977, 250, and 4608, respectively. In the case of nt123_degen1, a further 561 search replicates were performed, using the best topology from the 4608 searches as a new starting topology. Tree files in Nexus format that define the nt123 and nt123_degen1 topologies of highest recovered likelihood, including branch lengths, can be found in Texts S2 and S3, respectively. For bootstrap analyses, the number of search replicates per bootstrap pseudoreplicate was 15, in these and all phylogenetic analyses presented herein, unless otherwise specified. The number of bootstrap pseudoreplicates in the analysis of nt123, nt123_partition, and nt123_degen1 for 483 taxa were approximately 500 in each case. For phylogenetic analyses of data sets with fewer than 483 taxa (but excluding those for the Tineoidea test taxa, see below), the numbers of ML and bootstrap search replicates were each approximately 500. For heuristic purposes only, we refer to bootstrap values ≥80% as "strong" and those from 70–79% as "moderate".

### Stability analysis and identification of rogue taxa

"Rogue" taxa have been described as those that destabilize an otherwise optimal topology, resulting in lower bootstrap support for robust or well-established clades [65], [66]. To test for a putative rogue effect in the GARLI analysis of our nt123 and nt123_degen1 data sets for 483 taxa, we undertook a systematic deletion of taxa in order to look for higher-level nodes whose bootstrap support thereby increased. Two distinct approaches were taken in identifying taxa for deletion. The first method uses RogueNaRok (the RNR approach; [67], [68], which implements the so-called relative bipartition information criterion to identify rogue taxa for subsequent deletion when given bootstrap results from a RAxML analysis. This was performed in a recursive fashion until no new rogues were identified. The second approach, called the Adams-consensus approach, is based on a visual examination of Adams consensus trees from the nt123 and nt123_degen1 bootstrap analyses, and was restricted to taxa within Apoditrysia (as newly defined herein). Taxa are removed that do not cluster with other members of their own superfamily or that are unique exemplars of a family (e.g., Cimeliidae and Doidae) that cluster with multiple superfamilies. Taxa identified as rogues by both approaches are separately listed in Text S1.

A second general approach, not designed to directly identify destabilizing taxa but instead to minimize their effects without loss of information to ingroup taxa, was to remove distant outgroups. This was done in two separate and nested deletions, leaving taxa within, and only within: Apoditrysia (as newly defined herein) and Macroheterocera (as newly defined herein) + Pyraloidea.

A third, highly targeted approach was to delete two taxa (Aun2_ACAN_ACAN, Nmec_NEOP_NEOP) found near the base of the Lepidoptera (hence, outside Apoditrysia) that seemed problematic in 483-taxon analyses (both nt123 and nt123_degen1), among others, based on low bootstrap values in their surrounding topological regions and in the Adams consensuses.

### Assessment of and dealing with compositional heterogeneity

Nucleotide compositional heterogeneity has been quantified through pairwise Euclidean distances calculated on just the proportions of the four nucleotides in the combined sequences for each taxon in the 483-taxon data matrices (nt123, nt123­_degen1) and visualized as a minimum-evolution distance tree, rooted so as to roughly minimize the presence of large groups that branch off a central backbone. These distances, based on composition alone, do not represent phylogenetic signal of the primary sequence. The length of branches is correlated with the amount of compositional heterogeneity, and the longer a compositional distance tree is, the greater is the overall compositional heterogeneity of its underlying taxon set. Compositional distance matrices were calculated with a Perl script (available at http://www.phylotools.com). Based on these matrices, distance trees were calculated in PAUP* [64] with a heuristic search under the minimum evolution criterion.

Based on inspection of these distance trees, taxa present at one end of the distance tree or the other or both were excluded so as to reduce overall heterogeneity of the remaining taxa, while still representing most of the major clades. The boundaries of exclusion were largely arbitrary. In preparing data sets, removal of "heterogeneous" taxa was always performed in combination with removal of rogue taxa.

Euclidean composition-distance trees were also generated for nt123 and nt123_degen1 from the 63 taxa in the directed study of Tineoidea (see next section). For these two "tineoid" matrices only, bootstrap values were also estimated, allowing an additional assessment of distinct compositional similarities between individual taxa beyond subtending branch lengths. For bootstrapping with 500 pseudoreplicates, 500 randomly resampled data sets and their respective compositional distance matrices were generated with a Perl script (available at http://www.phylotools.com). Bootstrap values are based on the majority rule consensus of the corresponding distance trees.

"Heterogeneous" taxa were also removed in the directed study of Tineoidea.

### Directed study of Tineoidea

As described in Results, a comparison of the 483-taxon analyses of nt123 and nt123_degen1 data sets reveals strongly supported conflicts in the placement of Tineoidea relative to the other Ditrysia. In light of the computational challenges of working with the complete data sets, we felt (and subsequently confirmed) that in this case a thorough examination of the underlying problem could still be effective when working with fewer taxa. So, we created nt123 and nt123_degen1 data sets reduced to 63 taxa. All 38 tineoids present in the 483 taxa remained. However, the outgroup was reduced to two groups positioned close to the base of Ditrysia (and Tineoidea), namely Palaephatidae (2 spp.) and Tischeriidae (3 spp.). Non-tineoid Ditrysia consisted of Gracillarioidea (6 spp.), Yponomeutoidea (7 spp.), Choreutidae (3 spp.), Urodidae (1 sp.), Schreckensteinioidea (1 sp.), Douglasiidae (1), Millieridae (1), Immidae (1 sp.), Tortricidae (2 spp.), Gelechioidea (2 spp.), Cossoidea (1 sp.), Zygaenoidea (1 sp.), and Hyblaeoidea (1 sp.). These 63-taxon data sets were analyzed by ML and bootstrap analyses through a series of taxon deletions. The number of ML search replicates performed was approximately 1000, while the number of bootstrap pseudoreplicates was approximately 750.

## Supporting Information

Figure S1 **Maximum likelihood tree in phylogram format, with bootstrap values, based on analysis of the nt123_degen1 data set for 483 taxa and 19 genes.** A condensed cladogram version is shown in Figure 2. Terminal taxa are labeled by their generic names. Higher-level classification names are also included. The 63 tineoid test taxa are each identified by three asterisks placed after their generic names.
(PDF)

Figure S2 **Maximum likelihood tree in phylogram format, with bootstrap values, based on analysis of the nt123 data set for 483 taxa and 19 genes.** Terminal taxa are labeled by their generic name. Higher-level classification names are also included.
(PDF)

Table S1 **Bootstrap results based on analysis of taxon-depleted nt123_degen1 data sets.**
(PDF)

Table S2 **Bootstrap results based on analysis of taxon-depleted nt123 data sets.**
(PDF)

Table S3 **List of specimens sampled, Leptree voucher identification numbers, and gene information, including GenBank numbers.**
(XLS)

Table S4 **Synopsis of genes sequenced.**
(PDF)

Table S5 **Absolute number of unambiguous nucleotides (bp) per gene in each taxon, plus summary statistics.**
(PDF)

Text S1 **List of taxon subsets used to generate (by deletion) new data sets with reduced numbers of taxa.**
(DOC)

Text S2 **Nexus-formatted tree file that encodes the topology (with branch lengths) of highest likelihood recovered in our analysis of the nt123 data set for 483 taxa and 19 genes with mask characters already excluded.** The species codenames are identified by their complete genus-species names in Table S3.
(TRE)

Text S3 **Nexus-formatted tree file that encodes the topology (with branch lengths) of highest likelihood recovered in our analysis of the nt123_degen1 data set for 483 taxa and 19 genes with mask characters already excluded.** The species codenames are identified by their complete genus-species names in Table S3.
(TRE)

Dataset S1 **Nexus-formatted data set that includes nucleotide sequence data (nt123) for 483 taxa and 19 genes with the ambiguously aligned characters already excluded (14658 characters total).** Sets of characters are defined and listed immediately after the data matrix. This data set can be degenerated using the degen1 script available at http://www.phylotools.com. The species codenames are identified by their complete genus-species names in Table S3.
(NEX)

Dataset S2 **Nexus-formatted data set that includes nucleotide sequence data (nt123_degen1) for 483 taxa and 19 genes with the ambiguously aligned characters already excluded (14658 characters total).** This data set was degenerated using a degen1 script and the nt123 data set. The most current degen1 script is available at http://www.phylotools.com. The species codenames are identified by their complete genus-species names in Table S3.
(NEX)
