# Supplementary material for: A Large-Scale, Higher-Level, Molecular Phylogenetic Study of the Insect Order Lepidoptera (Moths and Butterflies)
Source: PLoS One. 2013 Mar 12;8(3):e58568. doi: 10.1371/journal.pone.0058568 (PMC3595289; doi:10.1371/journal.pone.0058568)
Supplement: Figure S1 — Maximum likelihood tree in phylogram format, with bootstrap values, based on analysis of the nt123_degen1 data set for 483 taxa and 19 genes. A condensed cladogram version is shown in Figure 2. Terminal taxa are labeled by their generic names. Higher-level classification names are also included. The 63 tineoid test taxa are each identified by three asterisks placed after their generic names. (PDF) [file pone.0058568.s001.pdf]

Figure S1. Maximum likelihood tree in phylogram format, with bootstrap values, based based on analysis of the nt123_degen1 data set for 483 taxa and 19 genes. The cladogram version (without terminal taxon names) is shown in Figure 2. Terminal taxa are labeled by their generic name. Higher-level classification names are also included. Three consecutive asterisks are placed after the generic names of each of the 63 tineoid test taxa.

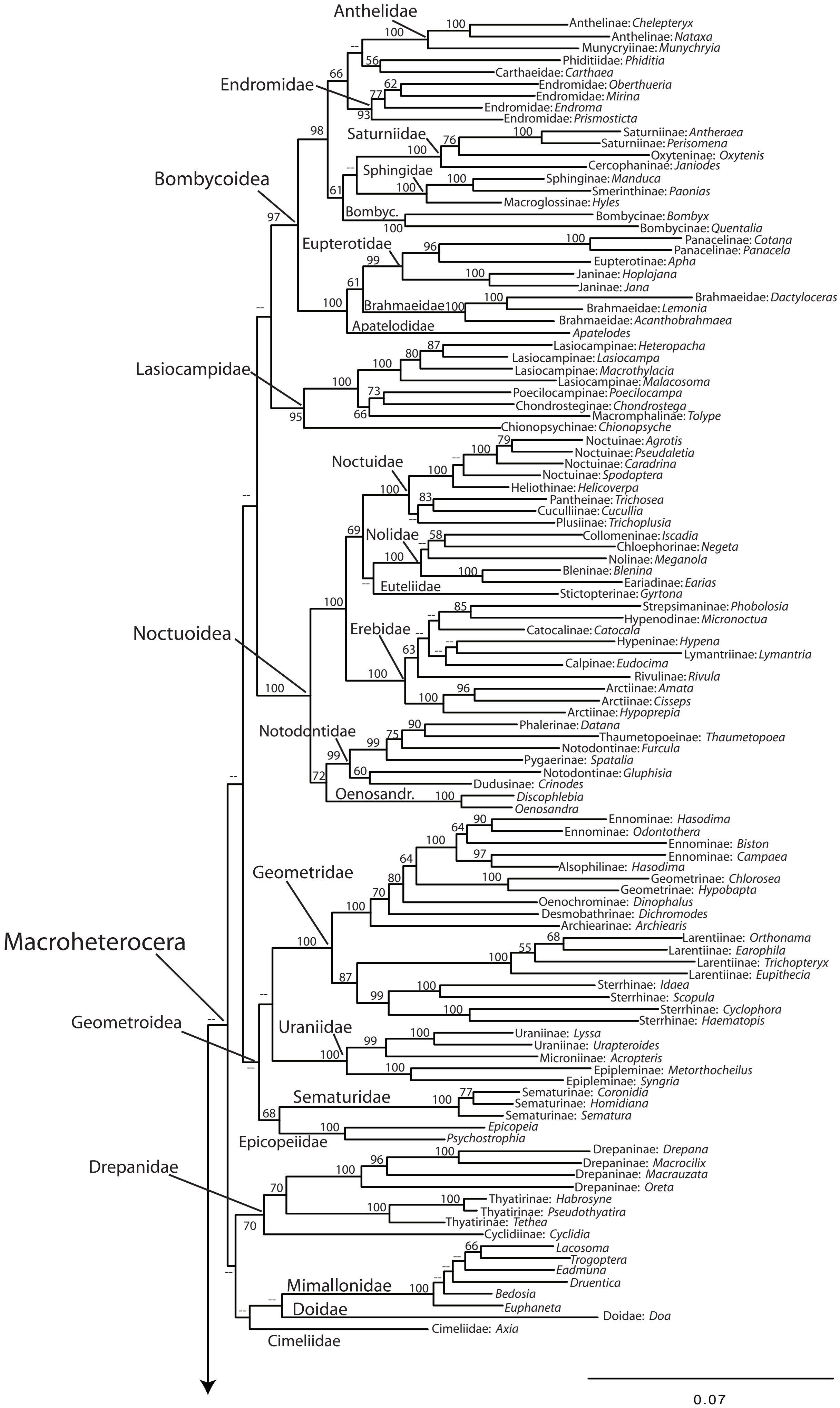

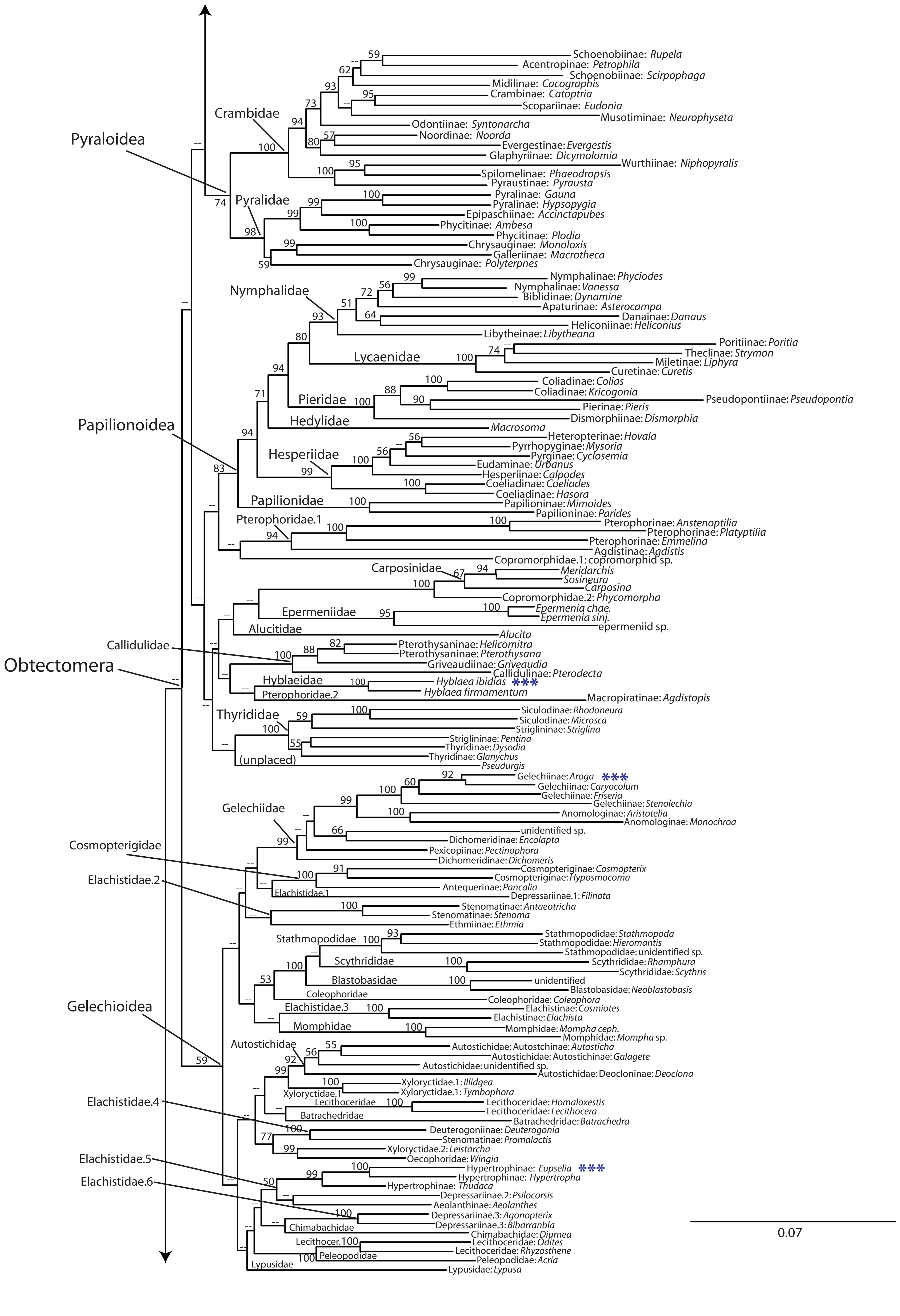
Pyraloidea
Crambidae
Schoenobiinae: Rupela
Acentropinae: Petrophila
Schoenobiinae: Scirpophaga
Midilinae: Cacographis
Crambinae: Catoptria
Scopariinae: Eudonia
Musotiminae: Neurophyseta
Odontiinae: Syntonarcha
Noordinae: Noorda
Evergestinae: Evergestis
Glaphyriinae: Dicymolomia
Wurthiinae: Niphopyralis
Spilomelinae: Phaeodropsis
Pyraustinae: Pyrausta
Pyralidae
Pyralinae: Gauna
Pyralinae: Hypsopygia
Epipaschiinae: Accinctapubes
Phycitinae: Ambesa
Phycitinae: Plodia
Chrysauginae: Monoloxis
Galleriinae: Macrotheca
Chrysauginae: Polyterpnes
Nymphalidae
Nymphalinae: Phyciodes
Nymphalinae: Vanessa
Biblidinae: Dynamine
Apaturinae: Asterocampa
Danainae: Danaus
Heliconiinae: Heliconius
Libytheinae: Libytheana
Lycaenidae
Poritiinae: Poritia
Theclinae: Strymon
Miletinae: Liphyra
Curetinae: Curetis
Pieridae
Coliadinae: Colias
Coliadinae: Kricogonia
Pseudopontiinae: Pseudopontia
Pierinae: Pieris
Dismorphiinae: Dismorphia
Hedylidae
Macrosoma
Papilionoidea
Hesperiidae
Heteropterinae: Hovala
Pyrrhopyginae: Mysoria
Pyrginae: Cyclosemia
Eudaminae: Urbanus
Hesperiinae: Calpodes
Coeliadinae: Coeliades
Coeliadinae: Hasora
Papilionidae
Papilioninae: Mimoides
Papilioninae: Parides
Pterophoridae.1
Pterophorinae: Anstenoptilia
Pterophorinae: Platyptilia
Pterophorinae: Emmelina
Agdistinae: Agdistis
Copromorphidae.1: copromorphid sp.
Carposinidae
Meridarchis
Sosineura
Carposina
Copromorphidae.2: Phycomorpha
Epermeniidae
Epermenia chae.
Epermenia sinj.
epermeniid sp.
Alucitidae
Alucita
Callidulidae
Pterothysaninae: Helicomitra
Pterothysaninae: Pterothysana
Griveaudiinae: Griveaudia
Callidulinae: Pterodecta
Obtectomera
Hyblaeidae
Hyblaea ibidias ***
Hyblaea firmamentum
Pterophoridae.2
Macropiratinae: Agdistopis
Thyrididae
Siculodinae: Rhodoneura
Siculodinae: Microsca
Striglininae: Striglina
Striglininae: Pentina
Thyridinae: Dysodia
Thyridinae: Glanychus
(unplaced)
Pseudurgis
Gelechiidae
Gelechiinae: Aroga ***
Gelechiinae: Caryocolum
Gelechiinae: Friseria
Gelechiinae: Stenolechia
Anomologinae: Aristotelia
Anomologinae: Monochroa
unidentified sp.
Dichomeridinae: Encolapta
Pexicopiinae: Pectinophora
Dichomeridinae: Dichomeris
Cosmopterigidae
Cosmopteriginae: Cosmopterix
Cosmopteriginae: Hyposmocoma
Antequerinae: Pancalia
Elachistidae.1
Depressariinae.1: Filinota
Elachistidae.2
Stenomatinae: Antaeotricha
Stenomatinae: Stenoma
Ethmiinae: Ethmia
Stathmopodidae
Stathmopodidae: Stathmopoda
Stathmopodidae: Hieromantis
Stathmopodidae: unidentified sp.
Scythrididae
Scythrididae: Rhamphura
Scythrididae: Scythris
Blastobasidae
unidentified
Blastobasidae: Neoblastobasis
Coleophoridae
Coleophoridae: Coleophora
Elachistidae.3
Elachistinae: Cosmiotes
Elachistinae: Elachista
Gelechioidea
Momphidae
Momphidae: Mompha ceph.
Momphidae: Mompha sp.
Autostichidae
Autostichidae: Autostchinae: Autosticha
Autostichidae: Autostichinae: Galagete
Autostichidae: unidentified sp.
Autostichidae: Deocloninae: Deoclona
Xyloryctidae.1
Xyloryctidae.1: Illidgea
Xyloryctidae.1: Tymbophora
Lecithoceridae
Lecithoceridae: Homaloxestis
Lecithoceridae: Lecithocera
Batrachedridae
Batrachedridae: Batrachedra
Elachistidae.4
Deuterogoniinae: Deuterogonia
Stenomatinae: Promalactis
Xyloryctidae.2: Leistarcha
Oecophoridae: Wingia
Elachistidae.5
Hypertrophinae: Eupselia ***
Hypertrophinae: Hypertropha
Hypertrophinae: Thudaca
Depressariinae.2: Psilocorsis
Aeolanthinae: Aeolanthes
Elachistidae.6
Depressariinae.3: Agonopterix
Depressariinae.3: Bibarranbla
Chimabachidae
Chimabachidae: Diurnea
Lecithocer.
Lecithoceridae: Odites
Lecithoceridae: Rhyzosthene
Peleopodidae
Peleopodidae: Acria
Lypusidae
Lypusidae: Lypusa
0.07

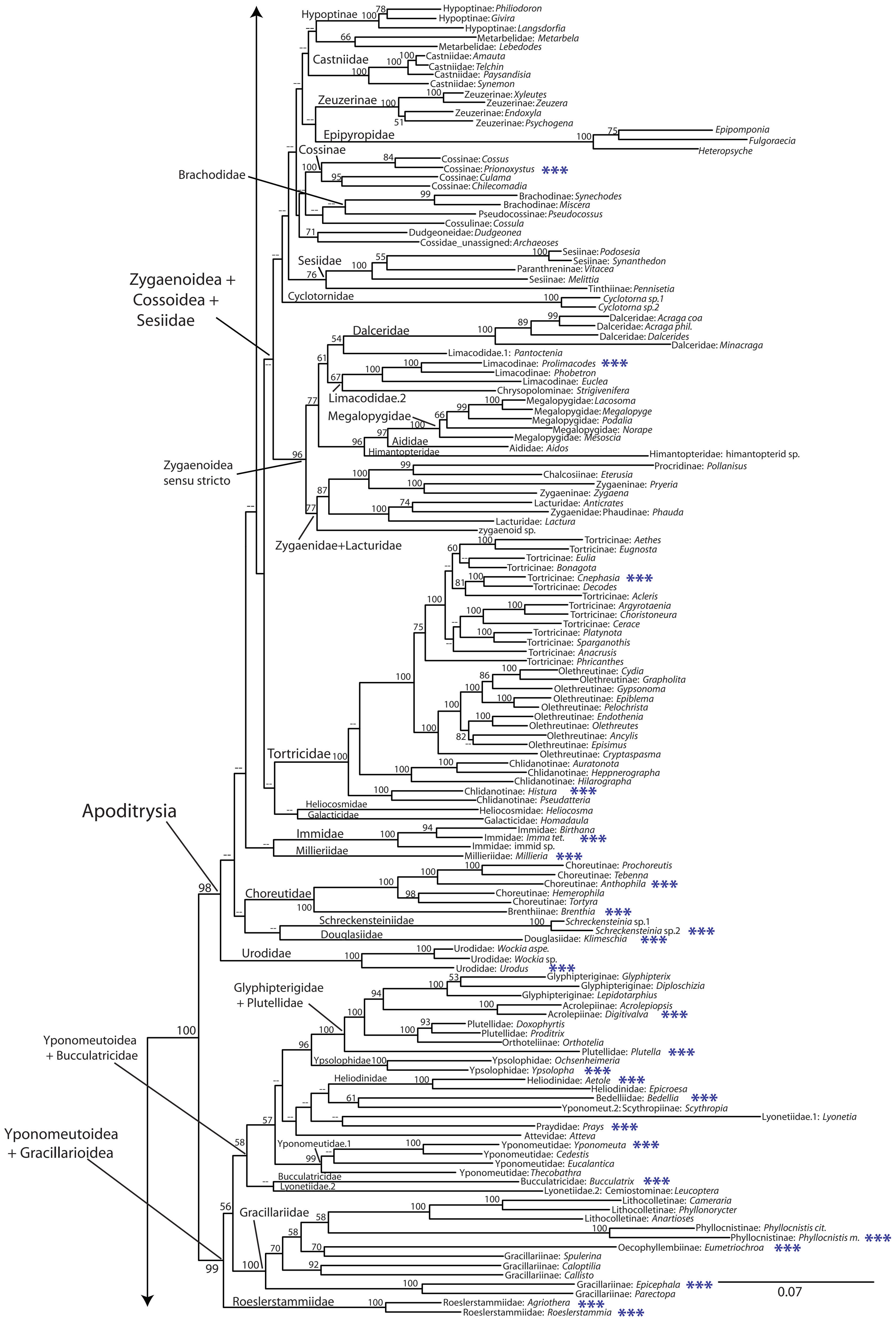

Hypoptinae
Hypoptinae: Philiodoron
Hypoptinae: Givira
Hypoptinae: Langsdorfia
Metarbelidae: Metarbela
Metarbelidae: Lebedodes
Castniidae
Castniidae: Amauta
Castniidae: Telchin
Castniidae: Paysandisia
Castniidae: Synemon
Zeuzerinae
Zeuzerinae: Xyleutes
Zeuzerinae: Zeuzera
Zeuzerinae: Endoxyla
Zeuzerinae: Psychogena
Epipyropidae
Epipomponia
Fulgoraecia
Heteropsyche
Cossinae
Cossinae: Cossus
Cossinae: Prionoxystus
Cossinae: Culama
Cossinae: Chilecomadia
Brachodidae
Brachodinae: Synechodes
Brachodinae: Miscera
Pseudocossinae: Pseudocossus
Cossulinae: Cossula
Dudgeoneidae: Dudgeonea
Cossidae_unassigned: Archaeoses
Sesiidae
Sesiinae: Podosesia
Sesiinae: Synanthedon
Paranthreninae: Vitacea
Sesiinae: Melittia
Tinthiinae: Pennisetia
Cyclotornidae
Cyclotorna sp.1
Cyclotorna sp.2
Zygaenoidea + Cossoidea + Sesiidae
Dalceridae
Dalceridae: Acraga coa
Dalceridae: Acraga phil.
Dalceridae: Dalcerides
Dalceridae: Minacraga
Limacodidae.1: Pantoctenia
Limacodinae: Prolimacodes
Limacodinae: Phobetron
Limacodinae: Euclea
Chrysopolominae: Strigivenifera
Limacodidae.2
Megalopygidae
Megalopygidae: Lacosoma
Megalopygidae: Megalopyge
Megalopygidae: Podalia
Megalopygidae: Norape
Megalopygidae: Mesoscia
Aididae
Aididae: Aidos
Himantopteridae
Himantopteridae: himantopterid sp.
Zygaenoidea sensu stricto
Procridinae: Pollanisus
Chalcosiinae: Eterusia
Zygaeninae: Pryeria
Zygaeninae: Zygaena
Lacturidae: Anticrates
Zygaenidae: Phaudinae: Phauda
Lacturidae: Lactura
zygaenoid sp.
Zygaenidae+Lacturidae
Tortricinae: Aethes
Tortricinae: Eugnosta
Tortricinae: Eulia
Tortricinae: Bonagota
Tortricinae: Cnephasia
Tortricinae: Decodes
Tortricinae: Acleris
Tortricinae: Argyrotaenia
Tortricinae: Choristoneura
Tortricinae: Cerace
Tortricinae: Platynota
Tortricinae: Sparganothis
Tortricinae: Anacrusis
Tortricinae: Phricanthes
Olethreutinae: Cydia
Olethreutinae: Grapholita
Olethreutinae: Gypsonoma
Olethreutinae: Epiblema
Olethreutinae: Pelochrista
Olethreutinae: Endothenia
Olethreutinae: Olethreutes
Olethreutinae: Ancylis
Olethreutinae: Episimus
Olethreutinae: Cryptaspasma
Tortricidae
Chlidanotinae: Auratonota
Chlidanotinae: Heppnerographa
Chlidanotinae: Hilarographa
Chlidanotinae: Histura
Chlidanotinae: Pseudatteria
Heliocosmidae
Galacticidae
Heliocosmidae: Heliocosma
Galacticidae: Homadaula
Apoditrysia
Immidae
Immidae: Birthana
Immidae: Imma tet.
Immidae: immid sp.
Millieriidae
Millieriidae: Millieria
Choreutidae
Choreutinae: Prochoreutis
Choreutinae: Tebenna
Choreutinae: Anthophila
Choreutinae: Hemerophila
Choreutinae: Tortyra
Brenthiinae: Brenthia
Schreckensteiniidae
Schreckensteinia sp.1
Schreckensteinia sp.2
Douglasiidae
Douglasiidae: Klimeschia
Urodidae
Urodidae: Wockia aspe.
Urodidae: Wockia sp.
Urodidae: Urodus
Glyphipterigidae + Plutellidae
Glyphipteriginae: Glyphipterix
Glyphipteriginae: Diploschizia
Glyphipteriginae: Lepidotarphius
Acrolepiinae: Acrolepiopsis
Acrolepiinae: Digitivalva
Plutellidae: Doxophyrtis
Plutellidae: Proditrix
Ortholeliinae: Orthotelia
Plutellidae: Plutella
Ypsolophidae
Ypsolophidae: Ochsenheimeria
Ypsolophidae: Ypsolopha
Yponomeutoidea + Bucculatricidae
Heliodinidae
Heliodinidae: Aetole
Heliodinidae: Epicroesa
Bedelliidae: Bedellia
Yponomeut.2: Scythropiinae: Scythropia
Lyonetiidae.1: Lyonetia
Praydidae: Prays
Attevidae: Atteva
Yponomeutoidea + Gracillarioidea
Yponomeutidae.1
Yponomeutidae: Yponomeuta
Yponomeutidae: Cedestis
Yponomeutidae: Eucalantica
Yponomeutidae: Thecobathra
Bucculatricidae
Bucculatricidae: Bucculatrix
Lyonetiidae.2
Lyonetiidae.2: Cemiostominae: Leucoptera
Gracillariidae
Lithocolletinae: Cameraria
Lithocolletinae: Phyllonorycter
Lithocolletinae: Anartioses
Phyllocnistinae: Phyllocnistis cit.
Phyllocnistinae: Phyllocnistis m.
Oecophyllembiinae: Eumetriochroa
Gracillariinae: Spulerina
Gracillariinae: Caloptilia
Gracillariinae: Callisto
Gracillariinae: Epicephala
Gracillariinae: Parectopa
Roeslerstammiidae
Roeslerstammiidae: Agriothera
Roeslerstammiidae: Roeslerstammia
0.07

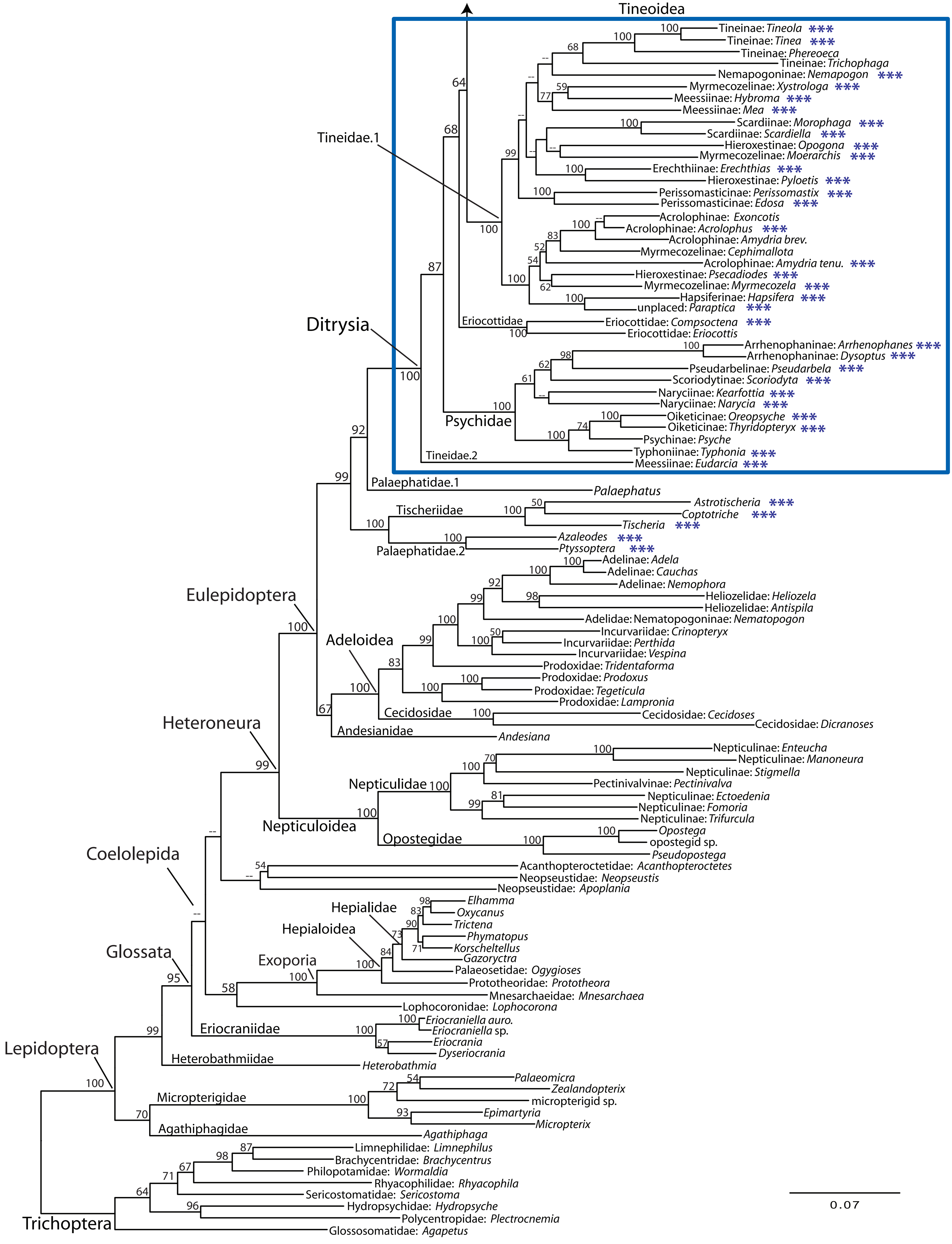

Tineoidea
Tineinae: Tineola ***
Tineinae: Tinea ***
Tineinae: Phereoeca
Tineinae: Trichophaga
Nemapogoninae: Nemapogon ***
Myrmecozelinae: Xystrologa ***
Meessiinae: Hybroma ***
Meessiinae: Mea ***
Scardiinae: Morophaga ***
Scardiinae: Scardiella ***
Hieroxestinae: Opogona ***
Myrmecozelinae: Moerarchis ***
Erechthiinae: Erechthias ***
Hieroxestinae: Pyloetis ***
Perissomasticinae: Perissomastix ***
Perissomasticinae: Edosa ***
Acrolophinae: Exoncotis
Acrolophinae: Acrolophus ***
Acrolophinae: Amydria brev.
Myrmecozelinae: Cephimallota
Acrolophinae: Amydria tenu. ***
Hieroxestinae: Psecadiodes ***
Myrmecozelinae: Myrmecozela ***
Hapsiferinae: Hapsifera ***
unplaced: Paraptica ***
Eriocottidae
Eriocottidae: Compsoctena ***
Eriocottidae: Eriocottis
Arrhenophaninae: Arrhenophanes ***
Arrhenophaninae: Dysoptus ***
Pseudarbelinae: Pseudarbela ***
Scoriodytinae: Scoriodyta ***
Naryciinae: Kearfottia ***
Naryciinae: Narycia ***
Psychidae
Oiketicinae: Oreopsyche ***
Oiketicinae: Thyridopteryx ***
Psychinae: Psyche
Typhoniinae: Typhonia ***
Tineidae.1
Tineidae.2
Meessiinae: Eudarcia ***
Ditrysia
Palaephatidae.1
Palaephatus
Tischeriidae
Astrotischeria ***
Coptotriche ***
Tischeria ***
Azaleodes ***
Ptyssoptera ***
Palaephatidae.2
Eulepidoptera
Adelinae: Adela
Adelinae: Cauchas
Adelinae: Nemophora
Heliozelidae: Heliozela
Heliozelidae: Antispila
Adelidae: Nematopogoninae: Nematopogon
Adeloidea
Incurvariidae: Crinopteryx
Incurvariidae: Perthida
Incurvariidae: Vespina
Prodoxidae: Tridentaforma
Prodoxidae: Prodoxus
Prodoxidae: Tegeticula
Prodoxidae: Lampronia
Cecidosidae
Cecidosidae: Cecidoses
Cecidosidae: Dicranoses
Andesianidae
Andesiana
Heteroneura
Nepticulinae: Enteucha
Nepticulinae: Manoneura
Nepticulinae: Stigmella
Pectinivalvinae: Pectinivalva
Nepticulidae
Nepticulinae: Ectoedenia
Nepticulinae: Fomoria
Nepticulinae: Trifurcula
Nepticuloidea
Opostegidae
Opostega
opostegid sp.
Pseudopostega
Acanthopteroctetidae: Acanthopteroctetes
Neopseustidae: Neopseustis
Neopseustidae: Apoplania
Coelolepida
Elhamma
Oxycanus
Hepialidae
Trictena
Hepialoidea
Phymatopus
Korscheltellus
Gazoryctra
Exoporia
Palaeosetidae: Ogygioses
Prototheoridae: Prototheora
Mnesarchaeidae: Mnesarchaea
Glossata
Lophocoronidae: Lophocorona
Eriocraniidae
Eriocraniella auro.
Eriocraniella sp.
Eriocrania
Dyseriocrania
Lepidoptera
Heterobathmiidae
Heterobathmia
Palaeomicra
Zealandopterix
Micropterigidae
micropterigid sp.
Epimartyria
Micropterix
Agathiphagidae
Agathiphaga
Limnephilidae: Limnephilus
Brachycentridae: Brachycentrus
Philopotamidae: Wormaldia
Rhyacophilidae: Rhyacophila
Sericostomatidae: Sericostoma
Hydropsychidae: Hydropsyche
Polycentropidae: Plectrocnemia
Trichoptera
Glossosomatidae: Agapetus
0.07
