# Supplementary material for: A Large-Scale, Higher-Level, Molecular Phylogenetic Study of the Insect Order Lepidoptera (Moths and Butterflies)
Source: PLoS One. 2013 Mar 12;8(3):e58568. doi: 10.1371/journal.pone.0058568 (PMC3595289; doi:10.1371/journal.pone.0058568)
Supplement: Figure S2 — Maximum likelihood tree in phylogram format, with bootstrap values, based on analysis of the nt123 data set for 483 taxa and 19 genes. Terminal taxa are labeled by their generic name. Higher-level classification names are also included. (PDF) [file pone.0058568.s002.pdf]

Figure S2. Maximum likelihood tree in phylogram format, with bootstrap values, based on analysis of the *nt123* data set for 483 taxa and 19 genes. Terminal taxa are labeled by their generic name. Higher-level classification names are also included.

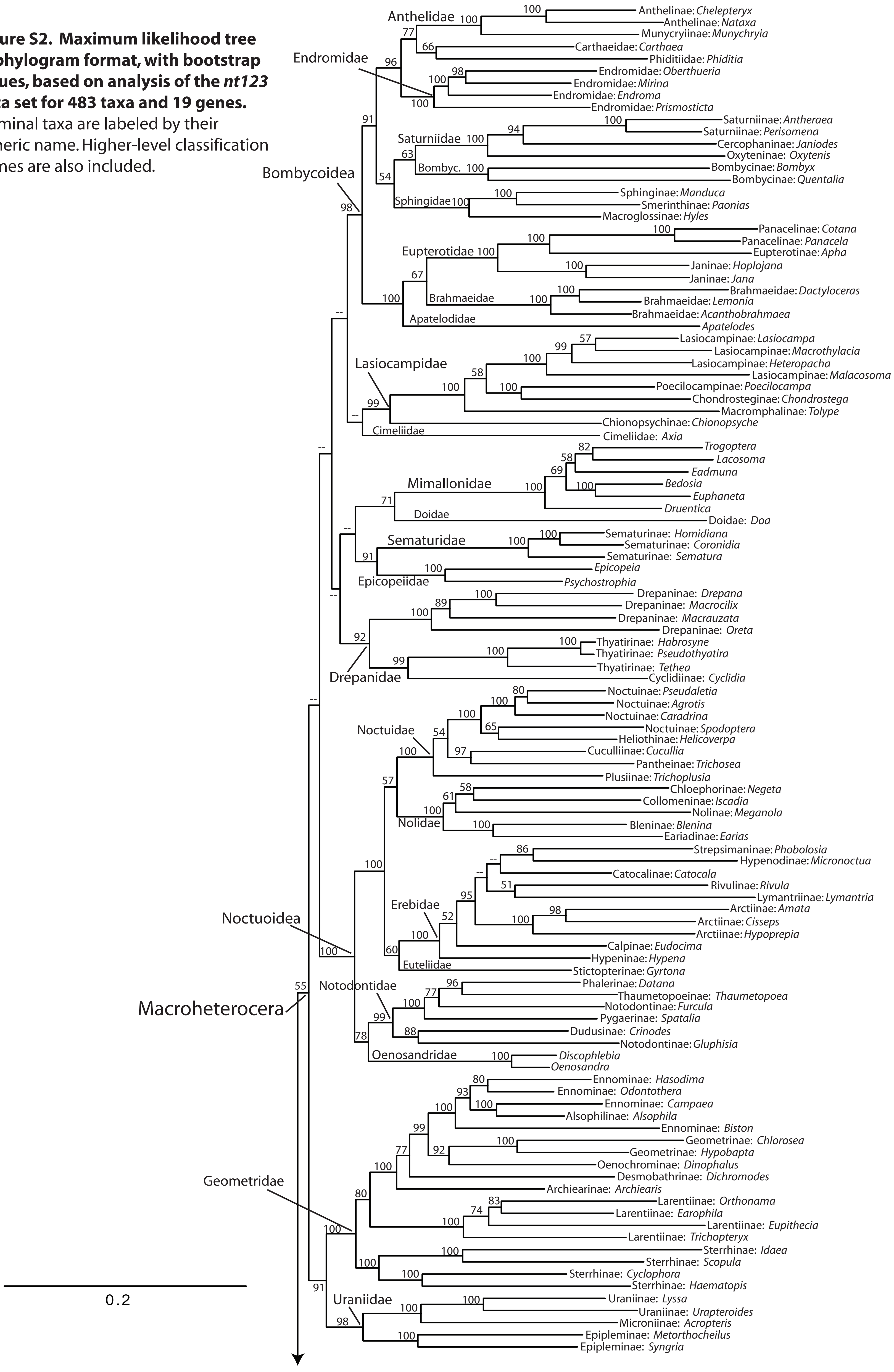

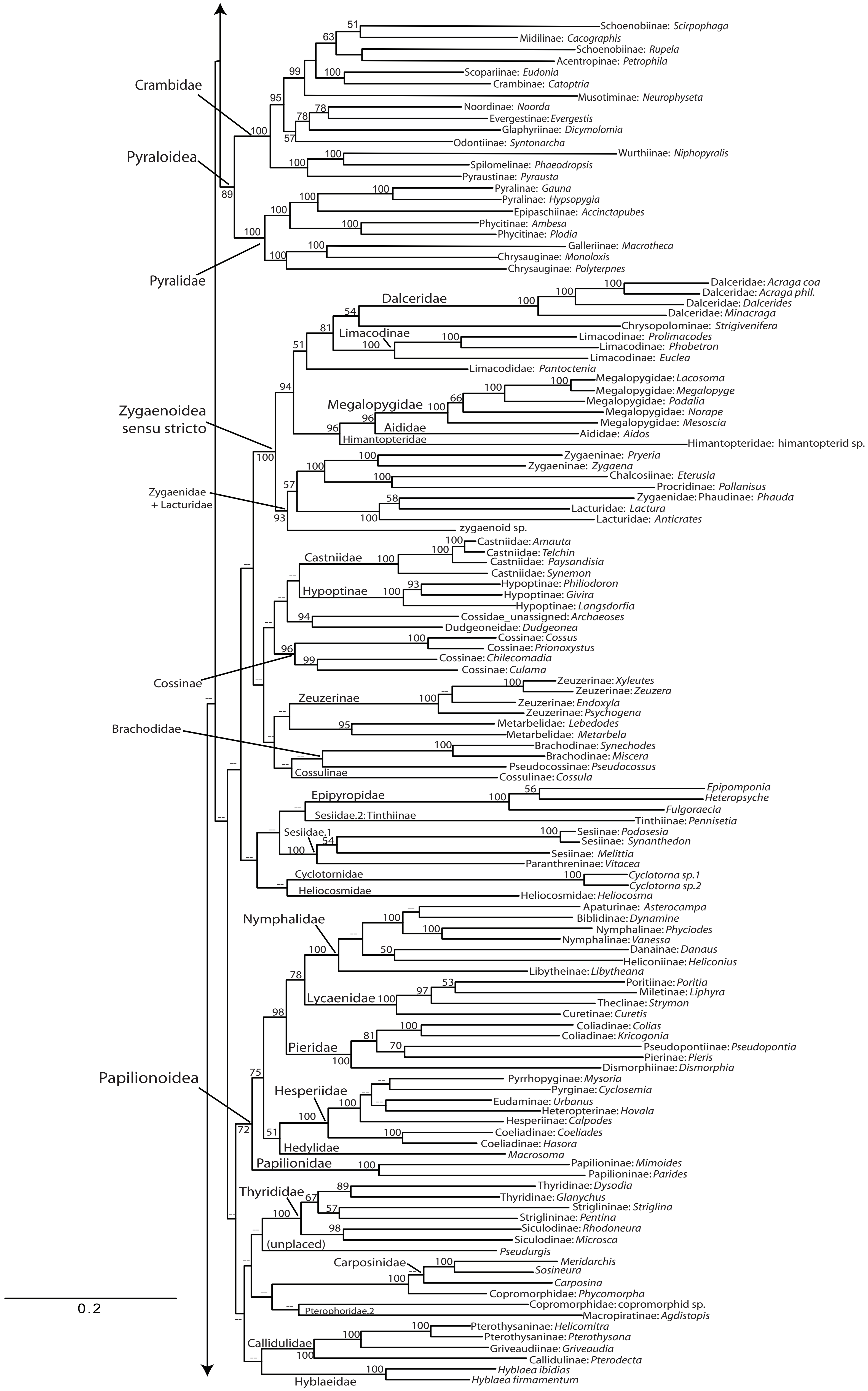

Crambidae
Pyraloidea
Pyralidae
Schoenobiinae: Scirpophaga
Midilinae: Cacographis
Schoenobiinae: Rupela
Acentropinae: Petrophila
Scopariinae: Eudonia
Crambinae: Catoptria
Musotiminae: Neurophyseta
Noordinae: Noorda
Evergestinae: Evergestis
Glaphyriinae: Dicymolomia
Odontiinae: Syntonarcha
Wurthiinae: Niphopyralis
Spilomelinae: Phaeodropsis
Pyraustinae: Pyrausta
Pyralinae: Gauna
Pyralinae: Hypsopygia
Epipaschiinae: Accinctapubes
Phycitinae: Ambesa
Phycitinae: Plodia
Galleriinae: Macrotheca
Chrysauginae: Monoloxis
Chrysauginae: Polyterpnes
Zygaenoidea sensu stricto
Dalceridae
Limacodinae
Megalopygidae
Aidiidae
Himantopteridae
Zygaenidae + Lacturidae
Dalceridae: Acraga coa
Dalceridae: Acraga phil.
Dalceridae: Dalcerides
Dalceridae: Minacraga
Chrysopolominae: Strigivenifera
Limacodinae: Prolimacodes
Limacodinae: Phobetron
Limacodinae: Euclea
Limacodidae: Pantoctenia
Megalopygidae: Lacosoma
Megalopygidae: Megalopyge
Megalopygidae: Podalia
Megalopygidae: Norape
Megalopygidae: Mesoscia
Aididae: Aidos
Himantopteridae: himantopterid sp.
Zygaeninae: Pryeria
Zygaeninae: Zygaena
Chalcosiinae: Eterusia
Procridinae: Pollanisus
Zygaenidae: Phaudinae: Phauda
Lacturidae: Lactura
Lacturidae: Anticrates
zygaenoid sp.
Castniidae
Hypoptinae
Cossinae
Zeuzerinae
Brachodidae
Cossulinae
Castniidae: Amauta
Castniidae: Telchin
Castniidae: Paysandisia
Castniidae: Synemon
Hypoptinae: Philiodoron
Hypoptinae: Givira
Hypoptinae: Langsdorfia
Cossidae_unassigned: Archaeoses
Dudgeoneidae: Dudgeonea
Cossinae: Cossus
Cossinae: Prionoxystus
Cossinae: Chilecomadia
Cossinae: Culama
Zeuzerinae: Xyleutes
Zeuzerinae: Zeuzera
Zeuzerinae: Endoxyla
Zeuzerinae: Psychogena
Metarbelidae: Lebedodes
Metarbelidae: Metarbela
Brachodinae: Synechodes
Brachodinae: Miscera
Pseudocossinae: Pseudocossus
Cossulinae: Cossula
Epipyropidae
Sesiidae.2: Tinthiinae
Sesiidae.1
Cyclotornidae
Heliocosmidae
Epipomponia
Heteropsyche
Fulgoraecia
Tinthiinae: Pennisetia
Sesiinae: Podosesia
Sesiinae: Synanthedon
Sesiinae: Melittia
Paranthreninae: Vitacea
Cyclotorna sp.1
Cyclotorna sp.2
Heliocosmidae: Heliocosma
Nymphalidae
Lycaenidae
Pieridae
Hesperiidae
Hedylidae
Papilionidae
Papilionoidea
Apaturinae: Asterocampa
Biblidinae: Dynamine
Nymphalinae: Phyciodes
Nymphalinae: Vanessa
Danainae: Danaus
Heliconiinae: Heliconius
Libytheinae: Libytheana
Poritiinae: Poritia
Miletinae: Liphyra
Theclinae: Strymon
Curetinae: Curetis
Coliadinae: Colias
Coliadinae: Kricogonia
Pseudopontiinae: Pseudopontia
Pierinae: Pieris
Dismorphiinae: Dismorphia
Pyrrhopyginae: Mysoria
Pyrginae: Cyclosemia
Eudaminae: Urbanus
Heteropterinae: Hovala
Hesperiinae: Calpodes
Coeliadinae: Coeliades
Coeliadinae: Hasora
Macrosoma
Papilioninae: Mimoides
Papilioninae: Parides
Thyrididae
(unplaced)
Carposinidae
Pterophoridae.2
Callidulidae
Hyblaeidae
Thyridinae: Dysodia
Thyridinae: Glanychus
Striglininae: Striglina
Striglininae: Pentina
Siculodinae: Rhodoneura
Siculodinae: Microsca
Pseudurgis
Meridarchis
Sosineura
Carposina
Copromorphidae: Phycomorpha
Copromorphidae: copromorphid sp.
Macropiratinae: Agdistopis
Pterothysaninae: Helicomitra
Pterothysaninae: Pterothysana
Griveaudiinae: Griveaudia
Callidulinae: Pterodecta
Hyblaea ibidias
Hyblaea firmamentum
0.2

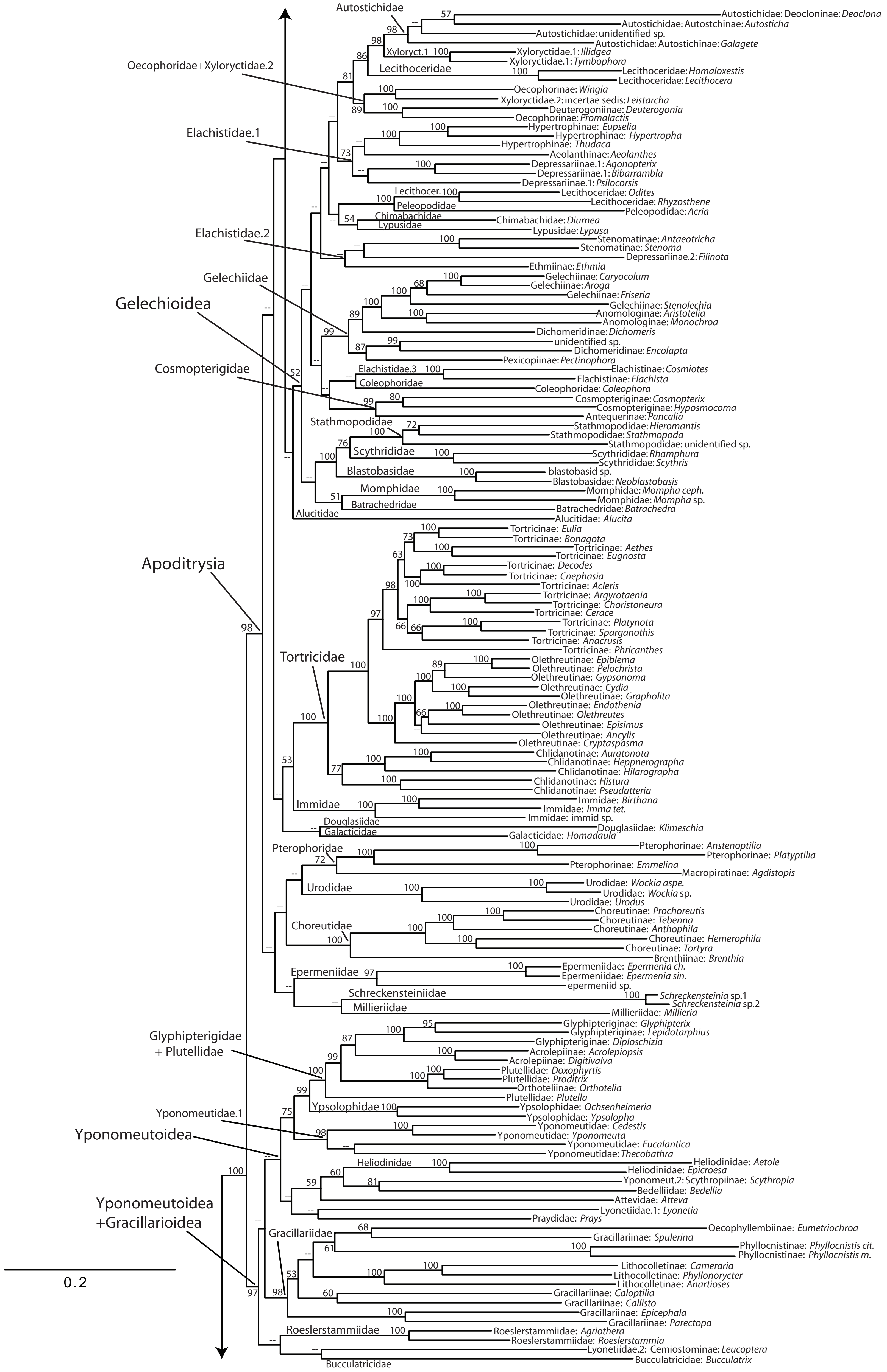

Autostichidae
Autostichidae: Deoclonae: Deoclona
Autostichidae: Autostchinae: Autosticha
Autostichidae: unidentified sp.
Autostichidae: Autostichinae: Galagete
Xyloryct.1
Xyloryctidae.1: Illidgea
Xyloryctidae.1: Tymbophora
Lecithoceridae
Lecithoceridae: Homaloxestis
Lecithoceridae: Lecithocera
Oecophoridae+Xyloryctidae.2
Oecophorinae: Wingia
Xyloryctidae.2: incertae sedis: Leistarcha
Deuterogoniinae: Deuterogonia
Oecophorinae: Promalactis
Elachistidae.1
Hypertrophinae: Eupselia
Hypertrophinae: Hypertropha
Hypertrophinae: Thudaca
Aeolanthinae: Aeolanthes
Depressariinae.1: Agonopterix
Depressariinae.1: Bibarrambla
Depressariinae.1: Psilocorsis
Lecithocer.
Lecithoceridae: Odites
Lecithoceridae: Rhyzosthene
Peleopodidae
Peleopodidae: Acria
Chimabachidae
Chimabachidae: Diurnea
Lypusidae
Lypusidae: Lypusa
Elachistidae.2
Stenomatinae: Antaeotricha
Stenomatinae: Stenoma
Depressariinae.2: Filinota
Ethmiinae: Ethmia
Gelechiidae
Gelechioidea
Gelechiinae: Caryocolum
Gelechiinae: Aroga
Gelechiinae: Friseria
Gelechiinae: Stenolechia
Anomologinae: Aristotelia
Anomologinae: Monochroa
Dichomeridinae: Dichomeris
unidentified sp.
Dichomeridinae: Encolapta
Pexicopiinae: Pectinophora
Cosmopterigidae
Elachistidae.3
Elachistinae: Cosmiotes
Elachistinae: Elachista
Coleophoridae
Coleophoridae: Coleophora
Cosmopteriginae: Cosmopterix
Cosmopteriginae: Hyposmocoma
Antequerinae: Pancalia
Stathmopodidae
Stathmopodidae: Hieromantis
Stathmopodidae: Stathmopoda
Stathmopodidae: unidentified sp.
Scythrididae
Scythrididae: Rhamphura
Scythrididae: Scythris
Blastobasidae
blastobasid sp.
Blastobasidae: Neoblastobasis
Momphidae
Momphidae: Mompha ceph.
Momphidae: Mompha sp.
Batrachedridae
Batrachedridae: Batrachedra
Alucitidae
Alucitidae: Alucita
Apoditrysia
Tortricinae: Eulia
Tortricinae: Bonagota
Tortricinae: Aethes
Tortricinae: Eugnosta
Tortricinae: Decodes
Tortricinae: Cnephasia
Tortricinae: Acleris
Tortricinae: Argyrotaenia
Tortricinae: Choristoneura
Tortricinae: Cerace
Tortricinae: Platynota
Tortricinae: Sparganothis
Tortricinae: Anacrusis
Tortricinae: Phricanthes
Tortricidae
Olethreutinae: Epiblema
Olethreutinae: Pelochrista
Olethreutinae: Gypsonoma
Olethreutinae: Cydia
Olethreutinae: Grapholita
Olethreutinae: Endothenia
Olethreutinae: Olethreutes
Olethreutinae: Episimus
Olethreutinae: Ancylis
Olethreutinae: Cryptaspasma
Chlidanotinae: Auratonota
Chlidanotinae: Heppnerographa
Chlidanotinae: Hilarographa
Chlidanotinae: Histura
Chlidanotinae: Pseudatteria
Immidae
Immidae: Birthana
Immidae: Imma tet.
Immidae: immid sp.
Douglasiidae
Galacticidae
Douglasiidae: Klimeschia
Galacticidae: Homadaula
Pterophoridae
Pterophorinae: Anstenoptilia
Pterophorinae: Platyptilia
Pterophorinae: Emmelina
Macropiratinae: Agdistopis
Urodidae
Urodidae: Wockia aspe.
Urodidae: Wockia sp.
Urodidae: Urodus
Choreutidae
Choreutinae: Prochoreutis
Choreutinae: Tebenna
Choreutinae: Anthophila
Choreutinae: Hemerophila
Choreutinae: Tortyra
Brenthiinae: Brenthia
Epermeniidae
Epermeniidae: Epermenia ch.
Epermeniidae: Epermenia sin.
epermeniid sp.
Schreckensteiniidae
Schreckensteinia sp.1
Schreckensteinia sp.2
Millieriidae
Millieriidae: Millieria
Glyphipterigidae
+ Plutellidae
Glyphipteriginae: Glyphipterix
Glyphipteriginae: Lepidotarphius
Glyphipteriginae: Diploschizia
Acrolepiinae: Acrolepiopsis
Acrolepiinae: Digitivalva
Plutellidae: Doxophyrtis
Plutellidae: Proditrix
Orthoteliinae: Orthotelia
Plutellidae: Plutella
Ypsolophidae
Ypsolophidae: Ochsenheimeria
Ypsolophidae: Ypsolopha
Yponomeutidae.1
Yponomeutidae: Cedestis
Yponomeutidae: Yponomeuta
Yponomeutoidea
Yponomeutidae: Eucalantica
Yponomeutidae: Thecobathra
Heliodinidae
Heliodinidae: Aetole
Heliodinidae: Epicroesa
Yponomeut.2: Scythropiinae: Scythropia
Bedelliidae: Bedellia
Attevidae: Atteva
Lyonetiidae.1: Lyonetia
Praydidae: Prays
Yponomeutoidea
+Gracillarioidea
Gracillariidae
Oecophyllembiinae: Eumetriochroa
Gracillariinae: Spulerina
Phyllocnistinae: Phyllocnistis cit.
Phyllocnistinae: Phyllocnistis m.
Lithocolletinae: Cameraria
Lithocolletinae: Phyllonorycter
Lithocolletinae: Anartioses
Gracillariinae: Caloptilia
Gracillariinae: Callisto
Gracillariinae: Epicephala
Gracillariinae: Parectopa
Roeslerstammiidae
Roeslerstammiidae: Agriothera
Roeslerstammiidae: Roeslerstammia
Lyonetiidae.2: Cemiostominae: Leucoptera
Bucculatricidae
Bucculatricidae: Bucculatrix
0.2

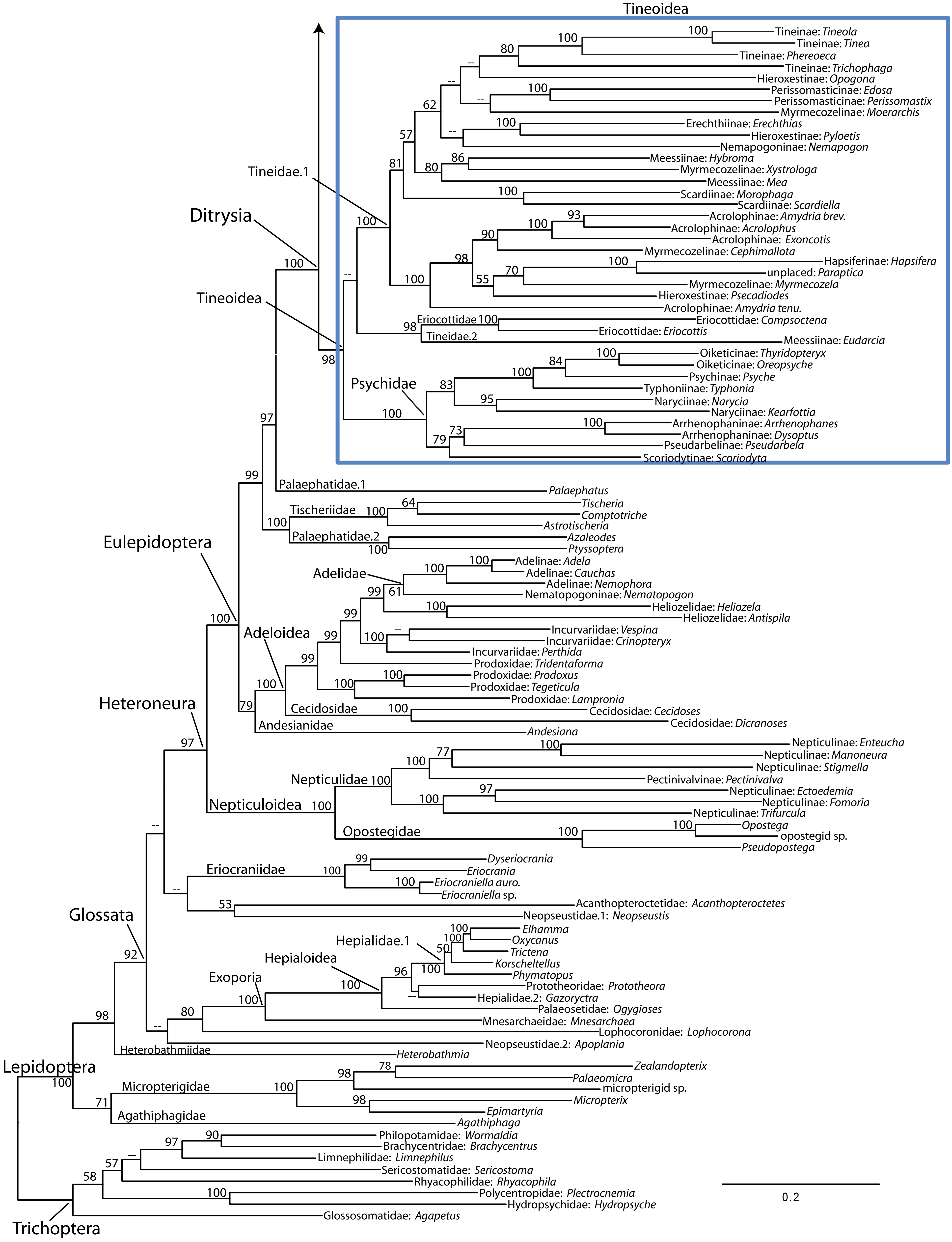

Tineoidea
Tineinae: Tineola
Tineinae: Tinea
Tineinae: Phereoeca
Tineinae: Trichophaga
Hieroxestinae: Opogona
Perissomasticinae: Edosa
Perissomasticinae: Perissomastix
Myrmecozelinae: Moerarchis
Erechthiinae: Erechthias
Hieroxestinae: Pyloetis
Nemapogoninae: Nemapogon
Meessiinae: Hybroma
Myrmecozelinae: Xystrologa
Meessiinae: Mea
Scardiinae: Morophaga
Scardiinae: Scardiella
Acrolophinae: Amydria brev.
Acrolophinae: Acrolophus
Acrolophinae: Exoncotis
Myrmecozelinae: Cephimallota
Hapsiferinae: Hapsifera
unplaced: Paraptica
Myrmecozelinae: Myrmecozela
Hieroxestinae: Psecadiodes
Acrolophinae: Amydria tenu.
Eriocottidae
Eriocottidae: Compsoctena
Eriocottidae: Eriocottis
Tineidae.2
Meessiinae: Eudarcia
Oiketicinae: Thyridopteryx
Oiketicinae: Oreopsyche
Psychinae: Psyche
Typhoniinae: Typhonia
Naryciinae: Narycia
Naryciinae: Kearfottia
Arrhenophaninae: Arrhenophanes
Arrhenophaninae: Dysoptus
Pseudarbelinae: Pseudarbela
Scoriodytinae: Scoriodyta
Tineidae.1
Ditrysia
Tineoidea
Psychidae
Palaephatidae.1
Palaephatus
Tischeriidae
Tischeria
Comptotriche
Astrotischeria
Palaephatidae.2
Azaleodes
Ptyssoptera
Eulepidoptera
Adelidae
Adelinae: Adela
Adelinae: Cauchas
Adelinae: Nemophora
Nematopogoninae: Nematopogon
Heliozelidae: Heliozela
Heliozelidae: Antispila
Adeloidea
Incurvariidae: Vespina
Incurvariidae: Crinopteryx
Incurvariidae: Perthida
Prodoxidae: Tridentaforma
Prodoxidae: Prodoxus
Prodoxidae: Tegeticula
Prodoxidae: Lampronia
Cecidosidae
Cecidosidae: Cecidoses
Cecidosidae: Dicranoses
Andesianidae
Andesiana
Heteroneura
Nepticulinae: Enteucha
Nepticulinae: Manoneura
Nepticulinae: Stigmella
Pectinivalvinae: Pectinivalva
Nepticulinae: Ectoedemia
Nepticulinae: Fomoria
Nepticulinae: Trifurcula
Nepticulidae
Nepticuloidea
Opostegidae
Opostega
opostegid sp.
Pseudopostega
Eriocraniidae
Dyseriocrania
Eriocrania
Eriocraniella auro.
Eriocraniella sp.
Acanthopteroctetidae: Acanthopteroctetes
Neopseustidae.1: Neopseustis
Glossata
Hepialidae.1
Elhamma
Oxycanus
Trictena
Korscheltellus
Phymatopus
Hepialoidea
Exoporia
Prototheoridae: Prototheora
Hepialidae.2: Gazoryctra
Palaeosetidae: Ogygioses
Mnesarchaeidae: Mnesarchaea
Lophocoronidae: Lophocorona
Neopseustidae.2: Apoplania
Heterobathmiidae
Heterobathmia
Lepidoptera
Zealandopterix
Palaeomicra
micropterigid sp.
Micropterigidae
Micropterix
Epimartyria
Agathiphagidae
Agathiphaga
Philopotamidae: Wormaldia
Brachycentridae: Brachycentrus
Limnephilidae: Limnephilus
Sericostomatidae: Sericostoma
Rhyacophilidae: Rhyacophila
Polycentropidae: Plectrocnemia
Hydropsychidae: Hydropsyche
Glossosomatidae: Agapetus
Trichoptera
0.2
