# Supplementary material for: A Large-Scale, Higher-Level, Molecular Phylogenetic Study of the Insect Order Lepidoptera (Moths and Butterflies)
Source: PLoS One. 2013 Mar 12;8(3):e58568. doi: 10.1371/journal.pone.0058568 (PMC3595289; doi:10.1371/journal.pone.0058568)
Supplement: Table S1 — Bootstrap results based on analysis of taxon-depleted nt123_degen1 data sets. (PDF) [file pone.0058568.s003.pdf]

Table S1. Bootstrap results based on analysis of taxon-depleted *nt123_degen1* data sets.[1]

**Node number**	**TAXONOMIC GROUP**	**483 taxa**	**453 taxa, no AC rogue**	**436 taxa, no RNR rogue**	**434 taxa, no RNR rogue, no Acan, no Neop: *Neopseustis***	**344 taxa, APODIT**	**314 taxa, APODIT, no AC rogue**	**133 taxa, MACRO**	**129 taxa, MACRO, no AC rogue**
	Bomb	97	92	98	98	95	92	98	94
	Lasi	95	100	99	98	99	100	97	100
	Bomb + Lasi	36	52	25	24	40	54	x	38
	Noct − *Doa*	100	100	100	100	100	100	100	100
24	Bomb + Lasi + Noct (- *Doa)*	14	x	x	x	15	x	x	x
	Bomb + Lasi + Cime	x	[no Cime]	[no Cime]	[no Cime]	x	[no Cime]	x	[no Cime]
	Bomb + Lasi + Drep:Drep	x	32	24	17	x	29	24	36
	Bomb + Lasi + Drep:Drep + Noct − *Doa*	x	32	x	19	x	23	20	24
	Mima + *Doa* + Geom:Sema + Drep:Epic	x	x	x	x	x	x	x	x
	Drep:Drep	70	100	89	83	74	100	67	100
	Geom:Geom	100	100	100	100	100	100	100	100
	Geom:Sema + Drep:Epic	68	72	74	76	70	61	66	59
	Geom:Geom + Geom:Sema + Drep:Epic	32	36	x	24	x	27	x	x
	Geom:Geom + Geom:Uran	x	x	x	x	51	x	41	x
	Geom + Drep:Epic	27	x	x	x	46	x	34	x
	Bomb + Lasi + Mima + *Doa* + Geom:Sema + Drep:Epic	x	x	x	x	x	x	x	x
22	Bomb + Lasi + Noct + Drep + Geom + Mima + *Doa* + Cime (= **MACRO**)	39	63 [no *Doa*, Cime]	79 [no *Doa*, Cime]	79	60	77 [no *Doa*, Cime]		
	**MACRO** − Drep:Drep	x	x	x	x	x	x	x	x
	Mima	100	100	100	100	100	100	100	100
	Mima + *Doa*	33	[no *Doa*]	[no *Doa*]	[no *Doa*]	44	[no *Doa*]	57	[no *Doa*]
	Pyra	74	74	70	80	71	77		
21	**MACRO** + Pyra	23	38	36	37	41	40		
	**MACRO** + Pyra + Hybl	31	27	39	26	34	24		
73	"butterflies"	83	80	86	82	82	79		
	Thyr	100	100	100	100	100	100		
	Gele	59	99	99	99	68	99		
15	**MACRO** + Pyra + Hybl + Copr + Eper + Thyr + Call + butterflies + Pter + Aluc (= **OBTECT)**	6	31	40	38	6	14		
19	**OBTECT** + Gele	14	65	58	57	16	34		
	Call + Hybl + Thry	x	x	x	x	x	x		

	Call + Hybl	x	x	x	x	x	x		
71	Eper + Copr − *Copromorpha*	35	69 (no *Copromorpha*)	76 (no *Copromorpha*)	73 (no *Copromorpha*)	37	54 (no *Copromorpha*)		
	Tort	100	100	100	100	100	100		
	Tort + Immo	x	44	32	x	x	x		
47	Zyga sensu stricto	96	100	100	100	100	100		
46	Zyga + Sesi + Coss	x	25 [no Zyga:Cycl or Zyga:Epip]	x	3	2 [no Zyga:Cycl or Zyga:Epip]	23 [no Zyga:Cycl or Zyga:Epip]		
	Sesi + Coss	x	67	x	x	x	64		
	Schr + Grac:Doug	37	[no Grac:Doug]	78	77	39	[no Grac:Doug]		
	Ditrysia − (Chor, Urod, Ypon, Grac, Tine)	13		x	x				
16	Ditrysia − (Urod, Ypon, Grac, Tine)	40		56	57				
15	Ditrysia − (Ypon, Grac, Tine)	98		99	99				
	Ypon + Grac	99		100	100				
	Tine	x		x	x				
14	Ditrysia − Tine (= **DnT**)	100		100	100				
	Tine:Tine (no *Eudarcia)* + Tine:Acro	100		100	100				
13	**DnT** + Tine:Tine (no *Eudarcia*) + Tine:Acro	64		57	55				
	Tine:Eriocot	100		100	100				
	Tine:Erio +Tine:*Eudarcia*	x		x	x				
12	**DnT** + Tine:Tine (no *Eudarcia*) + Tine:Acro + Tine:Eriocot	68		67	65				
	Tine:Psyc + Tine:Arrh +Tine:Eriocot + Tine:*Eudarcia*	x		x	x				
	Tine:Psyc + Tine:Arrh	100		100	100				
11	**DnT** + Tine − Tine:*Eudarcia*	87		90	94				
10	Ditrysia (= **DnT** + Tine)	100		100	100				
9	Ditrysia + Pala:*Palaeophatus*	92		89	89				
	Tisc + Pala (no Pala:*Palaeophatus)*	100		100	99				
8	Ditrysia + Tisc + Pala	99		99	99				
33	Adel	100		100	100				
32	Adel + Ande	67		66	68				
7	Ditrysia + Tisc + Pala + Adel + Ande (= **EULEP**)	100		100	100				
	Nept	100		100	100				
6	**EULEP** + Nept	99		100	100				

	Acan + Neop	35		76 [no Neop:*Apoplania*]	[no Acan or Neop]				
	Acan + Neop + Erio	x		45 [no Neop:*Apoplania*]	[no Acan or Neop]				
	**EULEP** + Nept + Acan + Neop + Erio	26		33 [no Neop:*Apoplania*]	52 [no Acan or Neop]				
28	Hepi + Mnes	100		100	100				
27	Hepi + Mnes + Loph	58		72	92				
4	**EULEP** + Nept + Acan + Neop + Hepi + Mnes + Loph	28		x	x				
	Erio	100		100	100				
3	**EULEP** + Nept + Acan + Neop + Hepi + Mnes + Loph + Erio (= **GLOS**)	95		100	100 [no Acan or Neop]				
2	**GLOS** + Heterob	99		99	99				
	Micr	100		100	100				
	Microp + Agat	70		77	76				
1	Lepidoptera	100		100	100				

[1] Bootstrap results in PAUP* are those shown under the "le = yes" option. "Node number" (column 1) refers to correspondingly numbered nodes in Figure 3. "Strong" bootstrap values, i.e., ≥80%, are highlighted in yellow. "Moderate" bootstrap values, i.e., 70-79%, are highlighted in green. Bootstrap results for taxa highlighted in blue are also present in Table 4. *x*, Not present in bootstrap table under le option, so value <50%; *ACrogue*, Adams-consensus rogue; *RNRrogue*, RNR rogue; *heterog*, heterogeneous taxa according to the boundaries in Figure C. *Bomb*, Bombycoidea; *Lasi*, Lasiocampidae; *Drep:Drep*, Drepanoidea:Drepanidae; *Geom:Sema*, Geometroidea:Sematuridae; *Drep:Epic*, Drepanoidea:Epicopeiidae; *Noct*, Noctuoidea; *Drep*, Drepanoidea; *Geom*, Geometroidea; *Mima*, Mimallonidae; *Cime*, Cimeliidae; *Geom:Geom*, Geometroidea:Geometridae; *Geom:Uran*, Geometroidea:Uraniidae; *MACRO*, Macroheterocera; *Doa*, Noctuoidea: *Doa* sp.; *Pyra*, Pyraloidea; *Hybl*, Hyblaeidae; *Gele*, Gelechioidea; *Copr*, Copromorphoidea; *Eper*, Epermeniidae; *Thyr*, Thyrididae; *Call*, Callidulidae; *"butterflies"*, Nymphalidae + Lycaenidae + Pieridae + Hedylidae + Hesperiidae + Papilionidae; *Pter*, Pterophoridae; *Aluc*, Alucitidae; *Gele*, Gelechioidea; OBTECT, Obtectomera; *Copromorpha*, *Copromorpha* sp.; *Tort*, Tortricoidea; *Immo*, Immoidea; *Zyga*, Zygaenoidea; *Sesi*, Sesioidea; *Coss*, Cossoidea; *Zyga:Cycl*, Zygaenoidea:Cyclotornidae; *Zyga:Epip*, Zygaenoidea:Epipyropidae; *Zyga sensu stricto*, Zygaenoidea − (Zyga:Cycl, Zyga:Epip); *Schr*, Schreckensteiniidae; *Grac:Doug*, Gracillarioidea:Douglasiidae; *Ditrysia*, Ditrysia (as defined in Figure S1); *Urod*, Urodidae; *Ypon*, Yponomeutoidea; *Grac*, Gracillarioidea; *Tine*, Tineoidea; *Tine:Tine*, Tineoidea:Tineidae; *Tine:Acro*, Tineoidea:Acrolophidae; *Tine:Eriocot*, Tineoidea:Eriocottidae; *DnT*, "Ditrysia no Tineoidea"; *Tine:Eudarcia*, Tineoidea:*Eudarcia* sp.; *Tine:Psyc*, Tineoidea:Psychidae; APODIT, Apoditrysia; *Adel*, Adeloidea; *Ande*, Andesianidae; *Eulep*, Eulepidoptera; *Acan*, Acanthopteroctetidae; *Neop*, Neopseustidae; *Erio*, Eriocraniidae; *Neop:Apoplania*, Neopseustidae:*Apoplania* sp.; *Tisc*, Tischeriidae; *Pala*, Palaephatidae; *Pala:Palaephatus*, Palaephatidae:*Palaephatus luteolus*; *Erio*, Eriocraniidae; *Hepi*, Hepialidae; *Mnes*, Mnesarchaeidae; *Loph*, Lophocoronidae; *Nept*, Nepticuloidea; *Agat*, Agathiphagidae; *GLOS*, Glossata; *Heterob*, Heterobathmiidae; *Micr*, Micropterigidae. *Lepidoptera*, Lepidoptera.
