# Supplementary material for: A Large-Scale, Higher-Level, Molecular Phylogenetic Study of the Insect Order Lepidoptera (Moths and Butterflies)
Source: PLoS One. 2013 Mar 12;8(3):e58568. doi: 10.1371/journal.pone.0058568 (PMC3595289; doi:10.1371/journal.pone.0058568)
Supplement: Table S2 — Bootstrap results based on analysis of taxon-depleted nt123 data sets. (PDF) [file pone.0058568.s004.pdf]

Table S2. Bootstrap results based on analysis of taxon-depleted *nt123* data sets.[1]

Node number	TAXONOMIC GROUP	483 taxa	455 taxa, no AC rogue	432 taxa, no RNR rogue	356 taxa, no RNR rogue, no heterog	344 taxa, APODIT	316 taxa, APODIT, no AC rogue	133 taxa, MACRO	129 taxa, MACRO, no AC rogue
	Bomb	98	99	99	99	99	99	98	94
	Lasi	99	100	100	100	100	100	100	100
	Bomb + Lasi	63	95	97	99	54	96	76	82
	Noct − *Doa*	100	100	100	100	100	100	100	100
24	Bomb + Lasi + Noct - *Doa*	x	x	x	23	x	x	x	x
	Bomb + Lasi + Cime	39	[no Cime]	[no Cime]	[no Cime]	57	[no Cime]	x	[no Cime]
	Bomb + Lasi + Drep:Drep	x	x	x	x	x	x	x	x
	Bomb + Lasi + Drep:Drep + Noct − *Doa*	x	x	x	x	x	x	x	x
	Mima + *Doa* + Geom:Sema + Drep:Epic	26	x	x	x	38	62	x	65 [no *Doa*]
	Drep:Drep	92	100	99	99	97	100	97	100
	Geom:Geom	100	100	100	100	100	100	100	100
	Geom:Sema + Drep:Epic	91	88	97	91	94	76	94	68
	Geom:Geom + Geom:Sema + Drep:Epic	x	x	x	x	x	x	x	x
	Geom:Geom + Geom:Uran	91	x	92	49	98	75	97	87
	Geom + Drep:Epic	x	x	x	40	x	x	x	x
	Bomb + Lasi + Mima + Doa + Geom:Sema + Drep:Epic	15	x	x	x	16	x	24 [+ Cime]	54
22	Bomb + Lasi + Noct + Drep + Geom + Mima + Doa + Cime (= **MACRO**)	55	36 [no *Doa*, Cime]	82 [no Cime]	88 [no Cime]	83	70 [no *Doa*, Cime]		
	**MACRO** − Drep Drep	x	x	x	x	x	27	x	61
	Mima	100	100	100	100	100	100	100	100
	Mima + *Doa*	71	[no *Doa*]	88	92	85	[no *Doa*]	85	[no *Doa*]
	Pyra	89	95	98	97	95	78		
21	MACRO + Pyra	37	23	58	68	58	47		
	**MACRO** + Pyra + Hybl	x	x	x	x	x	x		
73	"butterflies"	72	57	76	64	72	48		
	Thyr	100	100	100	100	100	100		
	Gele	52	29	68	72	52	54		
15	**MACRO** + Pyra + Hybl + Copr + Eper + Thyr + Call + "butterflies" + Pter + Aluc (= **OBTECT)**	x	x	x	x	x	x		
19	**OBTECT** + Gele	x	x	x	x	x	x		
	Call + Hybl + Thry	x	59	79	72	x	40		
	Call + Hybl	24	83	70	68	x	69		
71	Eper + Copr − *Copromorpha*	x	17 (no *Copromorpha*)	x	21 (no *Copromorpha*)	14	13 (no *Copromorpha*)		
	Tort	100	100	100	100	100	100		
	Tort + Immo	53	84	72	92	60	94		
47	Zyga sensu stricto	100	100	100	100	100	100		

46	Zyga + Sesi + Coss	2	96 [no Zyga:Cycl or Zyga:Epip]	32	41	x	95 [no Zyga:Cycl or Zyga:Epip]		
	Sesi + Coss	x	x	x	x	x	x		
	Schr + Grac:Doug	42	[no Grac:Doug]	[no Grac:Doug]	[no Grac:Doug]	x	[no Grac:Doug]		
	Ditrysia − (Chor, Urod, Ypon, Grac, Tine)	x		x	x				
16	Ditrysia − (Urod, Ypon, Grac, Tine)	x		x	x				
15	Ditrysia − (Ypon, Grac, Tine)	98		99	99				
	Ypon + Grac	97		99	99				
	Tine	97		100	62				
14	Ditrysia − Tine (= **DnT**)	100		99	100				
	Tine:Tine (no *Eudarcia*) + Tine:Acro	100		100	100				
13	**DnT** + Tine:Tine (no *Eudarcia*) + Tine:Acro	x		x	x				
	Tine:Eriocot	100		100	[1 taxon]				
	Tine:Eriocot + Tine:*Eudarcia*	98		99	[no Tine:*Eudarcia*]				
12	**DnT** + Tine:Tine (no *Eudarcia)* + Tine:Acro + Tine:Eriocot	x		x	x				
11	Tine:Psyc + Tine:Arrh +Tine:Eriocot + Tine:*Eudarcia*	53		61	95 [no Tine:*Eudarcia*]				
	Tine:Psyc + Tine:Arrh	100		100	100				
	**DnT** + Tine − Tine:*Eudarcia*	x		x	[no Tine:*Eudarcia*]				
10	Ditrysia	100		100	100				
9	Ditrysia + Pala:*Palaeophatus*	97		95	95				
	Tisc + Pala (no *Palaeophatus*)	100		99	97				
8	Ditrysia + Tisc + Pala	99		97	100				
33	Adel	100		100	100				
32	Adel + Ande	79		83	90				
7	Ditrysia + Tisc + Pala + Adel + Ande (= **EULEP**)	100		100	100				
	Nept	100		100	100				
6	**EULEP** + Nept	97		100	100				
	Acan + Neop	x		[no Neop]	[no Neop]				
	Acan + Neop + Erio	x		88 [no Neop]	70 [no Neop]				
	**EULEP** + Nept + Acan + Neop + Erio	x		88 [no Neop]	49 [no Neop]				
28	Hepi + Mnes	100		100	100				
27	Hepi + Mnes + Loph	80		100	[no Loph]				
4	**EULEP** + Nept + Acan + Neop + Hepi + Mnes + Loph	x		x	x				
	Erio	100		100	100				
3	**EULEP**+ Nept + Acan + Neop + Hepi + Mnes + Loph + Erio (= **GLOS)**	92		99	100				

2	**GLOS** + Heterob	98		97	96				
	Microp	100		100	100				
	Agat + Microp	71		72	81				
1	Lepidoptera	100		100	100				

[1] Bootstrap results in PAUP* are those shown under the "le = yes" option. "Node number" (column 1) refers to correspondingly numbered nodes in Figure 3. "Strong" bootstrap values, i.e., ≥80%, are highlighted in yellow. "Moderate" bootstrap values, i.e., 70-79%, are highlighted in green. Bootstrap results for taxa highlighted in blue are also present in Table 5. *x*, Not present in bootstrap table under le option, so value <50%; *ACrogue*, Adams-consensus rogue; *RNRrogue*, RNR rogue; *heterog*, heterogeneous taxa -- see Figure C. *Bomb*, Bombycoidea; *Lasi*, Lasiocampidae; *Drep:Drep*, Drepanoidea:Drepanidae; *Geom:Sema*, Geometroidea:Sematuridae; *Drep:Epic*, Drepanoidea:Epicopeiidae; *Noct*, Noctuoidea; *Drep*, Drepanoidea; *Geom*, Geometroidea; *Mima*, Mimallonidae; *Cime*, Cimeliidae; *Geom:Geom*, Geometroidea:Geometridae; *Geom:Uran*, Geometroidea:Uraniidae; *MACRO*, Macroheterocera; *Doa*, Noctuoidea: *Doa* sp.; *Pyra*, Pyraloidea; *Hybl*, Hyblaeidae; *Gele*, Gelechioidea; *Copr*, Copromorphoidea; *Eper*, Epermeniidae; *Thyr*, Thyrididae; *Call*, Callidulidae; *"butterflies"*, Nymphalidae + Lycaenidae + Pieridae + Hedylidae + Hesperiidae + Papilionidae; *Pter*, Pterophoridae; *Aluc*, Alucitidae; *Gele*, Gelechioidea; OBTECT, Obtectomera; *Copromorpha*, *Copromorpha* sp.; *Tort*, Tortricoidea; *Immo*, Immoidea; *Zyga*, Zygaenoidea; *Sesi*, Sesioidea; *Coss*, Cossoidea; *Zyga:Cycl*, Zygaenoidea:Cyclotornidae; *Zyga:Epip*, Zygaenoidea:Epipyropidae; *Zyga sensu stricto*, Zygaenoidea − (Zyga:Cycl, Zyga:Epip); *Schr*, Schreckensteiniidae; *Grac:Doug*, Gracillarioidea:Douglasiidae; *Ditrysia*, Ditrysia (as defined in Figure S1); *Urod*, Urodidae; *Ypon*, Yponomeutoidea; *Grac*, Gracillarioidea; *Tine*, Tineoidea; *Tine:Tine*, Tineoidea:Tineidae; *Tine:Acro*, Tineoidea:Acrolophidae; *Tine:Eriocot*, Tineoidea:Eriocottidae; *DnT*, "Ditrysia no Tineoidea"; *Tine:Eudarcia*, Tineoidea:*Eudarcia* sp.; *Tine:Psyc*, Tineoidea:Psychidae; APODIT, Apoditrysia; *Adel*, Adeloidea; *Ande*, Andesianidae; *Eulep*, Eulepidoptera; *Acan*, Acanthopteroctetidae; *Neop*, Neopseustidae; *Erio*, Eriocraniidae; *Tisc*, Tischeriidae; *Pala*, Palaephatidae; *Pala:Palaephatus*, Palaephatidae:*Palaephatus luteolus*; *Erio*, Eriocraniidae; *Hepi*, Hepialidae; *Mnes*, Mnesarchaeidae; *Loph*, Lophocoronidae; *Nept*, Nepticuloidea; *Agat*, Agathiphagidae; *GLOS*, Glossata; *Heterob*, Heterobathmiidae; *Micr*, Micropterigidae. *Lepidoptera*, Lepidoptera.
