# Supplementary material for: A Large-Scale, Higher-Level, Molecular Phylogenetic Study of the Insect Order Lepidoptera (Moths and Butterflies)
Source: PLoS One. 2013 Mar 12;8(3):e58568. doi: 10.1371/journal.pone.0058568 (PMC3595289; doi:10.1371/journal.pone.0058568)
Supplement: Table S4 — Synopsis of genes sequenced. (PDF) [file pone.0058568.s006.pdf]

Table S4. Synopsis of genes sequenced.*

PCR amplicon name	Gene name	Fragment length(s)
CAD	(multiple enzymes for pyrimidine biosynthesis)	2865
DDC	dopa decarboxylase	1281
enolase	enolase	1134
period	period	873
wg	wingless	390
109fin	gelsolin	573
192fin	glutamyl- and prolyl-tRNA synthetase	402
197fin	triophosphate isomerase	444
265fin	histidyl-tRNA synthetase	447
268fin	AMP deaminase	768
3007fin	glucose phosphate dehydrogenase	621
3017fin	tetrahydrofolate synthase	594
3070fin	alanyl-tRNA synthetase	699
262 fin	(proteasome subunit)	486
40fin	phosphogluconate dehydrogenase	750
42fin	(putative GTP-binding protein)	840
8028fin	nucleolar cysteine-rich protein	324
8091fin	glucose phosphate isomerase	666
acc	acetyl-coA carboxylase	501

* These 19 genes have been used in previous studies, e.g., see reference [17] for the first five genes and reference [22] for the others. CAD, DDC, enolase, and period have multiple amplicons. All others are single amplicons. Fragment lengths do not include the ambiguously aligned characters that were excluded from the final data sets. The lengths of some gene fragments differ slightly from previous studies because the current data set was realigned after the substantial increase in total number and diversity of taxa included.
