# Supplementary material for: A Large-Scale, Higher-Level, Molecular Phylogenetic Study of the Insect Order Lepidoptera (Moths and Butterflies)
Source: PLoS One. 2013 Mar 12;8(3):e58568. doi: 10.1371/journal.pone.0058568 (PMC3595289; doi:10.1371/journal.pone.0058568)
Supplement: Table S5 — Absolute number of unambiguous nucleotides (bp) per gene in each taxon, plus summary statistics. (PDF) [file pone.0058568.s007.pdf]

Table S5. Absolute number of unambiguous nucleotides (bp) per gene in each taxon.*

GENE NAME ➔	40fin	42fin	109fin	192fin	197fin	262fin	265fin	268fin	3007fin	3017fin	3070fin	8028fin	8091fin	ACC	CAD	DDC	Enolase	Period	WG
**AK142_SESI_BRAC**	0	0	0	0	0	0	0	0	0	0	0	0	0	0	2310	1050	1134	0	390
**AK154_NEPT_OPOS**	747	0	573	402	444	486	447	768	621	0	699	0	666	501	1569	708	0	0	390
**Aaeq_BOMB_EUPT_EUPT**	750	840	573	402	444	486	0	768	621	594	699	324	666	501	2865	1257	1134	870	390
**Abre_TINE_ACRO**	0	0	573	0	0	0	447	768	621	0	0	0	0	501	2865	0	1134	0	0
**Acal_PYRA_PYRA_EPIP**	750	0	0	0	444	486	0	768	621	0	699	324	666	501	2865	1281	1134	870	390
**Acer_GELE_PELE**	750	840	573	402	444	486	447	768	621	594	699	324	666	501	2865	1281	1134	525	390
**Acly_PAPI_NYMP_APAT**	750	840	573	402	444	486	447	768	621	594	0	324	0	501	2681	939	1134	864	390
**Acoa_ZYGA_DALC**	750	840	573	0	444	486	447	768	621	594	699	324	665	501	2865	1281	1134	0	390
**Acro_GEOM_URAN_MICR**	0	0	0	0	0	486	0	768	621	0	0	324	0	0	2032	1281	1134	873	390
**Adam_PTER_PTER_AGDI**	750	840	573	402	444	486	447	768	621	567	0	324	666	501	2046	1050	1134	870	390
**Aesp_TORT_TORT_TORT**	708	0	573	0	444	486	447	768	621	594	699	324	666	501	2865	456	1134	867	0
**Aetr_YPON_HELI**	750	840	570	402	444	486	447	0	621	594	699	324	666	501	2865	708	1134	873	390
**Aeuro_BOMB_BRAH**	750	840	573	402	444	486	447	768	621	594	699	324	666	501	2865	1281	1134	822	390
**Afor_NOCT_ARCT_SYN**	750	840	573	402	444	486	447	0	621	594	0	324	666	501	2848	1050	1134	873	390
**Agdi_PTER_PTER_MACR**	750	840	573	0	444	486	447	768	0	594	699	324	666	500	2865	0	1134	873	390
**Agel_GRAC_ROES**	750	840	558	0	444	486	447	768	621	594	699	324	666	501	2865	0	1134	0	0
**Agip_NOCT_NOCT_NOCT**	750	840	573	402	444	486	447	768	621	594	0	324	666	501	2865	1281	1134	873	390
**Agpt_GELE_ELAC_DEPR**	750	0	573	402	444	486	442	768	621	594	699	324	666	501	2865	0	1134	873	390
**Agus_TRICHOPTERA**	0	0	0	0	0	0	0	0	0	0	0	0	0	0	2454	708	1134	852	0
**Ahpt_GEOM_GEOM_ALSO**	750	0	573	0	444	486	447	768	621	594	699	324	666	0	2865	1281	1134	873	387
**Ahss_COSS_COSS**	750	840	573	0	444	486	447	0	621	594	699	0	666	501	2670	1281	1134	856	390
**Aios_ZYGA_AIDI**	750	0	573	0	0	486	447	768	621	594	699	324	0	501	2043	708	750	873	387
**Alsp_ALUC_ALUC**	750	840	573	402	444	486	447	768	618	0	699	324	666	501	2169	456	1134	873	387
**Ambe_PYRA_PYRA_PHYC**	750	840	573	0	444	486	447	768	621	594	699	324	666	0	2169	1281	1134	873	390
**Amca_SESI_CAST**	750	840	573	0	444	486	447	0	621	0	699	324	666	501	2865	939	642	873	387
**Amod_GELE_AUTO_AUTO**	750	840	573	402	444	486	447	768	621	574	699	324	666	501	2865	993	1134	873	390
**Amst_GELE_GELE_ANOM**	750	840	573	402	444	486	447	768	621	594	699	324	666	501	2865	1281	1134	513	390
**Anab_GRAC_GRAC**	0	0	573	0	0	0	0	0	621	0	0	0	0	495	2865	708	1134	0	0
**Ancy_TORT_TORT_OLET**	0	840	573	0	444	486	0	768	621	594	699	324	0	501	2865	456	1134	870	0
**Anfa_CHOR_CHOR_CHOR**	750	840	573	0	444	486	447	768	621	0	699	324	666	501	2865	939	1134	498	390
**Anla_ANDE_ANDE**	750	840	555	0	444	486	447	768	621	0	699	0	666	501	2862	0	1134	867	387
**Anma_PTER_PTER_PTER**	750	840	573	0	444	486	447	0	621	594	0	324	666	501	2310	1281	1134	0	390
**Anne_TORT_TORT_TORT**	750	0	573	0	0	486	0	0	621	594	699	0	666	501	2036	1050	1134	867	390
**Anph_ZYGA_LACT**	0	0	0	0	0	0	0	0	0	0	0	0	0	0	2865	1050	1134	513	390
**Apar_GEOM_GEOM_ARCH**	0	0	573	402	0	486	0	768	621	594	0	324	666	500	2311	1281	1134	0	390
**Apauk_BOMB_SATU_SATU**	750	840	573	402	444	486	447	768	621	594	699	324	666	501	0	1281	0	819	390
**Aphy_PYRA_CRAM_ODON**	750	840	573	402	444	486	447	768	621	594	699	324	666	501	0	0	0	0	0
**Aquen_AGAT_AGAT**	750	840	573	402	444	486	447	768	621	594	699	324	666	501	2862	0	1134	0	0
**Arca_TINE_ACRO**	750	0	573	402	444	486	447	768	621	571	0	324	0	501	0	1281	1134	0	390
**Aren_GELE_ELAC_STEN**	0	0	0	0	0	486	0	0	621	594	699	0	0	0	2865	456	1134	0	390
**Arga_TORT_TORT_TORT**	0	840	573	402	444	486	447	743	621	0	699	324	666	501	2865	1281	1134	861	387
**Arot_GELE_GELE_GELE**	0	840	573	402	444	486	447	768	621	593	0	324	663	501	2865	939	1134	873	390
**Arrp_TINE_ARRH**	0	840	573	0	444	0	447	768	621	0	699	0	648	501	2169	456	1134	0	0

**Asap_YPON_ACRO**	750	840	573	402	444	486	447	768	621	594	699	324	666	0	2865	0	1134	849	0
**Asem_GELE_ELAC_AEOL**	750	840	573	402	444	486	447	0	621	594	699	324	666	501	2865	1281	1134	0	390
**Ator_BOMB_APAT**	750	840	573	402	444	486	447	768	621	594	699	324	666	501	2865	1281	0	873	390
**Atpu2_YPON_YPON**	750	840	573	402	444	486	447	0	621	594	699	324	666	501	2169	939	1134	502	389
**Atr5_INCU_ADEL_ADEL**	750	840	573	402	444	486	447	767	0	594	699	0	666	501	2862	651	1134	0	0
**Atth_TISC_TISC**	0	0	573	402	444	486	447	768	621	594	0	0	666	501	2862	0	1131	0	0
**Audi_TORT_TORT_CHLI**	750	840	573	402	444	486	447	768	621	594	699	324	666	501	2733	1281	1134	0	387
**Aun2_ACAN_ACAN**	750	840	573	402	444	486	447	768	0	594	699	324	666	501	2862	1281	1134	864	0
**Avld_NEOP_NEOP**	0	0	0	0	0	0	0	0	0	0	0	0	0	0	2862	708	0	0	0
**Avog_INCU_HELI**	750	840	573	402	444	486	447	768	621	594	699	309	666	501	2862	708	1134	0	378
**Axsp_CIME_CIME**	750	0	573	402	444	486	447	768	621	594	699	324	666	501	2865	1257	1134	873	390
**Ayte_TINE_ACRO_closetoPtcu**	750	0	573	0	444	0	447	768	0	594	699	324	0	501	2865	708	1134	873	0
**Ayth_GELE_GELE**	0	840	573	0	444	486	447	768	621	594	0	324	666	501	2865	1050	0	0	390
**Azal_PALA_PALA**	750	840	573	0	444	486	447	768	621	594	699	0	666	501	2862	708	1134	0	390
**Ball_GELE_ELAC_DEPR**	750	840	573	402	444	486	447	768	621	594	699	324	666	501	0	1281	1134	0	390
**Basp_TORT_TORT_OLET**	750	840	573	402	444	486	447	768	621	594	699	324	666	498	2862	1050	1134	873	390
**Bbet_GEOM_GEOM_ENNO**	747	0	573	402	0	486	447	768	621	594	699	324	0	501	2865	456	1134	810	387
**Bcle_IMMO_IMMI**	0	840	0	402	444	486	447	768	621	594	699	324	0	0	2169	0	1134	0	390
**Bedg2_MIMA_MIMA**	0	0	573	0	0	0	447	768	621	0	0	0	0	501	2865	1050	1134	0	0
**Bmor1_BOMB_BOMB_BOMB**	750	840	573	402	444	486	447	768	621	594	699	324	666	501	2865	1281	1134	822	390
**Bni2_TRICHOPTERA**	750	840	541	0	444	486	447	768	621	591	699	324	666	501	2862	708	1134	0	0
**Bogo_TORT_TORT_TORT**	750	0	0	0	444	486	447	0	621	0	699	324	0	501	2169	1050	1134	858	0
**Bren_CHOR_CHOR_BREN**	0	840	0	402	444	486	447	768	621	594	699	0	666	501	2865	1050	1134	873	390
**Bsex_NOCT_NOLI_BLEN**	750	840	573	402	0	486	447	0	621	594	699	324	666	501	2844	1281	1134	873	390
**Bsmu_YPON_BEDE**	750	840	573	402	444	485	447	768	621	594	699	321	666	501	2858	939	1134	867	390
**Bucc_GRAC_BUCC**	750	840	0	402	444	486	447	0	621	594	0	324	660	501	2865	1050	1134	0	390
**CR19_GELE_checkID**	0	0	573	0	0	0	447	768	621	0	0	0	0	501	2865	1050	1134	0	0
**Cafd_COPR_CARP**	750	840	573	402	444	486	447	0	621	594	699	0	666	501	2865	939	1134	0	387
**Caga_GRAC_GRAC_LITH**	750	840	573	402	444	486	447	768	621	594	699	324	666	501	2865	0	1134	0	390
**Calfa_TORT_TORT_CNEP**	540	0	573	402	444	486	447	768	621	0	699	324	666	501	2865	1026	1134	0	0
**Caor_PYRA_CRAM_CRAM**	0	0	573	402	444	486	447	768	621	594	699	0	666	501	2865	1281	1134	867	390
**Caul_NOCT_NOCT_CATO**	750	840	573	402	444	486	447	768	621	594	0	324	666	501	2367	1281	1134	873	390
**Cbes_NOCT_NOTO_DUDU**	750	840	573	402	444	486	447	768	621	594	699	324	0	501	2865	1281	1134	873	390
**Cbim_GRAC_GRAC_GRAC**	750	840	552	402	444	486	447	768	621	594	699	324	666	501	2865	1281	642	0	388
**Ccol_BOMB_ANTH**	750	0	573	402	444	486	447	768	621	594	699	324	666	501	2865	1281	1134	822	390
**Cdel_GRAC_GRAC_GRAC**	750	840	573	402	444	486	447	768	621	582	699	324	666	501	2865	1050	1134	864	390
**Cecid_INCU_CECI**	750	840	573	0	444	486	447	768	621	0	699	0	666	501	2862	570	1134	0	390
**Cera_TORT_TORT_TORT**	750	840	573	0	444	486	447	768	621	0	699	324	666	501	0	939	1134	870	0
**Cet_HESP_HESP_HESP**	0	837	567	402	0	486	447	0	620	0	0	324	0	501	2865	699	1134	0	390
**Ceur_PAPI_PIER_COLI**	750	0	573	402	444	486	447	768	621	594	699	324	666	501	2842	1050	1134	873	390
**Cfu2_NOCT_ARCT_ARCT**	750	840	573	402	444	486	0	768	621	594	699	324	666	501	2865	1281	1134	873	390
**Cill_GELE_ELAC_ELAC**	0	840	573	0	0	486	0	768	621	594	0	0	666	501	2865	0	1134	0	390
**Cler_TORT_TORT_TORT**	0	840	573	0	444	486	447	768	621	594	699	324	0	501	2865	1050	642	732	390
**Clph_GELE_COLE_COLE**	0	839	573	402	444	486	447	768	621	594	699	0	666	501	2865	939	1134	870	390
**Cmar_GEOM_GEOM_GEOM**	750	840	573	402	444	486	447	768	621	594	699	324	666	501	2865	1281	1134	873	390
**Cmtn_LASI_LASI_CHIO**	750	0	573	402	444	486	447	768	621	594	699	324	666	501	2865	1281	1134	513	390

**Cole_GELE_COSM_BIAS**	0	840	0	402	444	486	0	768	621	594	699	0	666	501	2865	1050	642	873	390
**Comp_TINE_ERIO_COMP**	750	840	573	0	444	486	447	768	621	594	699	324	666	501	2310	0	1134	0	390
**Cooh_GEOM_SEMA_SEMA**	750	0	573	402	0	0	447	0	621	0	0	324	0	0	2157	1278	750	873	0
**Copro_COPR_COPR**	0	840	573	402	444	486	447	768	621	594	699	324	666	501	2865	0	1134	873	390
**Cosa_TINE_TINE_MYRM**	0	0	0	0	0	0	0	0	0	0	0	0	0	0	2658	1050	1134	870	390
**Cosla_COSS_COSS_COSS**	0	0	0	0	0	0	0	0	0	0	0	0	0	0	2865	939	1134	873	390
**Cosm_GELE_COSM_COSM**	0	0	573	0	444	486	447	768	621	594	699	324	0	501	2157	1050	1134	867	390
**Coss_COSS_COSS_COSS**	750	840	573	402	444	486	447	0	621	589	699	324	666	501	2865	1281	1134	873	390
**Coste_PYRA_CRAM_MIDI**	750	840	570	0	444	486	447	0	621	594	699	324	666	501	2865	0	1134	870	390
**Cotan_BOMB_EUPT**	750	840	573	402	444	486	0	768	621	588	699	324	666	0	2865	1281	1134	513	390
**Cper_GEOM_GEOM_ENNO**	0	840	573	0	444	486	447	768	621	594	0	324	666	501	2842	1050	1134	852	0
**Cpo_TORT_TORT_OLET**	750	840	573	402	444	486	447	768	621	594	699	324	666	501	2644	1281	1134	873	390
**Cpua_GELE_GELE_GELE**	0	0	573	0	0	0	447	0	621	0	0	0	0	501	1569	1050	1134	0	0
**Cram_HESP_HESP_COEL**	750	0	0	0	444	0	447	0	621	0	699	0	666	501	2865	456	0	0	390
**Crin_INCU_CRIN**	0	840	573	402	444	486	447	768	621	594	699	324	0	501	2169	939	1134	0	390
**Cros_TORT_TORT_TORT**	750	840	573	402	444	486	447	768	621	0	699	324	666	501	2865	939	1134	867	384
**Csi_INCU_ADEL_ADEL**	750	840	573	402	444	486	447	768	621	594	699	0	666	501	2862	708	642	522	0
**Cucr_COSS_COSS_COSS**	750	840	573	402	444	486	447	768	621	594	699	324	666	501	2865	1281	1134	513	390
**Cure_PAPI_LYCA_CURE**	750	840	573	402	444	486	447	768	621	594	0	324	666	501	2865	708	1134	855	390
**Cuvx_NOCT_NOCT_CUCU**	750	840	573	402	444	486	447	768	621	594	0	324	666	501	2865	1050	1134	873	390
**Cvan_LASI_LASI_CHON**	750	840	570	402	444	486	447	768	621	594	699	324	666	501	2865	1050	1134	0	390
**Cvdv_COSS_COSS_COSS**	0	840	573	402	444	486	447	0	621	0	699	324	666	501	2169	456	1134	0	390
**Cycs3_HESP_HESP_PYRG**	0	0	0	0	444	0	0	0	621	0	699	0	0	0	2169	456	1134	0	390
**Cyna_ZYGA_CYCL**	0	0	0	0	0	0	0	0	0	0	0	0	0	0	2865	1050	1134	858	390
**Cysp_ZYGA_CYCL**	750	840	573	402	444	486	447	768	621	594	699	324	666	501	2865	1281	642	858	390
**Cysu_DREP_DREP_CYCL**	750	0	573	402	444	486	0	768	621	590	699	324	0	499	2865	1281	1134	873	390
**Cytr_GEOM_GEOM_STER**	750	840	573	402	444	485	0	768	621	594	699	324	666	501	2865	1281	1134	873	0
**Dals_MIMA_MIMA**	750	840	573	402	444	486	447	768	621	594	699	324	666	501	2845	1257	1134	825	390
**Damp_PAPI_PIER_DISM**	0	0	0	0	444	486	447	768	621	594	699	0	666	0	2169	1281	1134	873	390
**Darcu_DREP_DREP_DREP**	750	840	573	0	444	486	447	768	621	594	0	324	666	0	2865	1050	1134	0	390
**Dasa_TORT_TORT_TORT**	750	0	573	402	444	486	0	0	621	0	699	324	666	501	2865	0	1134	0	0
**Dawi_BOMB_BRAH**	750	840	573	402	444	486	447	768	621	594	699	324	666	501	2865	1050	1134	870	390
**Dbil_TINE_ARRH**	750	0	573	0	444	486	447	0	621	0	699	0	666	501	1569	708	1134	498	0
**Dcap_INCU_CECI**	0	0	573	0	444	0	447	768	0	0	699	0	0	501	2862	0	642	0	0
**Deoy_GELE_DEOC_DEOC**	750	840	573	402	444	486	447	768	621	594	699	324	666	501	2865	1050	1134	0	390
**Dfag_GELE_CHIM**	0	0	573	402	0	486	447	0	621	0	699	0	0	501	1569	0	1134	513	390
**Dgr2_ERIO_ERIO**	750	840	573	0	444	486	447	768	621	585	699	0	666	501	2862	1050	1134	0	0
**Dhem_YPON_ACRO**	750	840	573	402	444	486	447	768	621	593	699	324	666	501	2865	456	1134	513	0
**Dhyd_YPON_PLUT**	0	0	573	402	0	486	447	768	621	0	0	0	0	501	2865	456	0	0	390
**Dich_GEOM_GEOM_DESM**	750	837	573	402	444	486	447	768	621	594	699	324	666	501	2865	1281	1134	873	390
**Dimp_YPON_GLYP**	750	840	573	402	444	486	447	0	621	594	699	324	663	501	2796	708	1134	846	0
**Ding2_ZYGA_DALC**	750	840	573	0	444	486	447	768	621	594	699	324	666	501	2434	1281	1134	0	390
**Disc_NOCT_OENO**	750	0	573	402	444	486	447	768	621	594	699	324	666	501	2865	1281	1134	873	390
**Dlec_GEOM_GEOM_OENO**	750	840	573	402	444	486	446	768	621	594	699	324	666	501	2865	1281	1134	873	0
**Dmet_PYRA_CRAM_GLAP**	750	840	573	0	444	486	447	768	621	594	699	324	666	501	2865	1281	1134	870	390
**Doa_NOCT_DOID**	0	840	573	0	444	486	447	768	621	579	699	324	666	501	2865	1281	1134	873	390

Dpd_GELE_ELAC_DEUT	750	0	573	402	441	486	447	768	621	591	699	324	666	501	2865	1050	1134	873	390
Dpe2_NOCT_NOTO_PHAL	750	840	573	402	0	486	447	768	621	594	699	324	666	501	2865	1281	1134	873	390
Dplex_PAPI_NYMP_DANA	0	0	573	0	0	485	0	768	621	0	699	0	0	501	1569	456	0	0	390
Dpunc_GELE_GELE_DICH	0	840	555	402	444	486	447	768	621	594	699	324	666	501	2865	1050	1134	870	390
Dsos_PAPI_NYMP_BIBL	750	0	0	402	444	0	0	0	0	0	699	0	660	0	2865	1050	1134	0	390
Dsp_ZYGA_DALC	750	840	573	0	444	486	447	768	621	594	699	324	665	501	2865	801	1134	807	390
Dudg_COSS_DUDG	750	0	0	0	0	0	447	0	621	0	699	0	0	501	1110	633	0	873	390
Dyso_THYR_THYR_THYR	0	0	0	0	0	485	447	0	621	0	0	0	0	501	2865	939	642	513	390
Eate_HEPI_HEPI	750	840	573	402	441	486	447	0	621	594	699	0	666	0	2862	0	1134	0	0
Ebdt_GEOM_GEOM_LARE	0	0	573	0	0	0	447	768	621	0	0	0	0	501	2865	708	1134	0	0
Edel3_ZYGA_LIMA_LIMA	750	840	573	402	444	486	447	768	621	594	699	324	666	501	2865	1281	1134	852	390
Edos_TINE_TINE_PERI	750	840	573	402	444	486	447	768	621	594	0	324	666	501	2865	939	1134	873	390
Edvs3_MIMA_MIMA	0	0	573	0	444	0	447	0	0	0	699	324	0	0	2169	1026	0	0	390
Eeny_COSS_COSS_ZEUZ	750	840	573	402	444	486	447	768	621	576	699	324	666	0	2865	1281	0	513	390
Eeu_GELE_ELAC_ETHM	750	840	573	402	444	486	441	768	621	594	699	324	666	501	2865	1281	1134	873	390
Ehai_DREP_EPIC	750	824	573	402	444	486	447	768	621	0	699	324	666	501	2865	1281	1134	873	390
Ehdr_GRAC_GRAC	750	840	0	402	444	486	447	768	621	594	699	324	666	501	2865	708	1134	738	390
Eheb_TORT_TORT_OLET	0	0	0	0	0	486	447	0	0	594	699	324	666	501	1992	456	1134	0	390
Ejzn_MIMA_MIMA	750	0	573	402	0	486	447	0	621	594	699	324	0	501	2865	1281	1134	0	390
Emet_YPON_HELI	750	0	570	402	444	486	447	768	621	594	699	324	0	501	2865	0	1134	501	390
Enac_NEPT_NEPT_NEPT	750	840	573	396	444	486	447	768	621	0	699	0	666	501	2859	1050	1134	858	390
Enaw_ZYGA_EPIP_EPIP	0	840	573	402	444	486	447	768	621	594	699	324	666	501	2865	1281	0	0	390
Epca_GELE_ELAC_HYPE	0	840	573	402	444	486	447	768	621	594	699	0	666	501	0	1281	1134	0	0
Epchh_EPER_EPER	750	840	573	402	444	486	447	768	621	594	699	324	666	501	2865	1281	1134	0	390
Epic_GRAC_GRAC_GRAC	750	0	558	402	0	486	447	768	0	594	699	0	0	501	2865	0	1134	0	390
Epmn_EPER_EPER	0	0	0	0	0	0	0	0	0	0	0	0	0	0	1569	456	0	0	387
Epo2_NEPT_NEPT_NEPT	750	840	573	396	444	483	447	768	621	594	699	324	666	501	2862	0	1134	0	390
Eppd_MICR_MICR	0	0	0	0	0	0	0	0	0	0	0	0	0	0	2853	651	0	0	0
Erau_ERIO_ERIO	750	840	573	0	444	486	447	768	621	0	699	0	666	501	2862	0	1134	0	0
Erof_NOCT_NOLI_EARI	0	840	573	402	444	486	447	768	621	594	699	324	0	501	2844	1281	1134	873	390
Ersn_TINE_ERIO	0	0	573	0	0	0	447	0	621	0	0	0	0	0	2310	651	1134	0	0
Esal_NOCT_NOCT_CALP	750	840	573	402	444	486	447	0	621	594	699	324	666	501	2853	1281	1134	0	390
Ese2_ERIO_ERIO	750	0	573	402	444	486	447	768	621	0	699	0	666	501	2862	708	1134	861	0
Esji_EPER_EPER	0	0	573	0	0	0	0	0	621	0	0	0	0	501	2865	456	1134	0	0
Esp2_ZYGA_EPIP	0	840	573	0	444	486	447	768	621	594	699	324	666	501	2169	0	0	0	390
Esub_PYRA_CRAM_EVER	750	840	573	402	444	486	447	768	621	594	699	324	666	0	2865	1281	1134	858	390
Etbk_TORT_TORT_TORT	0	840	573	0	444	486	447	0	0	0	699	324	666	501	2865	456	1134	481	0
Eten_GELE_ELAC_ELAC	0	0	573	402	444	486	447	0	621	547	699	0	642	501	2865	0	1134	0	390
Etgf_GELE_GELE_DICH	0	0	0	0	0	0	0	0	0	0	0	0	0	0	2865	1281	1134	0	390
Etsp_ZYGA_ZYGA_CHAL	750	840	573	0	444	486	447	768	621	594	699	324	666	501	2865	0	638	0	390
Etyr_TORT_TORT_OLET	0	0	0	0	0	0	0	0	0	0	0	0	0	0	2865	1050	1134	873	390
Euac_GEOM_GEOM_LARE	0	0	573	0	444	486	0	0	621	594	699	324	666	501	1305	699	0	867	390
Eucm_TORT_TORT_OLET	0	840	573	402	444	486	447	0	621	594	699	324	666	501	2865	1050	1134	873	0
Euds_TINE_TINE_MEES	750	840	573	402	444	486	447	0	621	594	699	324	666	501	2865	1281	0	0	390
EurytMibr_PAPI_PAPI_PAPI	750	0	573	0	0	486	447	0	621	594	699	324	666	501	2169	1050	1089	0	390
Eusp_TORT_TORT_OLET	0	840	573	402	444	486	0	768	621	594	699	324	666	501	2862	1050	1134	873	390

Eutr_TORT_TORT_TORT	750	840	573	402	444	486	447	0	621	594	699	324	666	501	2865	708	1134	0	390
Eversicol_BOMB_ENDR	750	0	573	402	444	486	447	768	621	594	699	324	0	501	2169	1281	1117	813	390
Exum_TINE_ACRO	0	0	573	0	0	0	447	768	621	0	0	0	0	501	2865	1050	1134	0	0
Ezeb_TINE_TINE_EREC	0	0	573	0	0	486	446	768	614	594	0	324	0	501	2865	708	1134	873	390
Fbrn_GELE_ELAC_DEPR	750	840	573	0	441	486	447	0	621	594	0	324	666	501	2865	708	1134	867	390
Fci2_NOCT_NOTO_NOTO	750	840	573	402	444	485	447	768	621	594	699	324	666	501	2865	1050	1134	867	390
Fhyp_NEPT_NEPT_NEPT	0	0	573	0	0	0	447	768	621	0	0	0	0	500	2862	708	1134	0	0
Gaeg_PYRA_PYRA_PYRA	750	0	573	402	444	486	0	768	621	585	699	324	0	501	2865	1281	1134	873	390
Gazm_HEPI_HEPI	0	840	0	0	441	486	0	768	621	0	0	0	666	501	0	0	1134	0	0
Gdel_TORT_TORT_OLET	750	0	573	402	444	486	447	0	621	594	699	324	666	501	2865	1050	1134	873	390
Gilt_THYR_THYR_THYR	750	0	573	402	444	485	447	768	621	594	0	324	0	501	2865	1281	1134	864	390
Glpx_YPON_GLYP_GLYP	750	0	573	402	444	486	447	0	621	594	699	0	0	501	2865	1050	0	0	0
Gmuc_COSS_COSS_HYPO	750	0	573	402	444	486	447	768	621	588	699	324	666	501	2865	1281	0	873	390
Gprot2_GELE_AUTO_SYMM	750	840	573	402	444	486	447	768	621	594	699	324	666	501	2658	1050	1134	513	390
Gsep_NOCT_NOTO_NOTO	750	840	572	402	444	486	447	768	618	594	0	324	666	501	2865	1281	1134	870	390
Gvii_CALL_CALL_GRIV	750	0	558	0	0	486	447	0	621	0	699	324	666	501	2865	1281	0	0	390
Gyrt_NOCT_NOCTquad_STIC	750	840	573	402	444	486	447	0	621	594	699	324	666	501	2854	1050	1134	861	390
Haes_INCU_HELI	0	0	573	0	0	0	0	768	621	0	0	0	0	501	1959	708	1134	0	0
Hani_GALA_GALA	0	840	0	0	444	486	447	768	621	594	699	324	666	501	2865	1281	1134	873	390
Haps_TINE_TINE_HAPS	750	0	573	0	444	486	447	768	621	0	699	324	666	501	2310	708	1134	0	0
Hbpy_DREP_DREP_THYA	0	0	0	0	0	0	0	0	0	0	0	0	0	0	2865	1281	1134	867	390
HeinEmon_PTER_PTER_PTER	750	840	573	402	444	486	447	768	621	594	699	324	666	501	2865	543	1134	870	390
Heli3_PAPI_NYMP_HELI	750	0	573	0	444	0	447	0	0	589	0	324	666	501	2865	456	0	0	390
Hens_GEOM_GEOM_ENNO	0	0	0	0	0	0	0	768	621	0	0	0	0	0	2865	1050	1134	0	0
Heol_PYRA_PYRA_PYRA	750	840	573	402	444	486	447	768	621	593	699	324	666	501	2865	1281	1134	870	390
Heter_ZYGA_EPIP	0	840	573	402	444	486	447	768	621	588	699	324	666	501	2865	939	0	0	390
Hfel_CHOR_CHOR	750	840	573	402	444	486	447	768	621	594	0	324	666	501	2862	1281	1134	873	390
Hibd_HYBL_HYBL	750	840	0	402	444	486	447	768	621	579	699	0	666	501	2865	939	1134	861	390
Hiku_GELE_OECO_STAT	750	840	573	402	0	486	447	768	621	594	699	324	666	501	2859	456	1134	513	0
Hila_TORT_TORT_CHLI	750	840	573	402	444	486	447	768	621	594	699	0	666	501	0	1281	1134	857	0
Hima_ZYGA_HIMA	0	0	573	402	444	486	0	0	0	0	699	324	0	501	2169	0	0	0	390
Hist_TORT_TORT_CHLI	750	840	573	402	444	486	447	768	621	0	699	324	666	501	2865	1050	1134	513	390
Hlch_GELE_BATR_BATR_sameasBatr	750	840	573	402	444	486	447	768	621	594	699	324	666	501	2864	1050	1134	873	390
Hlin_BOMB_SPHI_MACR	0	0	573	402	0	486	447	768	621	594	0	324	0	501	2865	1050	0	873	390
Hlpu_CALL_CALL_PTER	0	0	0	0	0	0	0	0	621	0	699	0	0	0	2169	456	0	0	390
Hmnt_TORT_HELI	750	840	573	402	444	486	447	768	621	594	0	324	666	501	2865	1281	1134	873	390
Hodn_GEOM_SEMA_SEMA	750	840	573	402	444	486	447	768	621	594	699	324	666	501	2865	1277	1134	873	390
Hova_HESP_HESP_HETE	750	0	0	0	0	0	0	0	621	0	699	0	666	501	2169	708	0	0	390
Hptr_GELE_ELAC_HYPE	750	840	573	402	444	486	447	768	621	594	699	0	666	501	2865	939	1134	873	390
Hrgi_GEOM_GEOM_STER	750	0	573	0	444	486	0	768	621	594	699	324	635	501	2865	1281	1134	867	390
Hrhd_BOMB_EUPT_JANI	750	840	573	402	444	486	447	768	621	594	699	324	666	501	2865	1236	1134	873	390
Hril_LASI_LASI_LASI	0	0	0	0	0	0	0	0	0	0	0	0	0	0	1830	1281	1116	822	390
Hsch_HESP_HESP_COEL	0	0	0	0	444	486	0	0	621	594	699	324	666	501	2169	456	0	0	390
Hsp2_HETE_HETE	750	840	573	0	444	486	447	768	621	594	699	0	666	501	2862	1050	1134	861	0
Htce_GELE_LECI_LECI	0	0	0	0	0	0	0	0	0	0	0	0	0	0	2865	1050	1134	873	390

Htmn_TORT_TORT_CHLI	750	840	573	0	444	486	447	768	621	594	699	324	666	501	2865	1050	1134	870	0
Htur_GELE_COSM	750	0	573	402	444	486	447	0	621	0	0	324	666	501	2865	1281	642	513	390
Hxen_GEOM_GEOM_GEOM	0	0	573	402	444	486	447	0	621	594	699	324	666	501	2865	1281	1134	870	390
Hybs_TINE_TINE_MEES	750	840	573	402	444	486	447	768	621	594	0	0	666	501	2862	1050	1134	864	390
Hyfm_HYBL_HYBL	750	840	573	402	444	486	423	768	0	594	699	0	666	501	2865	939	1134	873	390
Hymi_NOCT_ARCT_LITH	750	840	570	402	444	486	447	768	621	594	0	324	666	501	2463	1281	1134	873	390
Hysy_TRICHOPTERA	0	0	0	0	0	0	0	0	0	0	0	0	0	0	2856	708	1134	0	0
Hzea_NOCT_NOCT_HELI	0	840	573	402	444	486	0	768	621	594	0	324	0	0	2865	1050	1134	873	390
Idms_GEOM_GEOM_STER	750	840	573	402	444	486	447	768	621	594	699	324	666	0	2865	1281	1134	873	390
Illg_GELE_XYLO_XYLO	750	840	573	402	444	486	447	768	621	594	699	324	666	501	2169	1050	1134	873	390
Imja_IMMO_IMMI	0	0	0	0	0	0	0	0	0	0	0	0	0	0	2169	699	1134	0	390
Imsp_IMMO_IMMI	0	840	573	0	444	486	447	768	621	594	699	324	666	501	2865	1281	1134	873	390
Ipdt_NOCT_NOLI_COLL	750	840	0	0	444	486	447	768	621	594	699	324	666	501	2862	1281	1134	873	390
Jcer_BOMB_SATU_SATU	750	840	573	402	444	486	447	768	621	594	0	324	665	501	2865	1281	1134	873	390
Jpta_BOMB_EUPT_JANI	750	840	573	402	444	486	447	768	621	0	699	324	666	501	2865	1236	1134	513	390
Kalb_TINE_PSYC_NARY	750	840	573	402	444	486	447	768	621	594	699	324	666	501	2865	708	750	873	0
Kgr13_HEPI_HEPI_HEPI	750	840	573	402	441	486	447	768	621	594	699	0	666	501	2862	1050	1134	0	0
Klyd3_PAPI_PIER_COLI	750	0	573	402	0	486	0	766	621	594	699	324	666	501	2865	1050	1134	873	390
Ktr_GRAC_DOUG	750	840	558	402	444	486	447	768	621	576	699	324	666	501	2865	0	1134	821	390
Lact_ZYGA_LACT_uncertainID	750	840	573	402	444	486	447	768	621	594	699	324	666	501	2865	1255	1133	513	390
Laenes_INCU_PROD_LAMP	750	840	573	402	0	486	447	768	621	594	699	324	666	501	0	939	1134	0	390
Lch2_MIMA_MIMA	750	0	573	402	444	486	447	768	621	594	0	324	666	501	600	1281	1134	873	390
Lcr2_ZYGA_MEGA_MEGA	0	840	573	402	444	486	447	768	621	0	699	324	666	501	2862	1281	1133	873	390
Ldi_NOCT_LYMA_LYMA	750	840	573	402	444	486	447	768	621	594	699	324	666	501	2865	1281	0	513	390
Ldumi2_BOMB_BRAH_LEMO	750	0	573	402	444	486	447	768	621	594	699	324	666	501	2865	1281	0	802	390
Lebe_COSS_COSS_META	750	840	573	402	444	486	447	0	621	0	699	324	666	501	2865	1281	1134	873	390
Leci_GELE_LECI_LECI	750	840	573	402	444	486	447	768	621	594	699	324	666	501	2865	708	1134	873	390
Leuco_YPON_LYON_CEMI	0	0	573	402	441	486	447	0	621	594	699	0	666	501	2168	0	1134	0	0
Lica_PAPI_NYMP_LIBY	750	840	573	0	444	486	441	768	621	0	0	324	0	0	2865	0	1134	873	390
Liph_PAPI_LYCA_MILE	750	840	573	402	444	486	447	768	621	594	699	324	666	501	2103	1281	1134	0	390
Llun_COSS_COSS_HYPO	0	0	0	0	0	0	0	0	0	0	0	324	0	0	2841	939	0	873	390
Lmau_TINE_LYPU	0	0	0	0	0	0	0	0	0	0	0	0	0	0	2169	1050	1134	513	0
Loas_LOPH_LOPH	750	840	573	398	441	486	0	768	621	594	699	324	666	501	2859	708	0	870	0
Lpfe_YPON_LYON_LYON	0	0	570	0	444	486	447	768	621	594	0	324	666	501	2169	0	1134	819	0
Lpts_YPON_GLYP_GLYP	750	840	573	402	444	486	446	768	621	594	699	324	666	501	2865	1050	1134	873	390
Lquercus_LASI_LASI_LASI	750	840	573	402	444	486	447	768	621	594	699	324	666	501	2865	1281	1134	822	390
Lsci_GELE_XYLO_XYLO	0	840	573	402	444	486	447	768	621	594	699	324	666	501	2865	1281	1134	0	390
Lsub_ZYGA_LACT	750	840	573	402	444	486	447	768	621	594	699	324	666	501	741	699	1134	873	390
Lte_THYR_THYR_SICU	750	0	0	0	0	486	0	0	621	0	699	324	666	501	2166	243	1134	0	390
Lza_GEOM_URAN_URAN	750	840	573	402	444	486	0	768	621	594	699	324	666	501	2166	1281	1134	873	390
Mac5_MNES_MNES	750	840	573	402	444	484	447	768	621	594	699	0	666	501	2859	0	1131	0	0
Macro_PYRA_PYRA_GALL	750	840	573	402	444	486	447	768	621	588	0	324	666	501	2865	1281	1134	873	390
Mame2_LASI_LASI_LASI	750	0	0	0	444	486	447	768	621	594	699	324	0	0	1074	1050	1134	873	390
Mata_ZYGA_DALC	0	0	0	0	0	0	0	0	0	0	0	0	0	0	1569	1050	0	480	390
Mbal_NEPT_NEPT	0	0	573	396	444	0	447	768	621	0	699	0	666	501	2859	570	1134	0	390
Mbtl_TINE_TINE	750	840	573	402	444	486	447	768	621	594	699	324	666	501	2865	1050	1134	870	0

**Mca2_MICR_MICR**	750	840	573	402	441	486	447	768	621	594	699	324	666	501	0	939	1134	0	0
**Mchr_BOMB_ENDR_MIRI**	750	0	573	402	444	486	447	768	621	594	699	324	666	501	2865	1281	1134	810	390
**Mcls_GELE_GELE_ANOM**	0	0	573	402	444	0	444	0	621	594	0	324	0	501	2862	1281	1134	873	390
**Mcon_HEDY_HEDY**	750	0	0	402	444	486	447	0	621	0	699	324	666	501	2865	0	1134	825	390
**Mcuc2_SESI_SESI_SESI**	750	840	573	402	444	486	447	768	621	594	699	324	666	501	2865	1050	1134	861	390
**Mdyi_ZYGA_MEGA_TROS**	750	840	573	402	444	486	0	768	621	0	699	324	665	501	2862	939	1134	873	390
**Meem_GEOM_URAN_EPIP**	0	0	0	0	444	486	447	768	621	594	699	324	666	501	2865	1050	1134	873	390
**Merid_COPR_CARP**	750	840	573	402	444	486	447	768	621	594	699	324	666	501	2865	1281	1134	513	390
**Mfla_PYRA_PYRA_CHRY**	750	840	573	0	444	486	442	768	621	594	0	324	666	501	2865	1281	1134	873	390
**Mglp_ZYGA_MEGA**	750	840	573	402	444	486	447	768	621	594	0	324	665	501	2705	1281	1134	873	390
**Micr_MICR_MICR**	0	0	573	0	441	0	447	768	0	0	699	324	666	501	2850	0	0	0	0
**Mido_CHOR_CHOR_MILLI**	0	840	573	402	444	485	447	0	621	594	699	324	666	501	2865	1050	1134	510	390
**Mka_NOCT_MICR**	750	0	573	0	444	486	447	768	621	594	699	324	0	501	2865	1281	1134	870	390
**Mmax_DREP_DREP_DREP**	750	840	573	0	444	486	447	768	621	594	0	324	666	501	2865	1281	1134	873	0
**Mmi1_NOCT_NOLI_NOLI**	750	840	573	402	444	486	0	768	621	594	699	324	0	501	2865	1281	1134	873	390
**Mnau_COSS_COSS_META**	0	0	0	0	0	0	447	0	0	0	699	0	0	0	1569	0	0	0	390
**Mobu_TINE_TINE_SCAR**	750	0	573	0	444	486	447	0	0	594	699	0	666	501	2865	1281	1134	864	390
**Moch_TINE_TINE_MYRM**	750	840	573	402	444	486	447	768	621	594	699	324	666	501	2865	1050	1134	504	390
**Moin_TINE_TINE_MYRM**	750	0	573	402	0	486	447	768	621	594	699	324	666	501	2865	939	642	0	390
**Mom_GELE_COLE_MOMP**	750	840	573	402	444	486	447	0	621	594	699	0	666	501	2865	1281	1134	873	384
**Momph_GELE_COLE_MOMP**	0	0	0	0	0	0	0	0	0	0	0	0	0	0	2859	708	1134	522	384
**Mpll3_THYR_THRY_SICU**	750	0	573	402	444	486	0	768	621	594	699	324	0	501	2670	0	1134	0	390
**Mqrc_GELE_COSM_COSM**	750	840	573	402	0	486	447	768	621	594	699	324	0	501	2865	1281	1134	867	384
**Mqui_BOMB_SPHI_SPHI**	0	840	573	402	444	486	447	768	621	594	0	324	666	501	2865	939	1134	870	390
**Mrubi_LASI_LASI_LASI**	0	0	0	0	0	0	0	0	0	0	0	0	0	0	2865	1281	1134	822	390
**Msys_DREP_DREP_DREP**	750	840	573	402	444	486	447	768	621	594	699	324	666	501	2856	1050	1134	0	390
**Muso_PYRA_CRAM_MUSO**	750	840	573	402	0	486	447	768	621	594	697	324	666	501	2865	1281	1134	870	390
**Muyc_BOMB_ANTH_MUNY**	0	840	573	402	444	486	447	768	621	594	699	324	666	501	2865	1281	1134	870	390
**Myam_HESP_HESP_PYRR**	0	0	570	0	444	486	0	0	621	0	0	0	0	0	1536	0	0	0	390
**Nard_TINE_PSYC_NARY**	0	0	573	402	0	486	447	0	621	0	699	324	666	0	828	708	1134	0	0
**Nblt_PYRA_CRAM_NOOR**	750	840	573	402	444	486	447	0	621	594	0	324	0	501	2865	1026	1134	510	390
**Nclo_TINE_TINE_NEMA**	750	0	573	402	444	0	447	0	621	594	699	0	666	501	2865	1050	1134	0	390
**Nemo_INCU_ADEL_ADEL**	0	840	573	0	444	486	447	0	621	0	699	324	666	501	2862	1050	1134	0	384
**Nfla_BOMB_ANTH**	750	840	573	402	444	486	447	768	621	594	699	324	666	501	2659	1281	1134	819	390
**Niph_PYRA_CRAM_WURT**	750	840	573	402	444	486	447	768	621	594	699	324	666	501	2865	939	1134	870	390
**Nmec_NEOP_NEOP**	750	840	573	402	444	486	447	768	621	594	699	324	666	501	0	0	0	855	0
**Nmgs_INCU_ADEL_NEMA**	750	0	573	0	444	486	447	768	621	594	699	0	666	501	2859	0	1134	519	0
**Nora_ZYGA_MEGA**	0	0	0	0	0	0	0	0	0	0	0	0	0	0	2169	939	1134	873	390
**Nothsp_GEOM_SEMA_SEMA**	750	840	573	402	444	486	447	768	621	579	699	324	666	501	2865	1278	1134	822	0
**Nsig_NOCT_NOLI_CHLO**	750	840	573	402	444	486	447	768	621	594	699	324	666	501	2865	1281	1134	867	390
**Nspin_GELE_COLE_BLAS**	0	0	0	402	444	486	447	0	621	590	0	0	0	501	2757	1281	1134	0	390
**Octg_ZYGA_MEGA**	0	0	0	0	0	0	0	0	0	0	0	0	0	0	2169	456	750	873	390
**Odle_GELE_LECI_ODIT**	0	840	573	402	444	486	447	768	621	594	699	324	666	501	2865	1281	1134	873	390
**Odrp_HEPI_HEPI**	750	840	573	402	441	486	447	768	0	594	699	0	666	501	2862	1050	1134	0	0
**Odsp_GEOM_GEOM_ENNO**	0	0	573	0	0	0	447	768	621	0	0	0	0	0	2865	1050	1134	0	0
**Oeno_NOCT_OENO**	750	840	573	402	444	485	447	768	621	594	699	324	0	501	2865	1281	1134	0	390

Ofo_BOMB_ENDR_PRIS	750	0	573	0	444	486	447	768	621	592	699	324	666	501	2865	1281	1134	873	390
Olfa_TORT_TORT_OLET	0	840	573	402	444	486	447	768	621	594	699	0	666	501	2865	1281	1134	873	0
Omod_BOMB_SATU_OXYT	750	0	573	0	0	486	0	656	621	594	699	324	0	501	2169	1050	0	525	390
Onst_GEOM_GEOM_LARE	0	840	573	402	444	486	447	768	621	594	699	324	666	501	2862	1281	1134	873	390
Opin1_YPON_YPON	0	0	573	0	0	0	447	0	621	0	0	0	0	501	2865	1050	1134	0	0
Opns_NEPT_OPOS	0	0	0	0	0	0	0	0	621	0	0	0	0	0	1568	0	0	0	0
Oreo_TINE_PSYC_OIKE	750	0	573	0	444	0	0	0	621	0	699	0	666	501	2865	0	642	0	0
Oros_DREP_DREP_DREP	750	840	573	402	444	486	447	768	621	592	699	324	666	501	2865	939	1134	864	390
Ospa_YPON_GLYP_ORTH	750	840	573	402	444	486	447	768	621	594	699	324	0	501	2865	570	1134	0	390
Othi_TINE_TINE_HIER	0	840	573	0	444	486	447	768	621	594	0	324	666	501	2865	1281	1134	873	390
Ound_HEPI_PALA	750	840	573	402	441	486	0	768	621	594	0	0	666	501	0	708	1134	816	0
Oure_YPON_YPSO	750	840	573	402	444	486	447	0	621	572	699	324	666	501	2865	1050	1134	510	390
PBO_BOMB_PHID	750	0	573	402	444	486	0	768	621	594	699	324	0	0	2865	1281	0	0	390
Pacer_ERIO_checkID	750	840	573	0	444	486	447	0	621	594	699	324	666	501	2859	0	1134	867	0
Panla_GELE_COSM_ANTE	0	0	0	0	0	0	0	0	0	0	0	0	0	0	2865	1050	0	861	390
Pasma_TORT_TORT_OLET	750	840	573	402	444	486	447	768	621	594	699	324	666	501	2865	1050	1134	864	390
Pasp_TORT_TORT_TORT	628	840	0	402	444	486	447	768	621	594	699	0	0	501	2865	456	1134	0	0
Pays_SESI_CAST_CAST	750	0	573	402	444	486	447	768	621	0	699	324	666	501	2865	1281	1134	513	387
Pbod_SESI_BRAC_PSEU	0	0	573	0	0	0	0	0	621	0	699	324	0	0	1569	0	0	0	390
Pcan_INCU_INCU	750	0	573	0	0	486	447	768	621	0	699	0	666	501	2169	0	1134	0	0
Pccc_MICR_MICR	750	840	573	0	441	486	447	768	621	594	699	0	666	501	2853	708	0	0	0
Pcoci_TINE_TINE	750	0	0	0	444	486	447	768	621	0	699	324	666	501	2865	570	1134	0	390
Pcon_PYRA_CRAM_NYMP	750	840	573	402	444	486	447	768	621	594	699	324	666	501	2865	1281	1134	864	390
Pcra_TINE_PSYC_PSYC	0	0	0	0	0	486	447	0	0	0	699	0	0	501	828	0	0	0	0
Pctb_TRICHOPTERA	0	0	0	0	0	0	0	0	0	0	0	0	0	0	2856	687	1134	522	0
Pcym_DREP_DREP_DREP	750	840	573	402	444	486	447	768	621	594	0	324	666	501	2865	1281	1134	822	390
PdbaPeud_TINE_PSYC_PSEU	750	0	531	402	0	0	447	0	621	0	699	0	666	501	2865	708	0	513	0
Pdbi_ZYGA_LIMA_LIMA	750	840	573	402	444	486	447	768	621	594	699	324	666	501	2670	939	1134	873	390
Pect_NEPT_NEPT_PECT	750	840	573	396	444	486	447	768	621	579	699	0	666	501	0	708	1134	0	390
Pedy_PAPI_LYCA_PORI	0	0	0	0	0	0	0	768	0	0	0	0	0	0	2864	1050	1134	0	0
Peri_TINE_TINE_PERI	750	0	573	402	444	486	447	768	621	594	699	324	666	501	2865	1281	0	0	390
Pfene_BOMB_ENDR_PRIS	750	0	573	402	444	486	447	768	621	594	699	0	666	501	2157	1281	1134	873	390
Pfla_THYR_THYR_STRI	0	0	573	402	444	486	0	768	621	0	699	324	0	501	2169	0	1134	873	390
Pfoc_COSS_COSS_HYPO	0	840	573	402	444	486	447	768	621	594	699	324	666	501	2865	1281	1134	873	390
Pgah_YPON_PLUT	750	0	570	402	444	486	447	768	621	0	699	0	666	501	2865	1026	1134	0	389
Pgos_GELE_GELE_PEXI	750	0	573	402	444	486	447	768	621	594	699	324	666	501	2865	1281	1134	870	390
Phau_ZYGA_ZYGA_PHAU_problemID	750	840	573	402	444	486	447	768	621	594	699	324	666	501	2310	708	1134	804	390
Phcn_GRAC_GRAC_PHYL	750	840	573	402	444	486	447	766	0	594	699	324	666	501	2856	1050	1134	846	390
Phfr_NOCT_NOCT_STRE	750	840	573	402	444	486	447	768	621	594	0	324	666	501	2157	1281	1134	873	390
Phih_ZYGA_LIMA_LIMA	750	840	573	402	444	486	447	768	0	594	0	324	0	501	2865	1050	1133	873	390
Phms_SESI_SESI_TINT	739	840	573	402	444	486	447	768	621	593	699	324	666	501	2169	1050	1134	870	375
Phyl_GRAC_GRAC_LITH	750	840	555	402	444	486	447	0	621	594	699	324	666	501	2865	0	1134	0	390
Pida2_TORT_TORT_TORT	750	840	573	402	444	486	447	768	621	594	699	324	666	501	2865	1050	1134	864	390
Pin_PYRA_PYRA_PHYC	750	840	573	401	444	486	447	768	621	594	699	324	666	501	2862	1281	1134	873	387
Piph3_PAPI_PAPI_PAPI	750	0	573	402	444	486	447	0	621	594	0	324	0	501	2865	1050	0	867	390

**Pjez_GELE_ELAC_STEN**	0	0	573	402	441	486	447	0	621	594	0	324	0	501	2865	1050	1134	867	0
**Platc_NOCT_NOCT_NOCT**	750	840	573	402	444	486	447	768	621	594	0	324	666	501	2865	1281	1134	873	390
**Plty_PTER_PTER_PTER**	750	840	573	402	444	486	0	768	621	594	0	324	666	501	2169	1050	0	510	390
**Pmel_DREP_EPIC**	750	0	0	402	444	486	447	0	621	0	699	324	666	0	2865	1281	1134	873	390
**Pmgl_GRAC_GRAC_PHYL**	750	0	555	402	444	486	447	0	621	594	699	324	666	501	2856	0	1134	837	389
**Pmyo_BOMB_SPHI_SMER**	750	840	573	402	444	486	447	768	621	590	699	324	666	501	2169	1050	0	870	390
**Pncla_BOMB_EUPT_PANA**	750	0	573	402	444	486	447	768	621	588	699	324	666	501	2865	1050	1134	510	390
**Pnex_PYRA_CRAM_PYRA**	750	0	573	0	444	486	447	768	621	594	0	0	666	501	2865	1050	1134	870	390
**Pntp_ZYGA_LIMA_LIMA**	750	0	573	0	444	486	447	768	621	594	699	324	666	501	2865	939	0	873	390
**Poeu_CHOR_CHOR_CHOR**	750	840	573	402	444	486	447	768	621	594	699	324	0	0	2865	1281	1134	864	390
**Pohd_HEPI_HEPI**	750	840	573	402	441	486	447	768	621	594	699	0	666	501	2646	455	1134	0	0
**Posp_ZYGA_ZYGA_PROCmay be**	750	840	573	402	444	486	447	751	621	0	699	0	666	501	2169	243	1121	873	390
**Ppls_COSS_COSS_ZEUZ**	750	840	573	402	444	486	447	768	621	576	699	324	666	501	2862	1050	1134	873	390
**Ppop_LASI_LASI_POEC**	750	0	573	402	444	486	438	768	621	594	699	324	666	501	2865	1281	1134	870	390
**Ppra_COPR_COPR**	750	0	573	402	444	486	447	0	621	0	699	0	666	501	2865	243	0	873	390
**Ppy122_PYRA_CRAM_PYRA**	750	840	276	402	444	486	447	768	621	594	699	324	666	501	2865	1281	1134	870	390
**Pqu_INCU_PROD_PROD**	750	840	573	0	444	486	447	768	621	594	699	324	666	501	0	570	1134	0	0
**Pquadr2_NEPT_OPOS_OPOS**	747	0	573	402	444	486	447	768	621	593	699	0	666	501	2853	708	0	0	390
**Prap_PAPI_PIER_PIER**	750	0	573	402	444	486	447	768	621	594	699	324	666	501	2865	1281	1134	873	390
**Prbn_GRAC_GRAC_GRAC**	0	0	573	402	444	486	447	768	621	0	699	0	666	501	2865	708	1134	0	0
**Prfx_GELE_AMPH**	0	840	573	402	444	486	447	768	621	594	699	324	666	501	2865	1281	1134	870	390
**Prob2_COSS_COSS_COSS**	750	840	573	402	444	486	447	768	621	594	699	324	666	501	2434	1281	1134	873	390
**Proto_HEPI_PROT**	0	0	0	402	441	0	0	0	0	0	699	0	666	501	861	456	0	0	0
**Pryo_YPON_YPON_PRAY**	750	840	573	402	444	486	447	0	621	594	699	324	663	501	2859	939	1134	0	390
**Psasp_TINE_TINE_HIER**	750	840	573	0	444	486	447	0	0	585	699	324	666	501	2865	1050	1134	864	390
**Psc2_NOCT_NOCT_HYPE**	750	840	573	402	444	486	447	768	621	594	699	0	666	501	2865	1281	1134	873	390
**Psdl_NOCT_NOCT_NOCT**	750	840	573	402	444	485	447	768	621	594	0	324	666	501	2865	1281	1134	873	390
**Psh29_PYRA_CRAM_SCHO**	750	840	573	402	444	486	447	0	621	594	699	324	666	501	2865	939	1134	867	390
**Psin_ZYGA_ZYGA_PHAU**	750	840	573	402	444	486	447	768	621	594	699	324	666	501	2865	1257	1134	864	390
**Psy2_SESI_SESI_SESI**	750	840	573	402	444	486	447	768	621	594	699	324	666	501	2865	1281	1134	861	390
**Ptdx_PAPI_PIER_PSEU**	0	840	573	402	443	486	447	768	621	594	699	324	656	501	2862	1050	1131	0	390
**Pter_CALL_CALL**	750	840	573	402	444	486	447	768	621	594	0	0	666	501	0	0	0	0	0
**Ptfe_CALL_CALL_CALL**	750	840	573	402	444	486	447	768	621	594	699	324	666	501	2865	1281	1134	873	390
**Ptha_PAPI_NYMP_NYMP**	750	840	573	402	444	486	447	768	621	594	699	324	666	501	2865	1281	642	873	390
**Ptus_PALA_PALA**	0	0	0	0	0	0	0	0	0	0	0	0	0	0	2169	0	1134	498	390
**Ptys_PALA_PALA**	750	840	573	0	444	486	447	768	621	594	699	324	666	501	2862	1050	1134	0	387
**Purg_DItrysia**	0	0	0	402	0	0	447	0	621	0	699	0	0	501	2169	456	0	0	390
**Putr_TINE_TINE_TINE**	0	0	573	0	0	0	447	768	0	0	0	0	0	0	2166	993	0	0	0
**Pvol_TORT_TORT_CHLI**	750	0	573	402	444	486	447	768	621	0	699	324	666	501	2865	456	1134	0	390
**Pxy_YPON_PLUT**	747	840	573	0	444	486	447	768	621	594	699	324	0	501	2169	1281	1134	858	0
**Pymi_TINE_TINE_HIER**	750	840	573	402	444	486	447	768	621	594	0	324	0	501	2865	1050	1134	0	390
**Quid_BOMB_BOMB_EPII**	750	0	0	402	444	486	447	0	621	594	699	324	666	501	2865	1273	0	819	390
**Rfal_GELE_LECI**	0	0	0	0	0	0	0	0	0	0	0	0	0	0	2865	1050	1134	870	390
**Rham_GELE_XYLO_SCYT**	750	840	573	402	444	486	447	768	621	594	699	324	666	501	2865	1281	1134	849	390
**Rhmd_TRICHOPTERA**	0	0	0	0	0	0	0	0	0	0	0	0	0	0	2859	708	0	0	0

**Rpro3_NOCT_NOCT_RIVU**	750	840	573	402	444	486	447	768	621	594	699	324	666	501	2865	1281	1134	864	390
**Rstm_GRAC_ROES**	750	840	558	0	444	486	447	0	621	594	699	324	666	501	2865	939	1134	0	390
**Sapp_TINE_TINE_SCAR**	750	0	573	402	444	486	447	768	0	594	0	324	666	501	2865	939	1134	867	390
**Scdy_TINE_PSYC_SCOR**	750	0	573	402	0	0	447	768	621	594	0	324	666	501	2865	939	1134	525	0
**Schrk_SCHR_SCHR**	0	0	0	0	0	0	0	0	0	0	0	0	0	0	2310	708	1134	873	390
**Scli_GEOM_GEOM_STER**	750	840	573	401	444	486	447	768	621	579	699	324	666	501	2865	1281	1134	873	390
**ScspSC_GELE_uncertainID**	750	840	573	402	444	486	447	768	621	594	699	324	666	501	2859	1050	1134	861	390
**Scsp_PYRA_CRAM_SCOP**	642	840	573	402	444	486	447	768	621	594	699	324	666	501	2865	1281	1134	867	0
**Sdoe_NOCT_NOTO_PYGA**	750	840	573	402	444	486	447	768	621	592	699	324	666	501	2865	1050	1134	873	390
**Sdru_GEOM_URNA_EPIP**	699	840	0	0	444	486	447	768	621	594	699	324	666	501	2157	1281	1134	873	390
**Seic_TRICHOPTERA**	0	0	0	0	0	0	0	0	0	0	0	0	0	0	2859	708	1134	843	0
**Seto_Ditrysia**	750	840	573	0	444	486	447	0	621	594	699	324	666	501	2865	1281	1134	873	390
**Seur2_PAPI_LYCA_THEC**	750	840	573	402	444	486	447	0	621	0	699	324	666	501	2865	1281	1134	0	390
**Sfr_NOCT_NOCT_NOCT**	750	840	573	402	444	486	447	768	621	594	699	324	666	501	2865	1281	1134	873	390
**Simm_GELE_XYLO_SCYT**	750	840	573	402	444	486	447	768	621	594	699	309	666	501	2862	1050	1134	860	390
**Sin_PYRA_CRAM_CYBA**	750	840	573	402	444	486	447	768	621	594	0	324	666	501	2865	1281	1134	867	390
**Sktn_SCHR_SCHR**	750	840	573	402	444	486	447	767	621	589	699	324	665	501	2310	708	1134	867	390
**Smim2_COPR_CARP**	750	840	573	402	444	486	447	768	621	594	699	324	666	501	2865	1281	1134	873	390
**Snaes_BOMB_SATU_SATU**	750	840	573	402	444	486	447	768	621	594	699	324	665	501	2865	1281	0	822	390
**Spla_SESI_CAST**	750	840	573	402	444	486	447	768	621	594	699	324	666	501	2865	1281	1134	873	390
**Sput_GRAC_GRAC_GRAC**	750	840	558	402	0	486	0	0	621	594	699	324	666	501	2841	0	1134	864	390
**Srtc_TORT_TORT_TORT**	0	0	573	402	444	486	447	0	621	594	0	324	666	501	2658	1050	1134	510	390
**Sski_THYR_THYR_STRI**	750	840	573	402	444	486	447	768	621	594	699	324	666	501	2865	708	1134	867	390
**Stan_NEPT_NEPT_NEPT**	750	840	570	393	0	486	447	768	621	594	0	0	666	501	2859	1050	0	0	0
**Stmp_GELE_OECO_STAT**	750	840	573	402	444	486	446	768	621	594	699	324	666	501	2859	1050	1134	852	390
**Stna_PYRA_CRAM_ODON**	750	840	573	0	444	486	447	0	621	594	699	324	666	501	2865	0	1134	867	0
**Stpa_GELE_ELAC_STEN**	0	0	0	0	444	486	0	0	621	0	699	0	0	0	2169	456	750	0	390
**Svta_ZYGA_LIMA_CHRY**	750	840	573	402	444	486	447	768	621	592	699	324	666	501	2865	1281	1134	873	390
**Syco_SESI_BRAC**	0	0	0	0	0	0	0	0	621	0	0	0	0	0	2169	456	0	0	0
**Syex_SESI_SESI_SESI**	750	840	573	402	444	486	447	768	621	594	699	324	665	501	2865	707	1134	861	390
**Sytg_YPON_YPON_SCYT**	750	0	573	402	444	486	0	0	0	594	699	0	666	501	1671	708	642	0	0
**Szo2_MICR_MICR**	750	840	573	0	441	486	447	768	621	0	699	324	666	501	0	708	1131	0	0
**TB032177_BOMB_CART**	750	840	573	402	444	486	0	768	621	594	699	324	666	501	2865	1281	0	825	390
**Tan_YPON_YPON_YPON**	750	840	0	402	444	486	447	768	621	579	699	324	657	501	2862	939	1134	846	390
**Tbi3_TINE_TINE_TINE**	750	840	573	402	444	486	447	768	621	0	699	324	666	501	2842	1281	861	522	390
**Tchmp_NOCT_NOCT_PANT**	750	840	573	402	444	486	447	768	621	594	0	324	666	501	2865	1050	1134	0	390
**Tco2_TINE_TINE_TINE**	750	840	573	402	444	486	447	768	0	0	699	324	666	501	2865	1281	642	759	390
**Tep2_TINE_PSYC_OIKE**	750	840	573	402	444	486	447	768	621	556	699	324	666	501	2865	1281	1134	849	390
**Tfu2_INCU_PROD_LAMP**	0	840	573	402	444	486	447	768	621	594	699	324	666	501	2862	708	1134	0	390
**Tgkb_TISC_TISC**	0	0	573	0	444	486	447	0	621	0	699	0	666	501	2862	708	0	0	390
**Thap_GELE_ELAC_HYPE**	0	0	0	0	0	0	0	0	0	0	0	0	0	0	2169	993	1134	0	390
**Thcn_TISC_TISC**	0	0	573	0	444	486	447	0	621	594	699	0	666	501	2859	0	1134	0	0
**Tlic_SESI_CAST_CAST**	750	840	573	402	0	486	447	768	621	594	0	324	0	501	2865	1050	1134	873	387
**Tmic_CHOR_CHOR_CHOR**	0	0	573	0	444	486	447	768	621	594	699	324	666	501	2310	1281	1134	864	390
**Tni_NOCT_NOCT_PLUS**	750	840	573	402	444	486	447	768	621	594	0	324	666	501	2146	1281	1134	873	390
**Tnot_LASI_LASI_MACR**	750	0	573	0	444	486	447	768	621	594	699	324	666	501	2865	1050	1134	0	390

**Tort_CHOR_CHOR_CHOR**	750	840	573	0	0	486	447	768	621	594	699	324	666	501	2865	1050	1134	873	390
**Tpad_NEPT_NEPT_NEPT**	750	0	573	0	0	486	447	768	0	594	699	0	0	501	2364	0	0	0	390
**Tpgt_TINE_TINE_TINE**	0	0	0	0	0	0	0	0	0	0	0	0	0	0	2865	708	1134	0	390
**Tpit_NOCT_NOTO_THAU**	750	0	0	0	0	486	0	768	0	0	699	324	666	501	2310	1281	1134	873	390
**Trca_GEOM_GEOM_LARE**	750	840	572	402	444	486	447	768	621	594	699	324	666	501	2865	1281	1134	858	390
**Trili_TRICHOPTERA**	750	840	573	402	0	486	447	768	621	594	699	0	666	501	2145	708	1134	513	0
**Trta_HEPI_HEPI**	750	840	573	402	441	485	447	768	621	594	699	0	666	501	2862	1050	1134	0	0
**Tsav_MIMA_MIMA**	0	0	573	402	0	486	447	763	621	594	699	324	666	501	2865	1257	1134	873	390
**Ttms_DREP_DREP_THYA**	750	0	573	402	444	486	447	768	621	594	699	324	0	501	2865	1281	1134	864	390
**Tycl_TINE_PSYC_TYPH**	0	0	0	0	0	486	447	0	621	594	699	324	0	501	2865	1050	0	870	390
**Tymb_GELE_XYLO_XYLO**	750	840	573	402	444	484	447	768	621	594	699	324	666	501	2865	1281	1134	873	390
**Tyu2_INCU_PROD_PROD**	750	840	573	402	444	486	447	768	621	594	699	324	666	501	2862	1281	1134	0	0
**Udo_HESP_HESP_PYRG**	0	0	567	402	444	486	447	768	621	578	699	324	0	501	2865	699	1134	864	390
**Uptd3_GEOM_URAN_URAN**	750	840	573	402	444	486	447	0	621	594	699	324	666	501	2865	939	1134	513	390
**Ursp_UROD_UROD**	750	840	573	402	444	486	447	768	0	594	699	324	666	501	2434	801	1134	873	390
**Vane_PAPI_NYMP_NYMP**	750	840	573	0	444	486	0	768	621	0	699	324	666	501	2310	1281	1134	870	390
**Vipo_SESI_SESI_PARA**	750	840	573	402	441	486	447	768	621	591	0	324	666	501	2865	708	1134	0	387
**Vqu2_INCU_INCU**	0	840	573	0	444	486	447	768	621	594	699	324	666	501	2165	708	1134	0	0
**Wasp_UROD_UROD**	0	0	0	0	0	0	0	0	0	0	0	0	0	0	2862	939	1134	867	390
**Win_GELE_OECO_OECO**	750	840	573	402	444	486	446	768	621	583	0	324	666	501	2865	1281	1134	870	390
**Wmo2_TRICHOPTERA**	0	0	573	402	444	486	447	768	621	576	699	0	666	501	2856	993	1134	837	0
**Woc_UROD_UROD**	0	0	573	0	0	0	447	768	621	0	0	0	0	501	2865	0	1134	0	0
**Xmns_COSS_COSS_ZEUZ**	750	0	573	402	444	486	447	768	0	594	699	324	666	501	2865	1050	750	501	390
**Xwi_TINE_TINE_MYRM**	750	840	573	402	444	486	447	768	621	594	699	324	666	501	2865	1281	1134	873	387
**Ymul_YPON_YPON_YPON**	750	840	573	0	444	486	447	768	621	594	699	324	666	501	0	1281	1134	771	0
**Yni_YPON_YPSO_YPSO**	750	840	573	402	444	486	447	768	621	307	0	324	666	501	2865	1281	1134	864	390
**Ysp_YPON_YPON**	750	840	573	402	444	486	447	768	621	594	699	324	666	501	2865	1281	1134	0	390
**Zcof_COSS_COSS_ZEUZ**	750	840	573	402	444	486	447	768	621	594	699	324	666	501	2865	1050	1134	873	390
**Zgfa_ZYGA_ZYGA_ZYGA**	750	840	0	0	444	486	447	768	621	0	699	324	0	501	2865	1050	1134	870	390
GENE NAME ➔	40fin	42fin	109fin	192fin	197fin	262fin	265fin	268fin	3007fin	3017fin	3070fin	8028fin	8091fin	ACC	CAD	DDC	Enolase	Period	WG
MAX. SUM ATTEMPTED PER GENE PER TAXON (BP) ➔	750	840	573	402	444	486	447	768	621	594	699	324	666	501	2865	1281	1134	873	390
% OF THEORETICAL MAX. SUM ACTUALLY SEQUENCED PER GENE FOR ALL TAXA COMBINED ➔	69.6	61.1	83.4	65.2	78.9	84.5	81.6	71.8	86.8	72.4	74.1	71.2	74.5	85.7	90.3	71.3	84.7	61.9	77.2

*, For purposes of these calculations, "N", "?", and "-" were excluded from the data set.
