# Supplementary material for: A Large-Scale, Higher-Level, Molecular Phylogenetic Study of the Insect Order Lepidoptera (Moths and Butterflies)
Source: PLoS One. 2013 Mar 12;8(3):e58568. doi: 10.1371/journal.pone.0058568 (PMC3595289; doi:10.1371/journal.pone.0058568)
Supplement: Text S1 — List of taxon subsets used to generate (by deletion) new data sets with reduced numbers of taxa. (DOC) [file pone.0058568.s008.doc]

**Text S1.** List of taxon subsets used to generate (by deletion) new data subsets with reduced numbers of taxa.

**Starting 436-taxon, 19-gene data matrix for construction of data subsets**:

nt123: "nt123.lep483tx14658char.mask.TAXSETS.1Dec10.nex"

nt123_degen1: "degen1.lep483tx14658char.mask.TAXSETS.1Dec10.nex"

| **Data type** | **Data subset desciption**^1^ | **Taxset(s) deleted**^2^ |
| --- | --- | --- |
| nt123 | 455 taxa,  no AC rogues | ApoROGUE28tx.from483tx19gnNt123 |
| nt123 | 432 taxa,  no RNR rogues | 51txROGUE.from483tx.nt123.jay |
| nt123 | 356 taxa,  no RNR rogues,  no heterogeneous taxa | 51txROGUE.from483tx.nt123.jay, 82txHETEROG.from483tx.nt123 |
| nt123 | 344 taxa,  Apoditrysia | nonDitrysia,  TINE,  GRAC.YPON |
| nt123 | 316 taxa,  Apoditrysia,  no AC rogues | nonDitrysia TINE GRAC.YPON, ApoROGUE28tx.from483tx19gnNt123 |
| nt123 | 133 taxa,  Macroheterocera + Pyraloidea | nonDitrysia,  TINE,  GRAC.YPON,  Apodit.noMacroPYRA.483tx |
| nt123 | 129 taxa,  Macroheterocera + Pyraloidea,  no AC rogues | nonDitrysia,  TINE,  GRAC.YPON,  Apodit.noMacroPYRA.483tx,  ApoROGUE28tx.from483tx19gnNt123 |
| nt123_degen1 | 453 taxa,  no AC rogues | ApoROGUE30tx.483tx19gnDegen1 |
| nt123_degen1 | 436 taxa,  no RNR rogues | 47txROGUE.from483tx.degen1.jay |
| nt123_degen1 | 428 taxa,  no RNR rogues,  no heterogeneous taxa | 47txROGUE.from483tx.degen1.jay,  10txHETEROG.from483tx.degen1 |
| nt123_degen1 | 434 taxa,  no RNR rogues,  no Acanthopteroctetidae,  no Neopseustidae: *Neopseustis* sp. | 47txROGUE.from483tx.degen1.jay,  Aun2_ACAN_ACAN,  Nmec_NEOP_NEOP |
| nt123_degen1 | 344 taxa,  Apoditrysia | nonDitrysia,  TINE GRAC.YPON |
| nt123_degen1 | 314 taxa,  Apoditrysia,  no AC rogues | nonDitrysia,  TINE,  GRAC.YPON,  ApoROGUE30tx.483tx19gnDegen1 |
| nt123_degen1 | 133 taxa,  Macroheterocera + Pyraloidea | nonDitrysia,  TINE,  GRAC.YPON,  Apodit.noMacroPYRA.483tx |
| nt123_degen1 | 128 taxa,  Macroheterocera + Pyraloidea,  no AC rogues | nonDitrysia,  TINE,  GRAC.YPON, Apodit.noMacroPYRA.483tx,  ApoROGUE30tx.483tx19gnDegen1 |

^1^ The compositional heterogeneity of the three sets of "heterogeneous" taxa are described in Figure 4. *no AC rogues*, no rogue taxa defined by the *Adams-consensus* approach (see Materials and Methods);

*no RNR rogues*, no rogue taxa defined by the *RNR* approach (see Materials and Methods).

^2^  Taxon subsets are shown immediately below.

**TAXSETS ApoROGUE28tx.from483tx19gnNt123 =**

Axsp_CIME_CIME

Cysu_DREP_DREP_CYCL

Pbod_SESI_BRAC_PSEU

Acro_GEOM_URAN_MICR

Cyna_ZYGA_CYCL

Cysp_ZYGA_CYCL

Copro_COPR_COPR

Cosla_COSS_COSS_COSS

Doa_NOCT_DOID

Lebe_COSS_COSS_META

Lmau_TINE_LYPU

Heter_ZYGA_EPIP

Enaw_ZYGA_EPIP_EPIP

Esp2_ZYGA_EPIP

Adam_PTER_PTER_AGDI

Agdi_PTER_PTER_MACR

Alsp_ALUC_ALUC

Bren_CHOR_CHOR_BREN

Epmn_EPER_EPER

Hani_GALA_GALA

HeinEmon_PTER_PTER_PTER

Hlch_GELE_BATR_BATR_sameasBatr

Hmnt_TORT_HELI

Ktr_GRAC_DOUG

Mido_CHOR_CHOR_MILLI

Mnau_COSS_COSS_META

Phms_SESI_SESI_TINT

Purg_DItrysia

**TAXSET ApoROGUE30tx.483tx19gnDegen1 =**

PBO_BOMB_PHID

Cmtn_LASI_LASI_CHIO

Cysu_DREP_DREP_CYCL

Axsp_CIME_CIME

Copro_COPR_COPR

Dfag_GELE_CHIM

Heter_ZYGA_EPIP

Enaw_ZYGA_EPIP_EPIP

Esp2_ZYGA_EPIP

Cill_GELE_ELAC_ELAC

Eten_GELE_ELAC_ELAC

AK142_SESI_BRAC

Agdi_PTER_PTER_MACR

Alsp_ALUC_ALUC

Cosla_COSS_COSS_COSS

Doa_NOCT_DOID

Hani_GALA_GALA

Hmnt_TORT_HELI

Ktr_GRAC_DOUG

Lebe_COSS_COSS_META

Lmau_TINE_LYPU

Mido_CHOR_CHOR_MILLI

Mnau_COSS_COSS_META

Pbod_SESI_BRAC_PSEU

Purg_DItrysia

Cyna_ZYGA_CYCL

Cysp_ZYGA_CYCL

Phms_SESI_SESI_TINT

Epmn_EPER_EPER

Syco_SESI_BRAC

**TAXSET 51txROGUE.from483tx.nt123.jay =**

Leuco_YPON_LYON_CEMI

Avld_NEOP_NEOP

Ldi_NOCT_LYMA_LYMA

Othi_TINE_TINE_HIER

Alsp_ALUC_ALUC

Fbrn_GELE_ELAC_DEPR

Prfx_GELE_AMPH

Ipdt_NOCT_NOLI_COLL

Ancy_TORT_TORT_OLET

Myam_HESP_HESP_PYRR

Axsp_CIME_CIME

Muso_PYRA_CRAM_MUSO

Sski_THYR_THYR_STRI

Agus_TRICHOPTERA

Cet_HESP_HESP_HESP

Dplex_PAPI_NYMP_DANA

Nclo_TINE_TINE_NEMA

Moin_TINE_TINE_MYRM

Hani_GALA_GALA

Ktr_GRAC_DOUG

Pntp_ZYGA_LIMA_LIMA

Purg_DItrysia

Copro_COPR_COPR

Agdi_PTER_PTER_MACR

Mido_CHOR_CHOR_MILLI

Proto_HEPI_PROT

Pedy_PAPI_LYCA_PORI

Cosla_COSS_COSS_COSS

Pbod_SESI_BRAC_PSEU

Gprot2_GELE_AUTO_SYMM

Lmau_TINE_LYPU

Pryo_YPON_YPON_PRAY

Stna_PYRA_CRAM_ODON

Dfag_GELE_CHIM

Rhmd_TRICHOPTERA

Eeu_GELE_ELAC_ETHM_

Bucc_GRAC_BUCC

Sput_GRAC_GRAC_GRAC

Lpfe_YPON_LYON_LYON

Psh29_PYRA_CRAM_SCHO

Psasp_TINE_TINE_HIER

Atpu2_YPON_YPON

Hlch_GELE_BATR_BATR_sameasBatr

Heli3_PAPI_NYMP_HELI

Clph_GELE_COLE_COLE

Mbtl_TINE_TINE

Phms_SESI_SESI_TINT

Hmnt_TORT_HELI

Cdel_GRAC_GRAC_GRAC

Lact_ZYGA_LACT_uncertainID

Nmec_NEOP_NEOP

**TAXSET 47txROGUE.from483tx.degen1.jay =**

Lmau_TINE_LYPU

Lpfe_YPON_LYON_LYON

Nclo_TINE_TINE_NEMA

Hlch_GELE_BATR_BATR_sameasBatr

Dfag_GELE_CHIM

Pcon_PYRA_CRAM_NYMP

Gprot2_GELE_AUTO_SYMM

Atpu2_YPON_YPON

Fbrn_GELE_ELAC_DEPR

Alsp_ALUC_ALUC

Dals_MIMA_MIMA

Avld_NEOP_NEOP

Pryo_YPON_YPON_PRAY

Micr_MICR_MICR

Muso_PYRA_CRAM_MUSO

Prfx_GELE_AMPH

Cet_HESP_HESP_HESP

Eutr_TORT_TORT_TORT

Nard_TINE_PSYC_NARY

Ldi_NOCT_LYMA_LYMA

Rpro3_NOCT_NOCT_RIVU

Pbod_SESI_BRAC_PSEU

Agdi_PTER_PTER_MACR

Axsp_CIME_CIME

Mido_CHOR_CHOR_MILLI

Bedg2_MIMA_MIMA

Purg_DItrysia

Cosla_COSS_COSS_COSS

Ancy_TORT_TORT_OLET

Moin_TINE_TINE_MYRM

Othi_TINE_TINE_HIER

Gyrt_NOCT_NOCTquad_STIC

Caul_NOCT_NOCT_CATO

Dyso_THYR_THYR_THYR

Copro_COPR_COPR

Hani_GALA_GALA

Anne_TORT_TORT_TORT

Tni_NOCT_NOCT_PLUS

Mcon_HEDY_HEDY

Tan_YPON_YPON_YPON

Pgos_GELE_GELE_PEXI

Bucc_GRAC_BUCC

Doa_NOCT_DOID

Kalb_TINE_PSYC_NARY

Pntp_ZYGA_LIMA_LIMA

Rhmd_TRICHOPTERA

Trca_GEOM_GEOM_LARE

**TAXSET 82txHETEROG.from483tx.nt123 =**

Laenes_INCU_PROD_LAMP

Pcan_INCU_INCU

Pqu_INCU_PROD_PROD

Atr5_INCU_ADEL_ADEL

Ymul_YPON_YPON_YPON

Lpfe_YPON_LYON_LYON

Tyu2_INCU_PROD_PROD

Pxy_YPON_PLUT

Tfu2_INCU_PROD_LAMP

Crin_INCU_CRIN

Nemo_INCU_ADEL_ADEL

Csi_INCU_ADEL_ADEL

Erau_ERIO_ERIO

Nmgs_INCU_ADEL_NEMA

Vqu2_INCU_INCU

Epca_GELE_ELAC_HYPE

Apar_GEOM_GEOM_ARCH

Sdru_GEOM_URNA_EPIP

Cytr_GEOM_GEOM_STER

Pacer_ERIO_checkID

Atth_TISC_TISC

Arot_GELE_GELE_GELE

Eucm_TORT_TORT_OLET

Mcls_GELE_GELE_ANOM

Cpo_TORT_TORT_OLET

Olfa_TORT_TORT_OLET

Eheb_TORT_TORT_OLET

Basp_TORT_TORT_OLET

Imsp_IMMO_IMMI

Cera_TORT_TORT_TORT

Pnex_PYRA_CRAM_PYRA

Eusp_TORT_TORT_OLET

Prap_PAPI_PIER_PIER

Oreo_TINE_PSYC_OIKE

Llun_COSS_COSS_HYPO

Hima_ZYGA_HIMA

Pcoci_TINE_TINE

Dsp_ZYGA_DALC

Acoa_ZYGA_DALC

Moin_TINE_TINE_MYRM

Tco2_TINE_TINE_TINE

Tbi3_TINE_TINE_TINE

Dudg_COSS_DUDG

Micr_MICR_MICR

Damp_PAPI_PIER_DISM

Ding2_ZYGA_DALC

Edvs3_MIMA_MIMA

Anph_ZYGA_LACT

Stpa_GELE_ELAC_STEN

Rhmd_TRICHOPTERA

Enac_NEPT_NEPT_NEPT

Moch_TINE_TINE_MYRM

Aaeq_BOMB_EUPT_EUPT

Cyna_ZYGA_CYCL

Epo2_NEPT_NEPT_NEPT

Abre_TINE_ACRO

Pedy_PAPI_LYCA_PORI

Cotan_BOMB_EUPT

Schrk_SCHR_SCHR

Cycs3_HESP_HESP_PYRG

Tpgt_TINE_TINE_TINE

Svta_ZYGA_LIMA_CHRY

Edos_TINE_TINE_PERI

Pbod_SESI_BRAC_PSEU

Purg_DItrysia

Pcra_TINE_PSYC_PSYC

Comp_TINE_ERIO_COMP

Cosa_TINE_TINE_MYRM

Octg_ZYGA_MEGA

Euds_TINE_TINE_MEES

Aetr_YPON_HELI

Peri_TINE_TINE_PERI

Fhyp_NEPT_NEPT_NEPT

Pquadr2_NEPT_OPOS_OPOS

Hysy_TRICHOPTERA

Hlpu_CALL_CALL_PTER

Haps_TINE_TINE_HAPS

AK154_NEPT_OPOS

Loas_LOPH_LOPH

Mata_ZYGA_DALC

Opns_NEPT_OPOS

Syco_SESI_BRAC

**TAXSET 10txHETEROG.from483tx.degen1 =**

Dhyd_YPON_PLUT

Purg_DItrysia

Szo2_MICR_MICR

Hlpu_CALL_CALL_PTER

Mata_ZYGA_DALC

Pbod_SESI_BRAC_PSEU

Dplex_PAPI_NYMP_DANA

Putr_TINE_TINE_TINE

Opns_NEPT_OPOS

Syco_SESI_BRAC

**TAXSET nonDitrysia =**

Agus_TRICHOPTERA

AK154_NEPT_OPOS

Anla_ANDE_ANDE

Aquen_AGAT_AGAT

Atr5_INCU_ADEL_ADEL

Atth_TISC_TISC

Aun2_ACAN_ACAN

Avld_NEOP_NEOP

Avog_INCU_HELI

Azal_PALA_PALA

Bni2_TRICHOPTERA

Cecid_INCU_CECI

Crin_INCU_CRIN

Csi_INCU_ADEL_ADEL

Dcap_INCU_CECI

Dgr2_ERIO_ERIO

Eate_HEPI_HEPI

Enac_NEPT_NEPT_NEPT

Epo2_NEPT_NEPT_NEPT

Eppd_MICR_MICR

Erau_ERIO_ERIO

Ese2_ERIO_ERIO

Fhyp_NEPT_NEPT_NEPT

Gazm_HEPI_HEPI

Haes_INCU_HELI

Hsp2_HETE_HETE

Hysy_TRICHOPTERA

Kgr13_HEPI_HEPI_HEPI

Laenes_INCU_PROD_LAMP

Loas_LOPH_LOPH

Mac5_MNES_MNES

Mbal_NEPT_NEPT

Mca2_MICR_MICR

Micr_MICR_MICR

Nemo_INCU_ADEL_ADEL

Nmec_NEOP_NEOP

Nmgs_INCU_ADEL_NEMA

Odrp_HEPI_HEPI

Opns_NEPT_OPOS

Ound_HEPI_PALA

Pacer_ERIO_checkID

Pcan_INCU_INCU

Pccc_MICR_MICR

Pctb_TRICHOPTERA

Pect_NEPT_NEPT_PECT

Pohd_HEPI_HEPI

Pqu_INCU_PROD_PROD

Pquadr2_NEPT_OPOS_OPOS

Proto_HEPI_PROT

Ptus_PALA_PALA

Ptys_PALA_PALA

Rhmd_TRICHOPTERA

Seic_TRICHOPTERA

Stan_NEPT_NEPT_NEPT

Szo2_MICR_MICR

Tfu2_INCU_PROD_LAMP

Tgkb_TISC_TISC

Thcn_TISC_TISC

Tpad_NEPT_NEPT_NEPT

Trili_TRICHOPTERA

Trta_HEPI_HEPI

Tyu2_INCU_PROD_PROD

Vqu2_INCU_INCU

Wmo2_TRICHOPTERA

**TAXSET TINE =**

Abre_TINE_ACRO

Arca_TINE_ACRO

Arrp_TINE_ARRH

Ayte_TINE_ACRO_closetoPtcu

Comp_TINE_ERIO_COMP

Cosa_TINE_TINE_MYRM

Dbil_TINE_ARRH

Edos_TINE_TINE_PERI

Ersn_TINE_ERIO

Euds_TINE_TINE_MEES

Exum_TINE_ACRO

Ezeb_TINE_TINE_EREC

Haps_TINE_TINE_HAPS

Hybs_TINE_TINE_MEES

Kalb_TINE_PSYC_NARY

Mbtl_TINE_TINE

Mobu_TINE_TINE_SCAR

Moch_TINE_TINE_MYRM

Moin_TINE_TINE_MYRM

Nard_TINE_PSYC_NARY

Nclo_TINE_TINE_NEMA

Oreo_TINE_PSYC_OIKE

Othi_TINE_TINE_HIER

Pcoci_TINE_TINE

Pcra_TINE_PSYC_PSYC

PdbaPeud_TINE_PSYC_PSEU

Peri_TINE_TINE_PERI

Psasp_TINE_TINE_HIER

Putr_TINE_TINE_TINE

Pymi_TINE_TINE_HIER

Sapp_TINE_TINE_SCAR

Scdy_TINE_PSYC_SCOR

Tbi3_TINE_TINE_TINE

Tco2_TINE_TINE_TINE

Tep2_TINE_PSYC_OIKE

Tpgt_TINE_TINE_TINE

Tycl_TINE_PSYC_TYPH

Xwi_TINE_TINE_MYRM

**TAXSET GRAC.YPON =**

Aetr_YPON_HELI

Agel_GRAC_ROES

Anab_GRAC_GRAC

Asap_YPON_ACRO

Atpu2_YPON_YPON

Bsmu_YPON_BEDE

Bucc_GRAC_BUCC

Caga_GRAC_GRAC_LITH

Cbim_GRAC_GRAC_GRAC

Cdel_GRAC_GRAC_GRAC

Dhem_YPON_ACRO

Dhyd_YPON_PLUT

Dimp_YPON_GLYP

Ehdr_GRAC_GRAC

Emet_YPON_HELI

Epic_GRAC_GRAC_GRAC

Glpx_YPON_GLYP_GLYP

Leuco_YPON_LYON_CEMI

Lpfe_YPON_LYON_LYON

Lpts_YPON_GLYP_GLYP

Opin1_YPON_YPON

Ospa_YPON_GLYP_ORTH

Oure_YPON_YPSO

Pgah_YPON_PLUT

Phcn_GRAC_GRAC_PHYL

Phyl_GRAC_GRAC_LITH

Pmgl_GRAC_GRAC_PHYL

Prbn_GRAC_GRAC_GRAC

Pryo_YPON_YPON_PRAY

Pxy_YPON_PLUT

Rstm_GRAC_ROES

Sput_GRAC_GRAC_GRAC

Sytg_YPON_YPON_SCYT

Tan_YPON_YPON_YPON

Ymul_YPON_YPON_YPON

Yni_YPON_YPSO_YPSO

Ysp_YPON_YPON

**TAXSET Apodit.noMacroPYRA.483tx =**

Ptha_PAPI_NYMP_NYMP

Vane_PAPI_NYMP_NYMP

Dsos_PAPI_NYMP_BIBL

Acly_PAPI_NYMP_APAT

Heli3_PAPI_NYMP_HELI

Dplex_PAPI_NYMP_DANA

Lica_PAPI_NYMP_LIBY

Seur2_PAPI_LYCA_THEC

Pedy_PAPI_LYCA_PORI

Liph_PAPI_LYCA_MILE

Cure_PAPI_LYCA_CURE

Klyd3_PaPI_PIER_COLI

Ceur_PAPI_PIER_COLI

Prap_PAPI_PIER_PIER

Ptdx_PAPI_PIER_PSEU

Damp_PAPI_PIER_DISM

Mcon_HEDY_HEDY

Cet_HESP_HESP_HESP

Cycs3_HESP_HESP_PYRG

Myam_HESP_HESP_PYRR

Hova_HESP_HESP_HETE

Udo_HESP_HESP_PYRG

Hsch_HESP_HESP_COEL

Cram_HESP_HESP_COEL

EurytMibr_PAPI_PAPI_PAPI

Piph3_PAPI_PAPI_PAPI

Anma_PTER_PTER_PTER

Plty_PTER_PTER_PTER

HeinEmon_PTER_PTER_PTER

Adam_PTER_PTER_AGDI

Merid_COPR_CARP

Smim2_COPR_CARP

Cafd_COPR_CARP

Ppra_COPR_COPR

Copro_COPR_COPR

Agdi_PTER_PTER_MACR

Hlpu_CALL_CALL_PTER

Pter_CALL_CALL

Gvii_CALL_CALL_GRIV

Ptfe_CALL_CALL_CALL

Hibd_HYBL_HYBL

Hyfm_HYBL_HYBL

Sski_THYR_THYR_STRI

Dyso_THYR_THYR_THYR

Pfla_THYR_THYR_STRI

Gilt_THYR_THYR_THYR

Lte_THYR_THYR_SICU

Mpll3_THYR_THRY_SICU

Purg_DItrysia

Epchh_EPER_EPER

Esji_EPER_EPER

Epmn_EPER_EPER

Alsp_ALUC_ALUC

Cole_GELE_COSM_BIAS

Nspin_GELE_COLE_BLAS

Stmp_GELE_OECO_STAT

Hiku_GELE_OECO_STAT

ScspSC_GELE_uncertainID

Rham_GELE_XYLO_SCYT

Simm_GELE_XYLO_SCYT

Clph_GELE_COLE_COLE

Win_GELE_OECO_OECO

Lsci_GELE_XYLO_XYLO

Pjez_GELE_ELAC_STEN

Dpd_GELE_ELAC_DEUT

Mom_GELE_COLE_MOMP

Momph_GELE_COLE_MOMP

Gprot2_GELE_AUTO_SYMM

Amod_GELE_AUTO_AUTO

Mqrc_GELE_COSM_COSM

Deoy_GELE_DEOC_DEOC

Illg_GELE_XYLO_XYLO

Tymb_GELE_XYLO_XYLO

Htce_GELE_LECI_LECI

Leci_GELE_LECI_LECI

Epca_GELE_ELAC_HYPE

Hptr_GELE_ELAC_HYPE

Thap_GELE_ELAC_HYPE

Asem_GELE_ELAC_AEOL

Prfx_GELE_AMPH

Agpt_GELE_ELAC_DEPR

Ball_GELE_ELAC_DEPR

Odle_GELE_LECI_ODIT

Rfal_GELE_LECI

Acer_GELE_PELE

Ayth_GELE_GELE

Cpua_GELE_GELE_GELE

Arot_GELE_GELE_GELE

Seto_Ditrysia

Amst_GELE_GELE_ANOM

Mcls_GELE_GELE_ANOM

Etgf_GELE_GELE_DICH

CR19_GELE_checkID

Pgos_GELE_GELE_PEXI

Dpunc_GELE_GELE_DICH

Cill_GELE_ELAC_ELAC

Eten_GELE_ELAC_ELAC

Hlch_GELE_BATR_BATR_sameasBatr

Htur_GELE_COSM

Cosm_GELE_COSM_COSM

Panla_GELE_COSM_ANTE

Fbrn_GELE_ELAC_DEPR

Eeu_GELE_ELAC_ETHM

Stpa_GELE_ELAC_STEN

Aren_GELE_ELAC_STEN

Dfag_GELE_CHIM

Lmau_TINE_LYPU

Phih_ZYGA_LIMA_LIMA

Pdbi_ZYGA_LIMA_LIMA

Edel3_ZYGA_LIMA_LIMA

Svta_ZYGA_LIMA_CHRY

Dsp_ZYGA_DALC

Acoa_ZYGA_DALC

Ding2_ZYGA_DALC

Mata_ZYGA_DALC

Pntp_ZYGA_LIMA_LIMA

Lcr2_ZYGA_MEGA_MEGA

Mglp_ZYGA_MEGA

Octg_ZYGA_MEGA

Nora_ZYGA_MEGA

Mdyi_ZYGA_MEGA_TROS

Aios_ZYGA_AIDI

Hima_ZYGA_HIMA

Posp_ZYGA_ZYGA_PROCmaybe

Etsp_ZYGA_ZYGA_CHAL

Psin_ZYGA_ZYGA_PHAU

Zgfa_ZYGA_ZYGA_ZYGA

Phau_ZYGA_ZYGA_PHAU_problemID

Anph_ZYGA_LACT

Lsub_ZYGA_LACT

Lact_ZYGA_LACT_uncertainID

Amca_SESI_CAST

Tlic_SESI_CAST_CAST

Pays_SESI_CAST_CAST

Spla_SESI_CAST

Pfoc_COSS_COSS_HYPO

Gmuc_COSS_COSS_HYPO

Llun_COSS_COSS_HYPO

Mnau_COSS_COSS_META

Lebe_COSS_COSS_META

Eeny_COSS_COSS_ZEUZ

Ppls_COSS_COSS_ZEUZ

Xmns_COSS_COSS_ZEUZ

Zcof_COSS_COSS_ZEUZ

Enaw_ZYGA_EPIP_EPIP

Esp2_ZYGA_EPIP

Heter_ZYGA_EPIP

Cosla_COSS_COSS_COSS

Cvdv_COSS_COSS_COSS

Cucr_COSS_COSS_COSS

Coss_COSS_COSS_COSS

Prob2_COSS_COSS_COSS

Pbod_SESI_BRAC_PSEU

Dudg_COSS_DUDG

Ahss_COSS_COSS

Psy2_SESI_SESI_SESI

Syex_SESI_SESI_SESI

Mcuc2_SESI_SESI_SESI

Vipo_SESI_SESI_PARA

Phms_SESI_SESI_TINT

Cpo_TORT_TORT_OLET

Gdel_TORT_TORT_OLET

Eucm_TORT_TORT_OLET

Basp_TORT_TORT_OLET

Eusp_TORT_TORT_OLET

Ancy_TORT_TORT_OLET

Etyr_TORT_TORT_OLET

Eheb_TORT_TORT_OLET

Olfa_TORT_TORT_OLET

Pasma_TORT_TORT_OLET

Etbk_TORT_TORT_TORT

Aesp_TORT_TORT_TORT

Eutr_TORT_TORT_TORT

Bogo_TORT_TORT_TORT

Calfa_TORT_TORT_CNEP

Dasa_Tort_TORT_TORT

Cler_TORT_TORT_TORT

Anne_TORT_TORT_TORT

Cros_TORT_TORT_TORT

Arga_TORT_TORT_TORT

Cera_TORT_TORT_TORT

Pida2_TORT_TORT_TORT

Srtc_TORT_TORT_TORT

Pasp_TORT_TORT_TORT

Audi_TORT_TORT_CHLI

Htmn_TORT_TORT_CHLI

Hila_TORT_TORT_CHLI

Hist_TORT_TORT_CHLI

Pvol_TORT_TORT_CHLI

Syco_SESI_BRAC

AK142_SESI_BRAC

Hmnt_TORT_HELI

Cyna_ZYGA_CYCL

Cysp_ZYGA_CYCL

Hani_GALA_GALA

Imsp_IMMO_IMMI

Bcle_IMMO_IMMI

Imja_IMMO_IMMI

Mido_CHOR_CHOR_MILLI

Tmic_CHOR_CHOR_CHOR

Poeu_CHOR_CHOR_CHOR

Anfa_CHOR_CHOR_CHOR

Hfel_CHOR_CHOR

Tort_CHOR_CHOR_CHOR

Bren_CHOR_CHOR_BREN

Schrk_SCHR_SCHR

Sktn_SCHR_SCHR

Ktr_GRAC_DOUG

Ursp_UROD_UROD

Wasp_UROD_UROD

Woc_UROD_UROD
